# Analysis of three different reverse shoulder arthroplasty designs for cuff tear arthropathy – the combination of lateralization and distalization provides best mobility

**DOI:** 10.1186/s12891-024-07312-5

**Published:** 2024-03-07

**Authors:** Florian Freislederer, Philipp Moroder, Laurent Audigé, Tim Schneller, Yacine Ameziane, Raphael Trefzer, Jan-Philipp Imiolczyk, Markus Scheibel

**Affiliations:** 1 Schulthess Clinic, Department of Shoulder and Elbow Surgery, Zurich, Switzerland; 2 Department of Research and Development, Upper Extremities, Schulthess Clinic, Zurich, Switzerland; 3 Center for Musculoskeletal Surgery, Charité-Universitaetsmedizin, Berlin, Germany; 4 Surgical Outcome Research Center, Department of Clinical Research, University Hospital Basel and University of Basel, Basel, Switzerland

**Keywords:** Reverse shoulder arthroplasty, Reverse, Lateralization, Grammont, Design, Shoulder arthroplasty

## Abstract

**Background:**

The two major reverse shoulder arthroplasty (RSA) designs are the Grammont design and the lateralized design. Even if the lateralized design is biomechanically favored, the classic Grammont prosthesis continues to be used. Functional and subjective patient scores as well as implant survival described in the literature so far are comparable to the lateralized design. A pure comparison of how the RSA design influences outcome in patients has not yet been determined. The aim of this study was a comparison focused on patients with cuff tear arthropathy (CTA).

**Methods:**

We analyzed registry data from 696 CTA patients prospectively collected between 2012 and 2020 in two specialized orthopedic centers up to 2 years post-RSA with the same follow-up time points (6,12 24 months). Complete teres minor tears were excluded. Three groups were defined: group 1 (inlay, 155° humeral inclination, 36 + 2 mm eccentric glenosphere (n = 50)), group 2 (inlay, 135° humeral inclination, 36 + 4 mm lateralized glenosphere (n = 141)) and group 3 (onlay, 145° humeral inclination, + 3 mm lateralized base plate, 36 + 2 mm eccentric glenosphere (n = 35)) We compared group differences in clinical outcomes (e.g., active and passive range of motion (ROM), abduction strength, Constant-Murley score (CS)), radiographic evaluations of prosthetic position, scapular anatomy and complications using mixed models adjusted for age and sex.

**Results:**

The final analysis included 226 patients. The overall adjusted *p*-value of the CS for all time-points showed no significant difference (*p* = 0.466). Flexion of group 3 (mean, 155° (SD 13)) was higher than flexion of group 1 (mean, 142° (SD 18) and 2 (mean, 132° (SD 18) (*p* < 0.001). Values for abduction of group 3 (mean, 145° (SD 23)) were bigger than those of group 1 (mean, 130° (SD 22)) and group 2 (mean, 118° (SD 25)) (*p* < 0.001). Mean external rotation for group 3 (mean, 41° (SD 23)) and group 2 (mean, 38° (SD 17)) was larger than external rotation of group 1 (mean, 24° (SD 16)) (*p* < 0.001); a greater proportion of group 2 (78%) and 3 (69%) patients reached L3 level on internal rotation compared to group 1 (44%) (*p* = 0.003). Prosthesis position measurements were similar, but group 3 had significantly less scapular notching (14%) versus 24% (group 2) and 50% (group 1) (*p* = 0.001).

**Conclusions:**

Outcome scores of different RSA designs for CTA revealed comparable results. However, CTA patients with a lateralized and distalized RSA configuration were associated with achieving better flexion and abduction with less scapular notching. A better rotation was associated with either of the lateralized RSA designs in comparison with the classic Grammont prosthesis.

**Level of Evidence:**

Therapeutic study, Level III.

**Supplementary Information:**

The online version contains supplementary material available at 10.1186/s12891-024-07312-5.

## Introduction

The physiological function of the shoulder joint is dependent on an intact rotator cuff. Cuff tear arthropathy (CTA) is defined by advanced damage of the rotator cuff that leads to successive arthritic degeneration with radiologically classified signs of structural osseous modifications [1]. Besides cranialization of the humerus with reduced acromiohumeral distance, erosion of the humeral head and superior glenoid surface leaves distinct joint alterations with a medialized joint line and poor function [2].

Reverse shoulder arthroplasty (RSA) is a widespread treatment option for irreparable rotator cuff deficiency and associated osteoarthritic joint degeneration. The first RSA concept which became widely used for patients with CTA was presented by Paul Grammont. He used a medialized and distalized design to create a stable fulcrum around which the humerus could rotate and provided enough delta tension to enable very good elevation and abduction movements [3]. With the rising recognition of associated complications of this concept such as inferior scapular notching and unsatisfying outcomes in axial rotation, modified reversed arthroplasty designs were developed [2, 4–7]. Reduction of the humeral neck-shaft angle (NSA) and lateralization of the center of rotation aim to reduce conflict at the scapula neck. A reduced NSA improves impingement-free range of motion (ROM) and axial motion by creating a more anatomical vector and more tension of the remaining anterior and posterior rotator cuff muscles [8–11]. Mark Frankle popularized a bipolar lateralization with a lateralized glenosphere and reduced NSA of 135° (compared to the 155° of the Grammont design) [10].

Generally, it has be to be said that in all RSA designs the center of rotation remains medialized in comparison to a native glenoid joint. The terminus “lateralized” refers to more lateralized compared to the original Grammont (“more medialized”) design.

The advantages of a glenoidal lateralization were kept in further design evolutions [12]. On the humeral side, onlay systems for humeral lateralization were introduced [13]. Furthermore, the NSA shifted towards a way in between the Grammont and Frankle concept aiming to gather the advantages of a distalized and a lateralized concept [14]. Therefore, various humeral designs with an NSA of 145° were introduced [15].

The influence of various lateralized designs on clinical outcome has been widely reviewed and several advantages over medialized RSAs have been outlined such as decreased inferior scapular notching, better stability, and rotational mobility [8, 11, 16–18]. Nonetheless, the more recent reviews were unable to highlight any significant differences in shoulder function and outcome scores [11, 16]. There is a lack of evidence on how different RSA designs (the Grammont design, the Frankle design or a distalized and lateralized design) perform in comparable patient populations; this knowledge would improve the surgeon’s choice of prosthesis design based on specific indications. Our purpose was to compare these three concepts, with regards to clinical and radiographic outcome in a homogeneous cohort of patients with CTA. We hypothesized that by lateralizing and distalizing, better outcome scores and superior ROM as well as reduced notching would be achieved. The analyzed outcomes were ROM, a radiological core set evaluation [16] and outcome scores (CS, SPADI).

## Materials and methods

### Patient selection

This is a retrospective cohort study on patients with CTA who were treated with one of three different RSA prostheses at one of two specialized orthopedic centers. Since June 2012 all patients receiving a shoulder arthroplasty at one center (KWS) were prospectively documented in a local register. At the second center (BER), all patients were prospectively documented since June 2016. Trained specialized shoulder surgeons performed the operation at both centers. From both databases, patients with CTA were selected for this analysis when they had complete preoperative and 2-year clinical and radiographic examinations and were treated with one of the following implants: 1. Aequalis Reversed II prosthesis with 155° neck-shaft inclination and 36 + 2 mm eccentric glenosphere (Wright Medical Group N.V., Memphis, TN) (Group 1, medialized and distalized concept). 2. Univers Revers II prosthesis with 135° neck-shaft inclination and 36 + 4 mm lateralized glenosphere (Arthrex, Naples, FL) (Group 2, lateralized concept) or 3. Aequalis Ascend Flex prosthesis with 145° neck-shaft inclination, + 3 mm lateralized baseplate and 36 + 2 mm eccentric glenosphere (Wright Medical Group N.V., Memphis, TN) (Group 3, lateralized and distalized concept)). Based on the three prosthesis types, the theoretical global lateralized offset (tGLO) is 15.6 mm, 24.7 mm and 27.5 mm for groups 1, 2 and 3, respectively [12]. In addition, only data from the first operated side per patient were analyzed. Patients diagnosed with a complete teres minor tear were excluded. This analysis used prospectively documented clinical data that was approved by the local ethics committee for research purposes.

### Surgical technique and postoperative protocol

All reverse prostheses were implanted according to manufacturer instructions by mainly 4 and in total 7 experienced shoulder surgeons. A deltopectoral approach was used and tenotomy of the subscapularis (SSC) tendon was performed followed by circular capsulotomy. The tendon of the long head of the biceps, if still intact, was tenotomized. The humeral head was resected by all surgeons consistenly with 20° retroversion. After preparation of the humeral shaft the glenoid was exposed and remaining cartilage and labrum were removed. The central drill wire was inserted, and the central peg channel was drilled. The baseplate was placed centrally (group 2) or more flush to the inferior border of the glenoid (group 2 and 3) and fixed with two head locking and compression screws each for group 1 and a central bicortical screw followed by four peripheral screws for the group 3. The baseplate of group 2 patients was inserted and fixed with a central and two peripheral screws followed by peripheral over-reaming of the circumferential bone; the eccentric glenosphere was positioned and secured with a locking screw connection to the baseplate. In Onlay type prosthesis the humeral cut might have been slightly deeper sometimes, depending on the tension (that is higher in onlay type of designs), but for all patients the initial cut was at the anatomical neck and a recut was done depending on the intraoperative individual surgeon's decision.

After testing the RSA reduction and stability with trial implants, the definitive implant was inserted and tested again for impingement-free mobility. The SSC was reattached with FiberWire® sutures (Arthrex, Naples, FL) using the Mason-Allen technique.

Patients were required to keep their arm immobilized in a sling for 4 weeks after surgery while following a standardized physical therapy program starting from Day 1. Passive mobilization the first 4 weeks post-surgery followed by active-assisted mobilization. By the sixth postoperative week, patients were allowed to apply progressive active motion. Internal rotation against resistance was avoided for the first 6 weeks.

### Clinical evaluations

Patients underwent clinical examination preoperatively (baseline) and at 6-, 12- and 24 months after surgery, at 6 months mostly by the surgeons, at 12 and 24 months by independent observers. Clinical parameters of shoulder ROM (included elevation, abduction, internal and external rotation at 90° abduction, external rotation at 0° abduction, capacity of internal rotation (using the Apley scratch test) at 0° abduction and shoulder strength in 90° abduction were assessed. Functional outcome was based on the Constant-Murley score (CS) [19, 20], Subjective Shoulder Value (SSV) [20] and the patient-reported Shoulder Pain and Disability Index (SPADI) [21].

### Radiological baseline and 2-year follow-up parameters

**Fig. 1:**
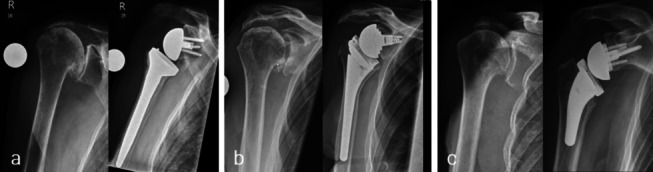
Prosthetic designs (ap x-rays pre- and 2yrs postoperative): **a** Group 1; NSA:155°, Inlay, GS: 36 + 2 mm ecc, **b** Group 2; NSA:135°, Inlay, GS: 36 + 4 mm lat **c** Group 3; NSA:145°, Onlay, BP: + 3 mm lat, GS: 36 + 2 mm ecc

**Fig. 2:**
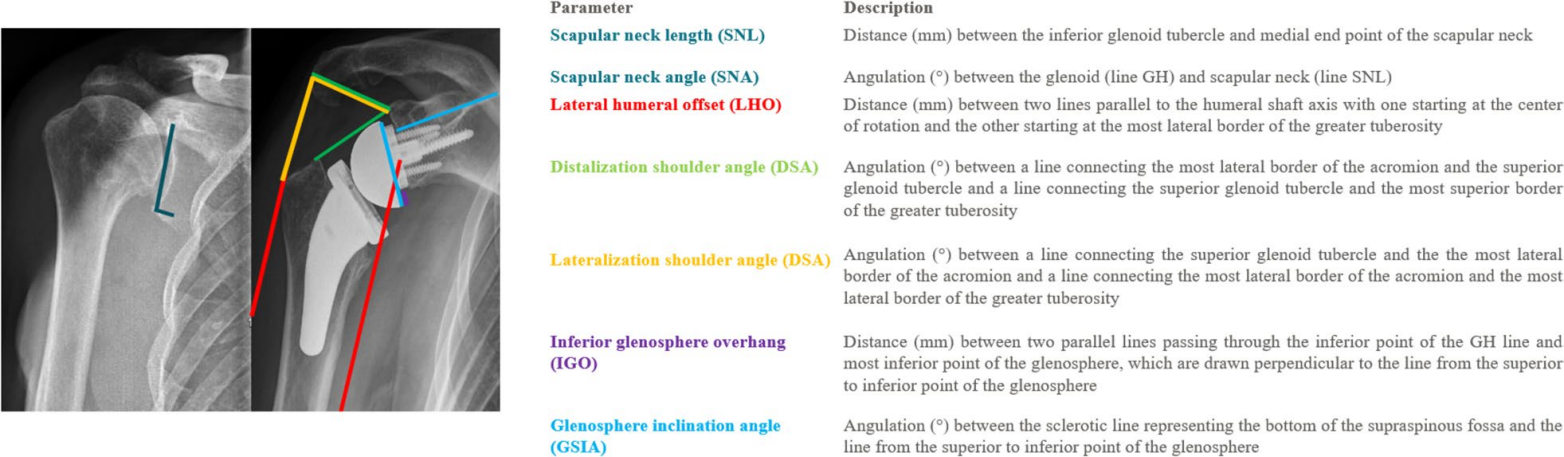
**a** Preoperative anteroposterior radiograph highlighting scapular neck length as indicated by the blue line (long) and neck angle lying between the two blue lines **(∡)**;**b** colored lines featured on the postoperative image indicate various radiological measurements of prosthetic position, i.e., lateral humeral offset (red), distalization shoulder angle (green), inferior glenoid overhang (purple) and glenosphere inclination angle (blue)

Baseline (preoperative) and 2-year postoperative radiographs included standard anteroposterior (Fig. 1) and axial views. From anteroposterior images, a range of parameters were assessed at both time points to provide details on scapular anatomy and prosthetic position (Fig. 2). Scapular anatomy was defined by scapular neck length (SNL) and angle (SNA), where SNL is the distance (mm) between the inferior glenoid tubercle and medial end point of the scapular neck and SNA is the angle (º) between the glenoid and scapular neck length (SNL). Prosthetic position was described by the following parameters of lateral humeral offset (LHO), distalization shoulder angle (DSA), lateralization shoulder angle (LSA), inferior glenosphere overhang (IGO) and glenosphere inclination angle (GSIA): LHO is the distance (mm) between two lines (red) parallel to the humeral shaft axis with one starting at the superior glenoid tubercle and the other starting at the most lateral border of the greater tuberosity; DSA lies between a line (green) connecting the most lateral border of the acromion and the superior glenoid tubercle and a line (green) connecting the superior glenoid tubercle and the most superior border of the greater tuberosity; IGO indicates the distance (mm) between the inferior point of the glenohumeral line and most inferior point of the glenosphere; and GSIA lies between the sclerotic line (blue) representing the bottom of the supraspinatus fossa and the line (blue) from the superior to inferior point of the glenosphere. The degree of baseline glenoid erosion was assessed using the established Favard classification system [2].

Throughout the 2-year postoperative period, various adverse events of inferior scapular notching based on the Sirveaux classification [2], signs of osteolysis around the implant components, ossification, component migration or breakage, and periprosthetic fractures were documented based on an international consensus core set [22].

### Data management and statistical analysis

Register data were managed using the REDCap (Research Electronic Data Capture) system [23] and exported for statistical analysis using Intercooled Stata version 17 (StataCorp LP, College Station, TX). Baseline patient demographic, radiological and functional parameters were tabulated separately per group using standard descriptive statistics and compared using standardized differences (where values closest to 0.10 indicate stronger group similarity) [24] and clinical judgment. Comparative analyses at the 2-year postoperative follow-up were conducted using standard linear regression analyses, and we used generalized linear mixed models to account for repeated measurements when outcome data were available at each clinical follow-up examination, as applicable. For all models, we included the demographic parameters of age and sex as well as respective baseline preoperative values. All eligible patients from the two databases were included, so there was no predetermined sample size based on comparative analyses; all analyses were explorative with a significance level set at 0.05.

## Results

**Fig. 3:**
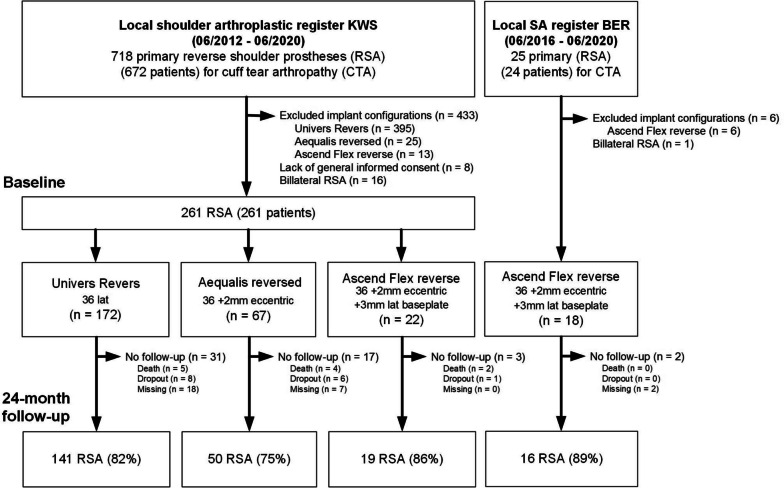
Flowchart for patient selection

Between June 2012 and June 2020, there were 67, 172 and 40 RSA in the groups 1, 2 and 3, respectively. At the 2-year postoperative follow-up, 50, 141 and 35 patients respectively, met the inclusion criteria and were selected for this analysis (Fig. 3). Most patients were women and the average age at the time of surgery was 76 years (range 52–93). The three defined groups shared similar baseline characteristics (except for a higher proportion of female patients in group 2, a higher proportion of low-grade Hamada 1 CTA (Table 1)), and preoperative shoulder function (Table 2).

**Table 1:** Baseline patient and shoulder characteristics according to the defined prosthesis groups

	Group 1		Group 2		Group 3		StdDiff	
	n(%^a^)	mean (SD)	n(%^a^)	mean (SD)	n(%^a^)	mean (SD)		1vs.3/ 1vs.2 / 2vs.3
Age at surgery		74.4 (7.7)		75.5 (5.7)		75.7 (6.2)		0.189 / 0.165 / 0.033
Sex								0.847 / 0.055 / 0.789
Female	33(66)		136(96)		24(69)			
Male	17(34)		5(4)		11(31)			
Diagnosis								0.563 / 0.117 / 0.148
RC tear without arthrosis	9(18)		27(19)		11(31)			
RC tear with arthrosis	41(82)		114(81)		24(69)			
Radiological examination								0.281 / 0.630 / 0.452
None	3(9)		8(8)					
Magnetic resonance imaging	20(63)		86(83)		23(100)			
Ultrasound	9(28)		9(9)					
RC tear type according to Lädermann^b^								0.205 / 0.396 / 0.446
A	3(10)		17(18)		4(17)			
B	1(3)		3(3)		2(9)			
C	18(60)		50(53)		14(61)			
D	8(27)		23(24)		3(13)			
E			1(1)					
Supraspinatus								0.099 / 0.820 / 1.064
Intact tendon	1(3)							
Partial tear	1(3)		16(17)					
Complete tear	27(93)		80(83)		23(100)			
Infraspinatus								0.108 / 0.306 / 0.311
Intact tendon	7(24)		23(24)		2(8)			
Partial tear	10(34)		32(33)		9(39)			
Complete tear	12(41)		41(43)		12(52)			
Subscapularis								0.211 / 0.432 / 0.379
Intact tendon	9(31)		28(29)		8(34)			
Partial tear	16(55)		54(56)		11(48)			
Complete tear	4(14)		14(15)		4(17)			
Teres minor								0.179 / 0.219 / 0.151
Intact	28(97)		88(92)		19(83)			
Partial tear			6(6)		3(12)			
n.d	1(3)		2(2)		2(8)			
Glenoid wear according to Favard^c^								0.688 / 0.205 / 0.151
E0	21(44)		29(22)		15(42)			
E1	4(8)		29(22)		7(19)			
E2	7(16)		13(10)		1(4)			
E3	4(8)		6(4)		10(30)			
n.d	11(24)		56(42)		2(7)			
RC tear arthropathy according to Hamada^d^								0.544 / 0.861 / 1.054
Grade 1	13(28)		72(54)		7(19)			
Grade 2	9(19)		17(13)		7(19)			
Grade 3	6(13)		7(5)		5(15)			
Grade 4A	4(9)		11(8)		2(6)			
Grade 4B	9(19)		18(14)		5(15)			
Grade 5			4(3)		7(20)			
n.d			4(3)		2 (6)			

*SD* standard deviation, *StdDiff* standardized difference calculated to three decimal places and equal to the absolute difference between group means divided by the common standard deviation, where values closest to 0.10 or below indicate stronger group similarity. The three values show the standardized difference between groups 155 and 135, groups 155 and 145, and groups 145 and 135, respectively; RC = rotator cuff; n.d. = indeterminable

^a^The percentage refers to the number of patients missing excluded

^b^Lädermann A, Denard PJ, Collin P. Massive rotator cuff tears: definition and treatment. Int. Orthop*.* 2015;39(12):2404–2414. https://doi.org/10.1007/s00264-015-2796-5: A = supraspinatus and superior subscapularis tears, B = supraspinatus and entire subscapularis tears, C = infraspinatus, supraspinatus and superior subscapularis tears, D = supraspinatus and infraspinatus tears, E = supraspinatus, infraspinatus and teres minor tears

^c^Favard L, Lautmann S, Sirveaux F, Oudet D, Kerjean Y, Huguet D. Hemiarthroplasty versus reverse arthroplasty in the treatment of osteoarthritis with massive rotator cuff tear. In: Walch G, Boileau P, Molé D, editors. 2000 Shoulder Prostheses. Two to ten years follow-up. Sauramps Medical: Paris, France; 2001. p 261–268: E0 = superior humeral head migration without erosion of the glenoid, E1 = concentric erosion of the glenoid, E2 = if erosion was limited to the superior part of the glenoid, E3 = if erosion extended to the inferior part of the glenoid

^d^Hamada K, Fukuda H, Mikasa M, Kobayashi Y. Roentgenographic findings in massive rotator cuff tears. A long-term observation. Clin Orthop Relat Res. 1990 May(254):92–96: 1 = acromiohumeral interval > 6 mm; normal glenohumeral joint, 2 = acromiohumeral interval < 5 mm; normal glenohumeral joint, 3 = acromiohumeral interval < 5 mm, with acetabulization of acromion; normal glenohumeral joint, 4A = glenohumeral osteoarthritis without acetabulization, acromiohumeral interval < 7 mm, 4B = glenohumeral osteoarthritis with acetabulization, acromiohumeral interval < 7 mm, 5 = humeral head subchondral collapse characteristic of cuff tear arthropathy

**Table 2:** Baseline and postoperative shoulder range of motion (ROM) parameters, strength and functional scores

	**Group 1**			**Group 2**				**Group 3**				Adjusted		Model
	**n**		**mean (SD)**	**n**		**mean (SD)**		**n**		**mean (SD)**		***p*** **-value**		***p*** -value*
Active motion parameters														
Flexion (°)														< 0.001
Baseline	50		71 (33)	141		78 (39)		35		69 (36)				
6 months	47		135 (20)	130		125 (25)		14		136 (21)		0.108		
12 months	43		141 (18)	123		130 (23)		22		146 (20)		0.002		
24 months	40		142 (18)	107		132 (18)		33		155 (13)		< 0.001		
Abduction (°)														< 0.001
Baseline	50		65 (25)	141		70 (34)		35		62 (31)				
6 months	47		123 (23)	129		118 (28)		14		120 (31)		0.949		
12 months	43		128 (22)	123		124 (26)		22		138 (25)		0.061		
24 months	40		130 (22)	107		118 (25)		33		147 (23)		< 0.001		
External rotation in 0° abd. (°)														< 0.001
Baseline	48		30 (18)	141		31 (22)		35		22 (23)				
6 months	47		22 (11)	131		33 (14)		14		41 (25)		< 0.001		
12 months	43		23 (11)	123		35 (15)		22		38 (21)		< 0.001		
24 months	40		24 (16)	107		38 (17)		33		41 (23)		< 0.001		
Passive motion parameters														
Flexion passive (°)														
Baseline	50		88 (39)	141		97 (39)		35		94 (45)				< 0.001
6 months	47		142 (19)	130		128 (22)		14		146 (18)		0.001		
12 months	43		149 (18)	123		133 (20)		22		157 (15)		< 0.001		
24 months	40		147 (18)	107		135 (17)		33		163 (11)		< 0.001		
Abduction passive (°)														
Baseline	50		77 (34)	141		85 (38)		35		90 (44)				< 0.001
6 months	47		131 (21)	129		121 (25)		14		130 (32)		0.237		
12 months	43		135 (21)	123		125 (24)		22		151 (20)		< 0.001		
24 months	40		136 (21)	107		121 (24)		33		156 (21)		< 0.001		
External rotation in 0° abd. passive (°)														
Baseline	46		35 (18)		139	38 (23)	35		31 (24)				< 0.001	
6 months	47		30 (9)		131	34 (13)	14		50 (22)		< 0.001			
12 months	43		31 (12)		122	36 (14)	22		46 (18)		< 0.001			
24 months	40		35 (14)		107	43 (14)	33		50 (23)		< 0.001			
Passive motion parameters														
Flexion passive (°)														
Pre-op	77		90 (35)		190	96 (36)	60		89 (40)				< 0.001	
6 months	71		140 (20)		176	131 (22)	23		147 (20)		< 0.001			
12 months	67		148 (18)		164	135 (19)	38		156 (22)		< 0.001			
24 months	65		145 (18)		141	137 (17)	57		162 (16)		< 0.001			
Abduction passive (°)														
Pre-op	77		78 (30)		190	83 (34)	60		81 (39)				< 0.001	
6 months	71		129 (21)		175	123 (25)	23		134 (30)		0.118			
12 months	67		133 (20)		164	129 (24)	38		151 (27)		< 0.001			
24 months	65		134 (21)		141	125 (24)	57		156 (23)		< 0.001			
External rotation in 0° abd. passive (°)														
Pre-op	70		32 (17)		187	35 (22)	59		26 (22)				< 0.001	
6 months	71		30 (11)		176	34 (13)	23		49 (19)		< 0.001			
12 months	67		32 (12)		161	36 (14)	38		42 (18)		< 0.001			
24 months	65		36 (14)		141	43 (14)	57		48 (21)		< 0.001			
Strength, pain level and functional scores														
Strength in abduction (kg)													0.768	
Baseline		50	0.4 (0.9)		141	0.4 (1.1)	19		0.3 (1.2)					
6 months		45	4.2 (2.3)		130	3.3 (1.8)	14		3.8 (2.3)		0.669			
12 months		42	4.7 (2.3)		120	3.9 (2.0)	9		4.4 (2.3)		0.950			
24 months		39	5.3 (2.4)		106	3.9 (2.0)	17		4.8 (2.2)		0.627			
Pain NRS (0 = no pain, 10 = maximum pain)													0.008	
Baseline		46	6.1 (3.0)		129	6.4 (2.5)	33		6.2 (2.7)					
6 months		49	1.4 (1.7)		129	1.6 (1.9)	15		0.7 (0.9)		0.348			
12 months		44	1.4 (1.7)		129	1.3 (1.9)	27		0.6 (1.2)		0.055			
24 months		47	1.7 (2.1)		128	1.5 (2.1)	34		0.6 (1.2)		0.032			
CS Constant Murley Score (0–100 = best)													0.466	
Baseline		42	27 (11)		122	31 (15)	20		30 (14)					
6 months		40	65 (12)		114	62 (13)	11		64 (10)		0.576			
12 months		38	69 (10)		105	67 (13)	21		70 (10)		0.533			
24 months		33	68 (11)		83	68 (10)	31		76 (9)		0.088			
SPADI (0 = worst, 100 = best)													0.252	
Baseline		46	33 (22)		128	34 (20)	18		40 (19)					
6 months		50	73 (20)		131	78 (18)	15		84 (14)		0.199			
12 months		44	76 (19)		130	82 (18)	14		83 (14)		0.598			
24 months		47	74 (21)		129	80 (20)	17		83 (15)		0.534			
Subjective Shoulder Value (0 = worst, 100 = best)													0.325	
Baseline		38	41 (20)		118	39 (20)	34		34 (20)					
6 months		41	74 (18)		112	78 (16)	12		78 (13)		0.856			
12 months		41	75 (18)		117	83 (13)	26		78 (14)		0.110			
24 months		40	77 (18)		114	83 (14)	34		85 (13)		0.323			

*SD* standard deviation

^*^Mixed model *p*-value for group effect adjusted for age, gender and baseline pre-operative values

### Clinical examination and patient-reported outcomes

Two-years post-RSA, flexion of group 3 (mean, 155° (SD 13)) was higher than flexion of group 1 (mean, 142° (SD18) and group 2 (mean, 132° (SD18) (*p* < 0.001). Abduction of group 3 (mean, 145° (SD 23)) was also higher than abduction of group 1(mean, 130° (SD22)) and group 2 (mean, 118° (SD25)) (*p* < 0.001). Mean external rotation for group 3 (mean, 41° (SD 23)) and group 2 (mean, 38° (SD17)) was larger than external rotation of group 1 (mean, 24° (SD 16)) (*p* < 0.001); a greater proportion of group 2 (78%) and 3 (69%) patients reached L3 level for internal rotation compared to group 1 (44%) (*p* = 0.003).

**Fig. 4:**
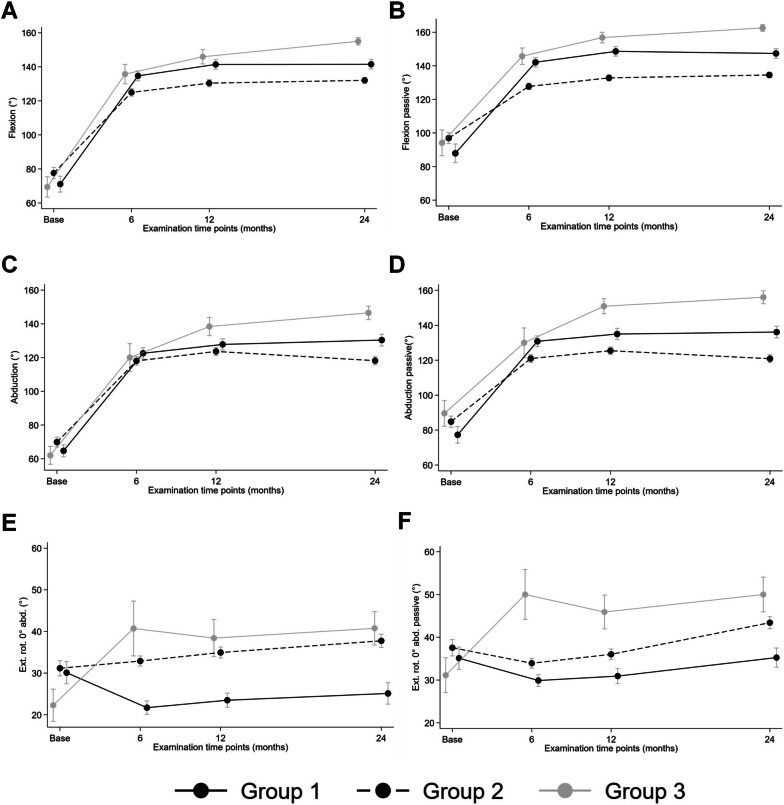
Graphics of active and passive ROM at various time points

**Fig. 5:**
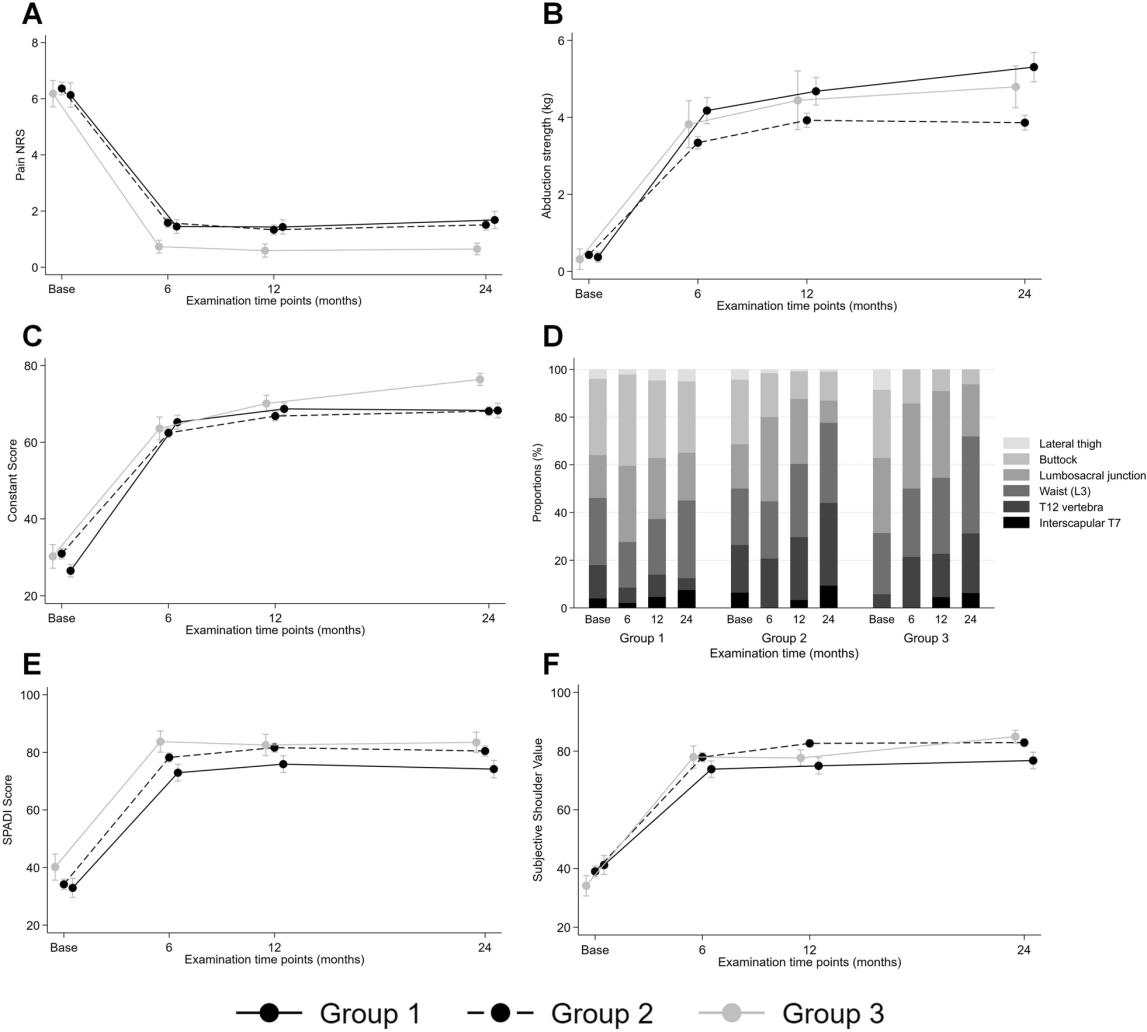
Graphics of Outcome scores (CS, SPADI) pain, internal rotation (Apley Scratch test) and abduction strength at various time points

Group 3 patients had significantly better ROM compared to patients in groups 1 and 2 (Table 3): group 3 patients achieved an average anterior flexion of 155°, which was 15° (95% confidence interval [CI] 7° to 23°) and 23° (CI 16° to 30°) better than groups 1 and 2 (*p* < 0.001) (Fig. 4). Mean abduction for group 3 was 147°, 19° (CI 8° to 30°) higher compared to group 1 and 28° (CI 17° to 38°) better than group 2 (*p* < 0.001) (Fig. 4). The low mean external rotation achieved by group 3 (41°) was 18° (CI 11° to 26°) higher than group 1 and 7° (CI 1° to 14°) higher than group 2 (*p* < 0.001) (Fig. 4); this difference was due to a better active external rotation (Fig. 4). Greater proportions of group 2 (78%) and 3 (69%) patients were able to reach the lumbar vertebrae 3 (L3) compared to group 1 (43%) (*p* = 0.003) (Fig. 5).

**Table 3:** Comparison of baseline scapula anatomy and 2-year postoperative prosthesis position measurements between defined study groups

	Group 1		Group 2		Group 3		StdDiff
	n	mean (SD)	n	mean (SD)	n	mean (SD)	1vs.3 / 1vs.2 / 2vs.3
Scapular anatomy							
Scapular neck length (mm)	49	14.8 (11.7)	135	13.1 (6.4)	33	13.6 (4.6)	0.02 / 0.03 / 0.01
Scapular neck angle (º)	49	82.4 (13.5)	135	83.2 (11.8)	33	85.1 (12.3)	0.04 / 0.01 / 0.04
							*P*-value
Prosthesis position							
Lateral humeral offset (mm)	49	33.1 (8.1)	135	40.9 (4.8)	33	44.0 (4.5)	< 0.001
Distalization shoulder angle (º)	49	52.2 (10.8)	135	45.5 (10.5)	33	52.0 (8.2)	< 0.001
Inferior glenosphere overhang (mm)	49	5.9 (12.5)	135	2.8 (2.2)	33	5.8 (1.9)	0.002
Glenosphere inclination angle (º)	49	98.2 (9.0)	135	102.3 (7.9)	33	101.4 (7.6)	0.013
Lateralization Shoulder Angle (°)	49	78.4 (10.4)	135	87.4 (9.6)	33	83.9 (7.4)	< 0.001

*SD* standard deviation, *StdDiff* standardized difference calculated to two decimal places and equal to the absolute difference between group means divided by the common standard deviation, where values closest to 0.10 or below indicate stronger group similarity. The three values show the standardized difference between groups 155 and 135, groups 155 and 145, and groups 145 and 135, respectively

Outcome scores, adjusted for baseline values, showed no significant differences at follow-up (Table 3; e.g. CS: *p* = 0.466). At 2 years the baseline- and gender-adjusted Constant score was on average 3 points higher (CI -3 to 9) for group 3 (76 points; range 56–96) in comparison with group 2 (68 points; range 26–85) and 7 points (CI 1 to 13) compared to group 1 (68 points; range 40–85 points), an observation however that showed only a statistical trend but no significance (*p* = 0.088) (Fig. 5). Mean outcomes of SSV and SPADI were also not significantly different between groups (*p* = 0.325 and *p* = 0.225) (Fig. 5).

### Radiological outcomes

All groups shared similar baseline measurements of SNL and SNA (Table 3). At the 2-year follow-up, there was a statistically significant difference in GSIA (*p* = 0.013), but mean LHO was significantly lower in group 1 (33 mm) and group 2 showed a lower mean IGO (3 mm) (*p* < 0.001) (Table 3).

There was significantly less scapular notching (14%) reported for group 3 compared to groups 2 (24%) and 1 (50%) (*p* = 0.001). Grade 1 notching was reported in 50% group 1 patients, in 14% group 3 patients and in 20% group 2 patients; Grade 2 notching was only found in 4% of group 2 patients. Overall, we did not report any signs of osteolysis, radiolucency, bone resorption, ossification, implant migration/breakage/loosening for any of the study patients. There were 2 acromial fractures (Levy type 2) in group 2 and one (Levy type 2) in group 1, all of which did not need surgical treatment.

## Discussion

Our retrospective study shows that the baseline/gender-adjusted CS difference for all groups comprising all-time points was not significant. A lateralized and distalized design (group 3) achieved superior results for flexion and abduction compared to the Grammont design and the lateralized design in a cohort of 226 patients with CTA. Lateralized implants (either with additional distalization or not) showed better rotational movement compared to the medialized and distalized Grammont design. To precise, the baseline/gender-adjusted CS showed a slight difference of 7 points (*p* = 0.03) between group 3 and group 1 at final 2-years follow-up. The clinical meaning of this fact is questionable as the cut-off number of the MCID (minimal clinically important difference) after RSA treatment for CTA in the literature is 8 points [25].

Pre- and postoperative radiographic measurements showed no relevant design-independent differences between the groups regarding scapular neck anatomy and implant positioning (GSIA was statistically significant but small angular differences of 4.1° (group 1 vs. 2) and 3.2° (group 1 vs 3) do not have clinical meaning).

In a similar study focused on Hamada Grade 1 to 3 cuff-deficient shoulders better external rotation and a trend towards better internal rotation with less scapular notching for lateralized (135° NSA and 4 mm lateralized glenosphere) over non-lateralized RSAs (155° NSA and 2 mm eccentric glenosphere) was reported [26]. The use of curved stem 145° NSA onlay designs introduced another type of RSA configuration; a computational ROM study for different humeral and glenosphere design concepts showed adequate restoration of glenohumeral ROM only for a lateralized NSA (145°) in combination with eccentric, large or lateralized spheres [14].

The LHO of group 3 (mean, 44.0 mm) was slightly higher than that of group 2 (mean, 40.9 mm). Based on the NSA (10° less distalization) and bigger glenoidal lateralization (additional 1 mm) in group 135°, this difference is arguably due to the onlay and curved stem design of group 145°. This is supported by the findings of Werthel et al. who found that twice the amount of lateralization can be achieved on the humeral side due to changes in design (i.e., onlay or curved stems) [12].

Glenoid lateralization is an accepted approach to decrease scapular notching [27–30] and increases impingement-free motion [31, 32]. In our group 2 the inferior glenosphere overhang was significantly lower than in 3, where an inferiorly eccentric glenodphere was used. This explains a higher value of scapular notching of group 2 in comparison to group 3. However, although eccentricity of the glenosphere was also used in group 1, values of mild scapular notching (grade 1) were significantly higher than in group 2 and 3, where bipolar (glenoidal and humeral) lateralization was performed. Comparing short-term results of a Grammont-style RSA versus the same 145° curved stem used in our study (a subgroup of those additionally treated with a BIO-RSA) showed less scapular notching with humeral lateralization [33].

With the center of rotation shifting more laterally with glenoidal lateralization shearing forces in elevation and abduction increase [34]. Consequently, acromial stress [35, 36] and shearing forces onto the glenoid also increase, which could potentially lead to spine stress fractures [37] or glenoid loosening in the long term [38]. We did observe 2 acromial fractures (Levy Typ 2) in group 2 and no acromial or scapular spine stress fractures in group 3. Overall, there was a low incidence of acromial fractures, with similar values reported in the literature [39] and there was no difference between the groups.

The position of the greater and lesser tuberosity becomes lateralized with a humeral lateralization design, which improves tensioning of the remaining cuff [40] that in turn, improves stability [41] as well as the lever arm [34, 42, 43] and deltoid wrapping [44]. Regarding glenoidal lateralization Collin et al. found that patients with a bony increased-offset RSA (BIO-RSA) achieved better functional results without any difference in ROM compared to those with a non-lateralized Grammont arthroplasty [45]. On the other hand, similar studies with small patient cohorts did not report any significant differences in functional outcomes of ROM, abduction strength, pain, or any other patient-reported scores in short-term follow-up (up to 2 years) [30, 46]. When humeral lateralization was introduced, higher functional outcome with glenoid lateralization and a BIO-RSA (CS: 70–71 points) [40, 47] or metallic baseplate offset (CS: 79 points) [48] was achieved.

The same 135° design as that used in our study showed better external rotation and greater abduction strength compared to a 155° design with a tGLO of 18.5 mm at the 1-year follow-up examination [49].

A comparative investigation of two matched cohorts with 135° NSA stems and an inlay (tGLO 23.5 mm) versus lateralized onlay (tGLO 29.3 mm) revealed no differences in scapular notching or acromial fractures, but better external rotation and forward flexion for the onlay design after 2 years [50]. Moreover, a 145° onlay design displayed better external rotation over a 155° inlay implant [51].

A short-term retrospective comparison of the extreme lateralizing Arrow prosthesis (tGLO 34.5 mm) versus the Grammont-style Delta III (tGLO 13.1 mm) showed less scapular notching and a trend towards better external rotation for the lateralized implant, yet without an overall superior clinical outcome [52].

These studies support our findings that the sum of bipolar lateralization (more rotational movement (humeral lateralization [49]) and less notching (glenoidal lateralization) [30, 52]) and distalization ( more flexion [50]) with inferior glenosphere overhang ( less notching [53]) provides best ROM despite no clinically significant difference in outcome scores could be found [30, 46, 52, 54].

All patients in our study had intact teres minor and there was equal distribution of complete infraspinatus ruptures between the groups. Considering the comparable percentage of subscapularis tears, the medialized design of group 1 limits internal rotation, whereas group 2 representing the most “anatomical” design showed similar results for internal rotation as group 3. In this context it is important to mention that all passive movement parameter assessed (flexion, abduction, external rotation) showed significantly higher values for group 3. A part of presumably better muscle tension, a higher passive impingement-free range of motion is likely to cause a better active movement too. The working groups of Streit and Lädermann reported better flexion for a design with greater distalization [14, 55]. We too found best values of flexion in group 3 with a significantly higher DSA compared to our other study groups. The combination of humeral lateralization and distalization of group 3 resulted in favorable abduction, whereas group 2 showed worst abduction values. This might be due to cases of subacromial impingement. Moreover, lateralization increases the force required for abduction due to delta wrapping around the lateralized implant. Additionally, less delta muscle is recruited for abduction in a mainly glenoid-side lateralized implant as the one used in group 2.

Every surgeon had his preferred implant design and there was no choice of implant based on patient’s characteristics, pathology or anatomy. Therefor the choice of implant was preset and there was no selection bias a far as the surgeon’s choice of implant is concerned.

The strengths of this study are the homogeneous distribution of the three patient cohorts each with the same implant configuration and diagnosis as well as the strict monitoring and continuous follow-up examination protocol. Moreover, a small number of experienced shoulder surgeons performed the RSAs in specialized shoulder arthroplasty centers. Nonetheless, we need to highlight limitations including the retrospective bicentric, observational study design and short follow-up. The heterogeneity of glenoid configurations and deformities as well as scapular setting and motion must also be considered. We didn’t adjust for patient comorbidities. Radiological measurements were all performed by one experienced investigator and thus, we cannot provide any estimations of inter-rater reliability. Finally, clinical evaluation of range of motion at follow-up postoperatively was assessed by different observers and differences on the clinical judgement of range of motion between observers cannot be excluded.

## Conclusion

There was no difference in outcome scores between a medialized and distalized, a lateralized and a lateralized and distalized RSA. The lateralized and distalized RSA implant was associated with better flexion and abduction. Furthermore, glenoid lateralization combined with an NSA lower than that of the original Grammont design was associated with a reduction of scapular notching. There was an association of further reduction of scapular notching with glenosphere eccentricity because of higher inferior glenosphere overhang. A better rotation was associated with both lateralized implant designs. The outlined design advantages should be favored over the Grammont design.

### Supplementary Information

**Supplementary Material 1.**

**Supplementary Material 2.**

**Supplementary Material 3.**

## Data Availability

The datasets used and/or analyzed during the current study are available from the corresponding author on reasonable request.
