# Supplementary material for: Analysis of three different reverse shoulder arthroplasty designs for cuff tear arthropathy – the combination of lateralization and distalization provides best mobility
Source: BMC Musculoskelet Disord. 2024 Mar 7;25:204. doi: 10.1186/s12891-024-07312-5 (PMC10918945; doi:10.1186/s12891-024-07312-5)
Supplement: Supplementary file 1 — Supplementary Material 1. [file 12891_2024_7312_MOESM1_ESM.docx]

**Supplementary file 1**

**Article title** Reverse shoulder arthroplasty for cuff tear arthropathy – clinical and radiological results of different lateralization concepts in comparison to the classic Grammont design

**Journal name** BMC Musculoskeletal Disorders

**Author names** Florian Freislederer, Philipp Moroder, Laurent Audige, Tim Schneller, Yacine Ameziane, Raphael Trefzer, Jan-Philipp Imiolczyk, Markus Scheibel

**Affiliation** Schulthess Klinik, CH-8008 Zurich, Switzerland

**E-mail address** florian.freislederer@kws.ch

**Baseline and postoperative shoulder range of motion (ROM) parameters, strength and functional scores**

|  | **155** |  | **145** |  | **135** |  | Adjusted | Model |
| --- | --- | --- | --- | --- | --- | --- | --- | --- |
| Active motion parameters | n | mean (SD) | n | mean (SD) | n | mean (SD) | p-value | p-value* |
| Flexion (°) |  |  |  |  |  |  |  | <0.001 |
| Baseline | 50 | 71 (33) | 35 | 69 (36) | 141 | 78 (39) |  |  |
| 6 months | 47 | 135 (20) | 14 | 136 (21) | 130 | 125 (25) | 0.108 |  |
| 12 months | 43 | 141 (18) | 22 | 146 (20) | 123 | 130 (23) | 0.002 |  |
| 24 months | 40 | 142 (18) | 33 | 155 (13) | 107 | 132 (18) | <0.001 |  |

SD = standard deviation;
* Mixed model p-value for group effect adjusted for age, gender and baseline pre-operative values

**Stata output**

**Overall mixed model**

Mixed-effects ML regression Number of obs = 559

Group variable: regid2 Number of groups = 218

Obs per group:

min = 1

avg = 2.6

max = 3

Wald chi2(7) = 109.90

Log likelihood = -2366.6702 Prob > chi2 = 0.0000

-------------------------------------------------------------------------------

rm_ante_bakt | Coefficient Std. err. z P>|z| [95% conf. interval]

--------------+----------------------------------------------------------------

rm_ante_bakt0 | .1085491 .0318931 3.40 0.001 .0460398 .1710583

_Ievent_12 | 5.450351 1.277082 4.27 0.000 2.947316 7.953386

_Ievent_24 | 8.561073 1.310968 6.53 0.000 5.991624 11.13052

Age | -.7041606 .1884538 -3.74 0.000 -1.073523 -.3347979

gender | 3.600579 3.759841 0.96 0.338 -3.768573 10.96973

_Igroup_2 | 9.796491 4.021427 2.44 0.015 1.91464 17.67834

_Igroup_3 | -7.715921 3.142713 -2.46 0.014 -13.87553 -1.556316

_cons | 177.6231 14.75556 12.04 0.000 148.7027 206.5435

-------------------------------------------------------------------------------

( 1) [rm_ante_bakt]_Igroup_2 = 0

( 2) [rm_ante_bakt]_Igroup_3 = 0

chi2( 2) = 23.72

Prob > chi2 = 0.0000

**Adjusted model at 6 months**

**-------------------------------------------------------------------------------**

**rm_ante_bakt | Coefficient Std. err. t P>|t| [95% conf. interval]**

**--------------+----------------------------------------------------------------**

**rm_ante_bakt0 | .1110963 .0445729 2.49 0.014 .0231597 .1990329**

**_Igroup_2 | 2.758742 6.88152 0.40 0.689 -10.8176 16.33508**

**_Igroup_3 | -7.606547 4.163383 -1.83 0.069 -15.82036 .6072658**

**Age | -1.058141 .2546117 -4.16 0.000 -1.560457 -.5558253**

**gender | 5.139844 5.345719 0.96 0.338 -5.406565 15.68625**

**_cons | 203.7838 20.03902 10.17 0.000 164.2495 243.3182**

**-------------------------------------------------------------------------------**

**( 1) _Igroup_2 = 0**

**( 2) _Igroup_3 = 0**

**F( 2, 185) = 2.25**

**Prob > F = 0.1085**

**Adjusted model at 12 months**

**-------------------------------------------------------------------------------**

**rm_ante_bakt | Coefficient Std. err. t P>|t| [95% conf. interval]**

**--------------+----------------------------------------------------------------**

**rm_ante_bakt0 | .1165398 .0407428 2.86 0.005 .0361508 .1969287**

**_Igroup_2 | 5.464909 5.558325 0.98 0.327 -5.502133 16.43195**

**_Igroup_3 | -10.3066 4.081022 -2.53 0.012 -18.3588 -2.254405**

**Age | -.7461942 .2438464 -3.06 0.003 -1.227324 -.2650646**

**gender | .3376701 5.027623 0.07 0.947 -9.582253 10.25759**

**_cons | 188.0713 18.95335 9.92 0.000 150.6748 225.4679**

**-------------------------------------------------------------------------------**

**( 1) _Igroup_2 = 0**

**( 2) _Igroup_3 = 0**

**F( 2, 182) = 6.39**

**Prob > F = 0.0021**

**Adjusted model at 24 months**

**-------------------------------------------------------------------------------**

**rm_ante_bakt | Coefficient Std. err. t P>|t| [95% conf. interval]**

**--------------+----------------------------------------------------------------**

**rm_ante_bakt0 | .1110814 .0319622 3.48 0.001 .0479979 .1741648**

**_Igroup_2 | 14.96793 3.864474 3.87 0.000 7.340653 22.59521**

**_Igroup_3 | -7.510615 3.323148 -2.26 0.025 -14.06948 -.9517467**

**Age | -.4572013 .2000565 -2.29 0.023 -.852051 -.0623515**

**gender | 4.129502 3.586243 1.15 0.251 -2.948636 11.20764**

**_cons | 165.4671 15.38818 10.75 0.000 135.0956 195.8386**

**-------------------------------------------------------------------------------**

**( 1) _Igroup_2 = 0**

**( 2) _Igroup_3 = 0**

**F( 2, 174) = 21.53**

**Prob > F = 0.0000**

**Baseline and postoperative shoulder range of motion (ROM) parameters, strength and functional scores**

|  | **155** |  | **145** |  | **135** |  | Adjusted | Model |
| --- | --- | --- | --- | --- | --- | --- | --- | --- |
| Active motion parameters | n | mean (SD) | n | mean (SD) | n | mean (SD) | p-value | p-value* |
| Abduction (°) |  |  |  |  |  |  |  | <0.001 |
| Baseline | 50 | 65 (25) | 35 | 62 (31) | 141 | 70 (34) |  |  |
| 6 months | 47 | 123 (23) | 14 | 120 (31) | 129 | 118 (28) | 0.949 |  |
| 12 months | 43 | 128 (22) | 22 | 138 (25) | 123 | 124 (26) | 0.061 |  |
| 24 months | 40 | 130 (22) | 33 | 147 (23) | 107 | 118 (25) | <0.001 |  |

SD = standard deviation;
* Mixed model p-value for group effect adjusted for age, gender and baseline pre-operative values

**Stata output**

**Overall mixed model**

Mixed-effects ML regression Number of obs = 558

Group variable: regid2 Number of groups = 218

Obs per group:

min = 1

avg = 2.6

max = 3

Wald chi2(7) = 74.15

Log likelihood = -2460.3934 Prob > chi2 = 0.0000

------------------------------------------------------------------------------

rm_abd_bakt | Coefficient Std. err. z P>|z| [95% conf. interval]

-------------+----------------------------------------------------------------

rm_abd_bakt0 | .1680793 .0461817 3.64 0.000 .0775648 .2585938

_Ievent_12 | 6.102832 1.508887 4.04 0.000 3.145469 9.060195

_Ievent_24 | 5.145215 1.549149 3.32 0.001 2.108938 8.181491

Age | -.8233527 .2280111 -3.61 0.000 -1.270246 -.3764592

gender | 9.145731 4.559 2.01 0.045 .2102557 18.08121

_Igroup_2 | 15.04638 4.873957 3.09 0.002 5.493596 24.59916

_Igroup_3 | -2.15883 3.807154 -0.57 0.571 -9.620715 5.303056

_cons | 169.6213 17.82373 9.52 0.000 134.6874 204.5552

------------------------------------------------------------------------------

( 1) [rm_abd_bakt]_Igroup_2 = 0

( 2) [rm_abd_bakt]_Igroup_3 = 0

chi2( 2) = 15.36

Prob > chi2 = 0.0005

**Adjusted model at 6 months**

**------------------------------------------------------------------------------**

**rm_abd_bakt | Coefficient Std. err. t P>|t| [95% conf. interval]**

**-------------+----------------------------------------------------------------**

**rm_abd_bakt0 | .2325297 .0607026 3.83 0.000 .1127671 .3522923**

**_Igroup_2 | .549157 7.785273 0.07 0.944 -14.81072 15.90904**

**_Igroup_3 | -1.282423 4.699299 -0.27 0.785 -10.55386 7.989015**

**Age | -.9725979 .2873883 -3.38 0.001 -1.539598 -.4055978**

**gender | 9.877417 6.035249 1.64 0.103 -2.029771 21.78461**

**_cons | 176.4378 22.62685 7.80 0.000 131.7964 221.0792**

**------------------------------------------------------------------------------**

**( 1) _Igroup_2 = 0**

**( 2) _Igroup_3 = 0**

**F( 2, 184) = 0.05**

**Prob > F = 0.9491**

**Adjusted model at 12 months**

**------------------------------------------------------------------------------**

**rm_abd_bakt | Coefficient Std. err. t P>|t| [95% conf. interval]**

**-------------+----------------------------------------------------------------**

**rm_abd_bakt0 | .1687974 .0555152 3.04 0.003 .0592613 .2783335**

**_Igroup_2 | 12.87128 6.286475 2.05 0.042 .467541 25.27503**

**_Igroup_3 | -.3335056 4.61008 -0.07 0.942 -9.42958 8.762569**

**Age | -.7835244 .2754367 -2.84 0.005 -1.326984 -.2400647**

**gender | 10.57251 5.675389 1.86 0.064 -.6255054 21.77053**

**_cons | 171.0733 21.42317 7.99 0.000 128.8036 213.343**

**------------------------------------------------------------------------------**

**( 1) _Igroup_2 = 0**

**( 2) _Igroup_3 = 0**

**F( 2, 182) = 2.83**

**Prob > F = 0.0613**

**Adjusted model at 24 months**

**------------------------------------------------------------------------------**

**rm_abd_bakt | Coefficient Std. err. t P>|t| [95% conf. interval]**

**-------------+----------------------------------------------------------------**

**rm_abd_bakt0 | .1670304 .0539068 3.10 0.002 .0606351 .2734258**

**_Igroup_2 | 18.70029 5.448729 3.43 0.001 7.946176 29.4544**

**_Igroup_3 | -8.43699 4.682397 -1.80 0.073 -17.6786 .8046166**

**Age | -.7130125 .2815986 -2.53 0.012 -1.268801 -.1572238**

**gender | 8.050142 5.056749 1.59 0.113 -1.930321 18.0306**

**_cons | 168.6094 21.68054 7.78 0.000 125.8187 211.4001**

**------------------------------------------------------------------------------**

**( 1) _Igroup_2 = 0**

**( 2) _Igroup_3 = 0**

**F( 2, 174) = 15.77**

**Prob > F = 0.0000**

**Baseline and postoperative shoulder range of motion (ROM) parameters, strength and functional scores**

|  | **155** |  | **145** |  | **135** |  | Adjusted | Model |
| --- | --- | --- | --- | --- | --- | --- | --- | --- |
| Active motion parameters | n | mean (SD) | n | mean (SD) | n | mean (SD) | p-value | p-value* |
| External rotation in 0° abd. (°) |  |  |  |  |  |  |  | <0.001 |
| Baseline | 48 | 30 (18) | 35 | 22 (23) | 141 | 31 (22) |  |  |
| 6 months | 47 | 22 (11) | 14 | 41 (25) | 131 | 33 (14) | <0.001 |  |
| 12 months | 43 | 23 (11) | 22 | 38 (21) | 123 | 35 (15) | <0.001 |  |
| 24 months | 40 | 25 (16) | 33 | 41 (23) | 107 | 38 (17) | <0.001 |  |

SD = standard deviation;
* Mixed model p-value for group effect adjusted for age, gender and baseline pre-operative values

**Stata output**

**Overall mixed model**

Mixed-effects ML regression Number of obs = 555

Group variable: regid2 Number of groups = 216

Obs per group:

min = 1

avg = 2.6

max = 3

Wald chi2(7) = 114.79

Log likelihood = -2229.8773 Prob > chi2 = 0.0000

------------------------------------------------------------------------------

rm_ar0_bakt | Coefficient Std. err. z P>|z| [95% conf. interval]

-------------+----------------------------------------------------------------

rm_ar0_bakt0 | .278643 .0385469 7.23 0.000 .2030924 .3541936

_Ievent_12 | 1.905239 1.150947 1.66 0.098 -.3505756 4.161054

_Ievent_24 | 4.136048 1.178618 3.51 0.000 1.825998 6.446097

Age | -.0951701 .128548 -0.74 0.459 -.3471195 .1567794

gender | -1.334111 2.538991 -0.53 0.599 -6.310441 3.642219

_Igroup_2 | 17.7643 2.767093 6.42 0.000 12.3409 23.1877

_Igroup_3 | 11.36723 2.15156 5.28 0.000 7.150251 15.58421

_cons | 20.243 10.06753 2.01 0.044 .5109986 39.97499

------------------------------------------------------------------------------

( 1) [rm_ar0_bakt]_Igroup_2 = 0

( 2) [rm_ar0_bakt]_Igroup_3 = 0

chi2( 2) = 47.12

Prob > chi2 = 0.0000

**Adjusted model at 6 months**

**------------------------------------------------------------------------------**

**rm_ar0_bakt | Coefficient Std. err. t P>|t| [95% conf. interval]**

**-------------+----------------------------------------------------------------**

**rm_ar0_bakt0 | .2862783 .0462346 6.19 0.000 .1950601 .3774965**

**_Igroup_2 | 18.98683 4.030384 4.71 0.000 11.03512 26.93853**

**_Igroup_3 | 10.91246 2.492062 4.38 0.000 5.995764 15.82915**

**Age | -.0583115 .1515128 -0.38 0.701 -.3572372 .2406142**

**gender | .0538325 3.159913 0.02 0.986 -6.180489 6.288154**

**_cons | 17.26409 11.9325 1.45 0.150 -6.278027 40.80621**

**------------------------------------------------------------------------------**

**( 1) _Igroup_2 = 0**

**( 2) _Igroup_3 = 0**

**F( 2, 184) = 15.24**

**Prob > F = 0.0000**

**Adjusted model at 12 months**

**------------------------------------------------------------------------------**

**rm_ar0_bakt | Coefficient Std. err. t P>|t| [95% conf. interval]**

**-------------+----------------------------------------------------------------**

**rm_ar0_bakt0 | .2726523 .0483301 5.64 0.000 .1772859 .3680188**

**_Igroup_2 | 18.8096 3.662322 5.14 0.000 11.58299 26.03621**

**_Igroup_3 | 10.67451 2.683017 3.98 0.000 5.380302 15.96872**

**Age | -.1007281 .1594937 -0.63 0.528 -.4154461 .2139898**

**gender | -3.722428 3.253239 -1.14 0.254 -10.14182 2.696964**

**_cons | 23.26572 12.4678 1.87 0.064 -1.336126 47.86756**

**------------------------------------------------------------------------------**

**( 1) _Igroup_2 = 0**

**( 2) _Igroup_3 = 0**

**F( 2, 180) = 14.61**

**Prob > F = 0.0000**

**Adjusted model at 24 months**

**------------------------------------------------------------------------------**

**rm_ar0_bakt | Coefficient Std. err. t P>|t| [95% conf. interval]**

**-------------+----------------------------------------------------------------**

**rm_ar0_bakt0 | .2952279 .0608857 4.85 0.000 .1750534 .4154024**

**_Igroup_2 | 18.39239 4.01806 4.58 0.000 10.46166 26.32313**

**_Igroup_3 | 11.40344 3.443944 3.31 0.001 4.605882 18.201**

**Age | -.2569789 .206787 -1.24 0.216 -.6651291 .1511712**

**gender | -1.860622 3.696494 -0.50 0.615 -9.156655 5.435411**

**_cons | 35.88989 15.89793 2.26 0.025 4.51102 67.26877**

**------------------------------------------------------------------------------**

**( 1) _Igroup_2 = 0**

**( 2) _Igroup_3 = 0**

**F( 2, 173) = 10.94**

**Prob > F = 0.0000**

**Baseline and postoperative shoulder range of motion (ROM) parameters, strength and functional scores**

|  | **155** |  | **145** |  | **135** |  | Adjusted | Model |
| --- | --- | --- | --- | --- | --- | --- | --- | --- |
| Passive motion parameters | n | mean (SD) | n | mean (SD) | n | mean (SD) | p-value | p-value* |
| Flexion passive (°) |  |  |  |  |  |  |  |  |
| Baseline | 50 | 88 (39) | 35 | 94 (45) | 141 | 97 (39) |  | <0.001 |
| 6 months | 47 | 142 (19) | 14 | 146 (18) | 130 | 128 (22) | 0.001 |  |
| 12 months | 43 | 149 (18) | 22 | 157 (15) | 123 | 133 (20) | <0.001 |  |
| 24 months | 40 | 147 (18) | 33 | 163 (11) | 107 | 135 (17) | <0.001 |  |

SD = standard deviation;
* Mixed model p-value for group effect adjusted for age, gender and baseline pre-operative values

**Stata output**

**Overall mixed model**

Mixed-effects ML regression Number of obs = 559

Group variable: regid2 Number of groups = 218

Obs per group:

min = 1

avg = 2.6

max = 3

Wald chi2(7) = 159.35

Log likelihood = -2308.9043 Prob > chi2 = 0.0000

-------------------------------------------------------------------------------

rm_ante_bpas | Coefficient Std. err. z P>|z| [95% conf. interval]

--------------+----------------------------------------------------------------

rm_ante_bpas0 | .0977128 .0266079 3.67 0.000 .0455623 .1498633

_Ievent_12 | 5.169807 1.161823 4.45 0.000 2.892675 7.446939

_Ievent_24 | 7.325585 1.192575 6.14 0.000 4.988182 9.662988

Age | -.6255816 .1673231 -3.74 0.000 -.9535288 -.2976343

gender | 2.067443 3.35382 0.62 0.538 -4.505924 8.640809

_Igroup_2 | 11.5356 3.57943 3.22 0.001 4.520049 18.55116

_Igroup_3 | -13.10599 2.807141 -4.67 0.000 -18.60788 -7.604095

_cons | 178.9299 13.10838 13.65 0.000 153.238 204.6219

-------------------------------------------------------------------------------

( 1) [rm_ante_bpas]_Igroup_2 = 0

( 2) [rm_ante_bpas]_Igroup_3 = 0

chi2( 2) = 62.93

Prob > chi2 = 0.0000

**Adjusted model at 6 months**

**-------------------------------------------------------------------------------**

**rm_ante_bpas | Coefficient Std. err. t P>|t| [95% conf. interval]**

**--------------+----------------------------------------------------------------**

**rm_ante_bpas0 | .1073322 .0371225 2.89 0.004 .0340943 .1805702**

**_Igroup_2 | 3.63547 6.066304 0.60 0.550 -8.332559 15.6035**

**_Igroup_3 | -12.57467 3.718997 -3.38 0.001 -19.91177 -5.237574**

**Age | -.9215986 .2252333 -4.09 0.000 -1.365955 -.4772427**

**gender | 5.949378 4.774311 1.25 0.214 -3.469716 15.36847**

**_cons | 199.3883 17.62288 11.31 0.000 164.6206 234.1559**

**-------------------------------------------------------------------------------**

**( 1) _Igroup_2 = 0**

**( 2) _Igroup_3 = 0**

**F( 2, 185) = 7.45**

**Prob > F = 0.0008**

**Adjusted model at 12 months**

**-------------------------------------------------------------------------------**

**rm_ante_bpas | Coefficient Std. err. t P>|t| [95% conf. interval]**

**--------------+----------------------------------------------------------------**

**rm_ante_bpas0 | .1112948 .0334201 3.33 0.001 .045354 .1772355**

**_Igroup_2 | 8.051393 4.723679 1.70 0.090 -1.268823 17.37161**

**_Igroup_3 | -16.1797 3.493405 -4.63 0.000 -23.07249 -9.286923**

**Age | -.7088224 .2066832 -3.43 0.001 -1.116626 -.301019**

**gender | -.6946682 4.284923 -0.16 0.871 -9.149182 7.759846**

**_cons | 191.7146 16.08249 11.92 0.000 159.9825 223.4467**

**-------------------------------------------------------------------------------**

**( 1) _Igroup_2 = 0**

**( 2) _Igroup_3 = 0**

**F( 2, 182) = 21.13**

**Prob > F = 0.0000**

**Adjusted model at 24 months**

**-------------------------------------------------------------------------------**

**rm_ante_bpas | Coefficient Std. err. t P>|t| [95% conf. interval]**

**--------------+----------------------------------------------------------------**

**rm_ante_bpas0 | .1001072 .0284216 3.52 0.001 .0440118 .1562027**

**_Igroup_2 | 15.63722 3.713152 4.21 0.000 8.308605 22.96584**

**_Igroup_3 | -12.24568 3.200146 -3.83 0.000 -18.56178 -5.929578**

**Age | -.4355175 .1915245 -2.27 0.024 -.8135277 -.0575073**

**gender | .8728941 3.451249 0.25 0.801 -5.938806 7.684594**

**_cons | 169.9543 14.73097 11.54 0.000 140.8799 199.0287**

**-------------------------------------------------------------------------------**

**( 1) _Igroup_2 = 0**

**( 2) _Igroup_3 = 0**

**F( 2, 174) = 36.58**

**Prob > F = 0.0000**

**Baseline and postoperative shoulder range of motion (ROM) parameters, strength and functional scores**

|  | **155** |  | **145** |  | **135** |  | Adjusted | Model |
| --- | --- | --- | --- | --- | --- | --- | --- | --- |
| Passive motion parameters | n | mean (SD) | n | mean (SD) | n | mean (SD) | p-value | p-value* |
| Abduction passive (°) |  |  |  |  |  |  |  |  |
| Baseline | 50 | 77 (34) | 35 | 90 (44) | 141 | 85 (38) |  | <0.001 |
| 6 months | 47 | 131 (21) | 14 | 130 (32) | 129 | 121 (25) | 0.237 |  |
| 12 months | 43 | 135 (21) | 22 | 151 (20) | 123 | 125 (24) | <0.001 |  |
| 24 months | 40 | 136 (21) | 33 | 156 (21) | 107 | 121 (24) | <0.001 |  |

SD = standard deviation;
* Mixed model p-value for group effect adjusted for age, gender and baseline pre-operative values

**Stata output**

**Overall mixed model**

Mixed-effects ML regression Number of obs = 558

Group variable: regid2 Number of groups = 218

Obs per group:

min = 1

avg = 2.6

max = 3

Wald chi2(7) = 113.48

Log likelihood = -2411.3734 Prob > chi2 = 0.0000

------------------------------------------------------------------------------

rm_abd_bpas | Coefficient Std. err. z P>|z| [95% conf. interval]

-------------+----------------------------------------------------------------

rm_abd_bpas0 | .1625592 .0346938 4.69 0.000 .0945606 .2305578

_Ievent_12 | 5.198128 1.395092 3.73 0.000 2.463798 7.932458

_Ievent_24 | 3.709309 1.432242 2.59 0.010 .9021655 6.516452

Age | -.643801 .205697 -3.13 0.002 -1.04696 -.2406422

gender | 7.603147 4.117913 1.85 0.065 -.467814 15.67411

_Igroup_2 | 16.44297 4.410729 3.73 0.000 7.798096 25.08784

_Igroup_3 | -8.267949 3.445804 -2.40 0.016 -15.0216 -1.514297

_cons | 163.2396 16.06758 10.16 0.000 131.7477 194.7315

------------------------------------------------------------------------------

( 1) [rm_abd_bpas]_Igroup_2 = 0

( 2) [rm_abd_bpas]_Igroup_3 = 0

chi2( 2) = 38.35

Prob > chi2 = 0.0000

**Adjusted model at 6 months**

**------------------------------------------------------------------------------**

**rm_abd_bpas | Coefficient Std. err. t P>|t| [95% conf. interval]**

**-------------+----------------------------------------------------------------**

**rm_abd_bpas0 | .2307018 .0444335 5.19 0.000 .1430373 .3183664**

**_Igroup_2 | -2.019208 6.816687 -0.30 0.767 -15.46813 11.42971**

**_Igroup_3 | -6.991319 4.169101 -1.68 0.095 -15.21671 1.234069**

**Age | -.7218151 .2533815 -2.85 0.005 -1.221722 -.2219085**

**gender | 12.62508 5.342482 2.36 0.019 2.084683 23.16548**

**_cons | 162.6898 19.78756 8.22 0.000 123.6501 201.7295**

**------------------------------------------------------------------------------**

**( 1) _Igroup_2 = 0**

**( 2) _Igroup_3 = 0**

**F( 2, 184) = 1.45**

**Prob > F = 0.2367**

**Adjusted model at 12 months**

**------------------------------------------------------------------------------**

**rm_abd_bpas | Coefficient Std. err. t P>|t| [95% conf. interval]**

**-------------+----------------------------------------------------------------**

**rm_abd_bpas0 | .1656531 .0423774 3.91 0.000 .082039 .2492672**

**_Igroup_2 | 14.81119 5.612646 2.64 0.009 3.736971 25.88542**

**_Igroup_3 | -8.010487 4.131078 -1.94 0.054 -16.16145 .1404775**

**Age | -.7073146 .24452 -2.89 0.004 -1.189773 -.2248561**

**gender | 7.072815 5.060909 1.40 0.164 -2.912785 17.05841**

**_cons | 172.6274 19.013 9.08 0.000 135.1131 210.1416**

**------------------------------------------------------------------------------**

**( 1) _Igroup_2 = 0**

**( 2) _Igroup_3 = 0**

**F( 2, 182) = 10.49**

**Prob > F = 0.0000**

**Adjusted model at 24 months**

**------------------------------------------------------------------------------**

**rm_abd_bpas | Coefficient Std. err. t P>|t| [95% conf. interval]**

**-------------+----------------------------------------------------------------**

**rm_abd_bpas0 | .1467642 .041775 3.51 0.001 .0643132 .2292152**

**_Igroup_2 | 20.00067 5.157227 3.88 0.000 9.821892 30.17944**

**_Igroup_3 | -12.90524 4.424897 -2.92 0.004 -21.63862 -4.171859**

**Age | -.5668383 .26554 -2.13 0.034 -1.090932 -.0427443**

**gender | 5.349543 4.770788 1.12 0.264 -4.066521 14.76561**

**_cons | 163.9185 20.43861 8.02 0.000 123.579 204.258**

**------------------------------------------------------------------------------**

**( 1) _Igroup_2 = 0**

**( 2) _Igroup_3 = 0**

**F( 2, 174) = 26.42**

**Prob > F = 0.0000**

**Baseline and postoperative shoulder range of motion (ROM) parameters, strength and functional scores**

|  | **155** |  | **145** |  | **135** |  | Adjusted | Model |
| --- | --- | --- | --- | --- | --- | --- | --- | --- |
| Passive motion parameters | n | mean (SD) | n | mean (SD) | n | mean (SD) | p-value | p-value* |
| External rotation in 0° abd. passive (°) |  |  |  |  |  |  |  |  |
| Baseline | 46 | 35 (18) | 35 | 31 (24) | 139 | 38 (23) |  | <0.001 |
| 6 months | 47 | 30 (9) | 14 | 50 (22) | 131 | 34 (13) | <0.001 |  |
| 12 months | 43 | 31 (12) | 22 | 46 (18) | 122 | 36 (14) | <0.001 |  |
| 24 months | 40 | 35 (14) | 33 | 50 (23) | 107 | 43 (14) | <0.001 |  |

SD = standard deviation;
* Mixed model p-value for group effect adjusted for age, gender and baseline pre-operative values

**Stata output**

**Overall mixed model**

Mixed-effects ML regression Number of obs = 545

Group variable: regid2 Number of groups = 212

Obs per group:

min = 1

avg = 2.6

max = 3

Wald chi2(7) = 121.39

Log likelihood = -2181.1909 Prob > chi2 = 0.0000

------------------------------------------------------------------------------

rm_ar0_bpas | Coefficient Std. err. z P>|z| [95% conf. interval]

-------------+----------------------------------------------------------------

rm_ar0_bpas0 | .1978143 .0346242 5.71 0.000 .1299521 .2656764

_Ievent_12 | 1.989067 1.189069 1.67 0.094 -.3414659 4.3196

_Ievent_24 | 7.730823 1.218157 6.35 0.000 5.343279 10.11837

Age | -.0307625 .1185683 -0.26 0.795 -.2631521 .2016272

gender | .5065369 2.346972 0.22 0.829 -4.093443 5.106517

_Igroup_2 | 15.99241 2.567096 6.23 0.000 10.961 21.02383

_Igroup_3 | 5.616598 2.02697 2.77 0.006 1.64381 9.589385

_cons | 23.57831 9.277034 2.54 0.011 5.395654 41.76096

------------------------------------------------------------------------------

( 1) [rm_ar0_bpas]_Igroup_2 = 0

( 2) [rm_ar0_bpas]_Igroup_3 = 0

chi2( 2) = 39.01

Prob > chi2 = 0.0000

**Adjusted model at 6 months**

**------------------------------------------------------------------------------**

**rm_ar0_bpas | Coefficient Std. err. t P>|t| [95% conf. interval]**

**-------------+----------------------------------------------------------------**

**rm_ar0_bpas0 | .2063486 .0424613 4.86 0.000 .1225659 .2901314**

**_Igroup_2 | 19.01538 3.800415 5.00 0.000 11.51656 26.51419**

**_Igroup_3 | 4.489314 2.389032 1.88 0.062 -.2246226 9.20325**

**Age | -.1518892 .1414682 -1.07 0.284 -.4310283 .1272498**

**gender | 1.259015 2.974029 0.42 0.673 -4.609212 7.127243**

**_cons | 32.86528 11.12497 2.95 0.004 10.91397 54.81659**

**------------------------------------------------------------------------------**

**( 1) _Igroup_2 = 0**

**( 2) _Igroup_3 = 0**

**F( 2, 181) = 12.52**

**Prob > F = 0.0000**

**Adjusted model at 12 months**

**------------------------------------------------------------------------------**

**rm_ar0_bpas | Coefficient Std. err. t P>|t| [95% conf. interval]**

**-------------+----------------------------------------------------------------**

**rm_ar0_bpas0 | .154516 .0471604 3.28 0.001 .061447 .2475849**

**_Igroup_2 | 17.02563 3.654884 4.66 0.000 9.812872 24.23839**

**_Igroup_3 | 4.968897 2.735369 1.82 0.071 -.4292367 10.36703**

**Age | -.0324306 .1614786 -0.20 0.841 -.3511018 .2862405**

**gender | -1.543447 3.270183 -0.47 0.638 -7.997013 4.910118**

**_cons | 27.66044 12.61318 2.19 0.030 2.768879 52.55201**

**------------------------------------------------------------------------------**

**( 1) _Igroup_2 = 0**

**( 2) _Igroup_3 = 0**

**F( 2, 177) = 11.02**

**Prob > F = 0.0000**

**Adjusted model at 24 months**

**------------------------------------------------------------------------------**

**rm_ar0_bpas | Coefficient Std. err. t P>|t| [95% conf. interval]**

**-------------+----------------------------------------------------------------**

**rm_ar0_bpas0 | .2149696 .0562413 3.82 0.000 .1039437 .3259955**

**_Igroup_2 | 15.96138 3.848045 4.15 0.000 8.364955 23.55781**

**_Igroup_3 | 7.39591 3.372333 2.19 0.030 .7385855 14.05323**

**Age | -.0505802 .1973284 -0.26 0.798 -.4401264 .3389659**

**gender | .3591307 3.538397 0.10 0.919 -6.62602 7.344282**

**_cons | 31.01702 15.13476 2.05 0.042 1.139488 60.89456**

**------------------------------------------------------------------------------**

**( 1) _Igroup_2 = 0**

**( 2) _Igroup_3 = 0**

**F( 2, 169) = 8.62**

**Prob > F = 0.0003**

**Baseline and postoperative shoulder range of motion (ROM) parameters, strength and functional scores**

|  | **155** |  | **145** |  | **135** |  | Adjusted | Model |
| --- | --- | --- | --- | --- | --- | --- | --- | --- |
| Strength, pain level and functional scores | n | mean (SD) | n | mean (SD) | n | mean (SD) | p-value | p-value* |
| Strength in abduction (kg) |  |  |  |  |  |  |  | 0.768 |
| Baseline | 50 | 0.4 (0.9) | 19 | 0.3 (1.2) | 141 | 0.4 (1.1) |  |  |
| 6 months | 45 | 4.2 (2.3) | 14 | 3.8 (2.3) | 130 | 3.3 (1.8) | 0.669 |  |
| 12 months | 42 | 4.7 (2.3) | 9 | 4.4 (2.3) | 120 | 3.9 (2.0) | 0.950 |  |
| 24 months | 39 | 5.3 (2.4) | 17 | 4.8 (2.2) | 106 | 3.9 (2.0) | 0.627 |  |

SD = standard deviation;
* Mixed model p-value for group effect adjusted for age, gender and baseline pre-operative values

**Stata output**

**Overall mixed model**

Mixed-effects ML regression Number of obs = 522

Group variable: regid2 Number of groups = 201

Obs per group:

min = 1

avg = 2.6

max = 3

Wald chi2(7) = 115.71

Log likelihood = -966.02774 Prob > chi2 = 0.0000

------------------------------------------------------------------------------

abdkraft_b | Coefficient Std. err. z P>|z| [95% conf. interval]

-------------+----------------------------------------------------------------

abdkraft_b0 | -.1039945 .1084815 -0.96 0.338 -.3166144 .1086254

_Ievent_12 | .6531907 .1199318 5.45 0.000 .4181286 .8882528

_Ievent_24 | .7004418 .1227945 5.70 0.000 .4597691 .9411145

Age | -.0910187 .0183268 -4.97 0.000 -.1269385 -.055099

gender | 2.038887 .3797056 5.37 0.000 1.294677 2.783096

_Igroup_2 | -.3308473 .4679043 -0.71 0.480 -1.247923 .5862283

_Igroup_3 | -.1266632 .2995775 -0.42 0.672 -.7138242 .4604978

_cons | 10.31667 1.401075 7.36 0.000 7.570614 13.06273

------------------------------------------------------------------------------

( 1) [abdkraft_b]_Igroup_2 = 0

( 2) [abdkraft_b]_Igroup_3 = 0

chi2( 2) = 0.53

Prob > chi2 = 0.7680

**Adjusted model at 6 months**

**------------------------------------------------------------------------------**

**abdkraft_b | Coefficient Std. err. t P>|t| [95% conf. interval]**

**-------------+----------------------------------------------------------------**

**abdkraft_b0 | -.125347 .1293137 -0.97 0.334 -.3804844 .1297905**

**_Igroup_2 | -.4984111 .5635124 -0.88 0.378 -1.610228 .6134057**

**_Igroup_3 | -.1594104 .3469724 -0.46 0.646 -.843991 .5251702**

**Age | -.0844848 .0209814 -4.03 0.000 -.1258814 -.0430883**

**gender | 1.729149 .4391528 3.94 0.000 .8626957 2.595603**

**_cons | 9.884965 1.609604 6.14 0.000 6.709197 13.06073**

**------------------------------------------------------------------------------**

**( 1) _Igroup_2 = 0**

**( 2) _Igroup_3 = 0**

**F( 2, 183) = 0.40**

**Prob > F = 0.6688**

**Adjusted model at 12 months**

**------------------------------------------------------------------------------**

**abdkraft_b | Coefficient Std. err. t P>|t| [95% conf. interval]**

**-------------+----------------------------------------------------------------**

**abdkraft_b0 | -.0463575 .1348875 -0.34 0.732 -.3126855 .2199705**

**_Igroup_2 | -.1682195 .7058901 -0.24 0.812 -1.561961 1.225522**

**_Igroup_3 | .042884 .3732893 0.11 0.909 -.6941555 .7799235**

**Age | -.0974938 .0231816 -4.21 0.000 -.1432645 -.0517231**

**gender | 2.116089 .5012313 4.22 0.000 1.126435 3.105743**

**_cons | 11.19448 1.77072 6.32 0.000 7.698286 14.69067**

**------------------------------------------------------------------------------**

**( 1) _Igroup_2 = 0**

**( 2) _Igroup_3 = 0**

**F( 2, 165) = 0.05**

**Prob > F = 0.9503**

**Adjusted model at 24 months**

**------------------------------------------------------------------------------**

**abdkraft_b | Coefficient Std. err. t P>|t| [95% conf. interval]**

**-------------+----------------------------------------------------------------**

**abdkraft_b0 | -.0816004 .1293085 -0.63 0.529 -.3370218 .173821**

**_Igroup_2 | -.4180818 .5452682 -0.77 0.444 -1.495143 .6589796**

**_Igroup_3 | -.3365326 .3912187 -0.86 0.391 -1.109302 .4362368**

**Age | -.0965456 .0240989 -4.01 0.000 -.1441477 -.0489434**

**gender | 2.358852 .4505413 5.24 0.000 1.468904 3.248801**

**_cons | 11.45017 1.819056 6.29 0.000 7.85701 15.04333**

**------------------------------------------------------------------------------**

**( 1) _Igroup_2 = 0**

**( 2) _Igroup_3 = 0**

**F( 2, 156) = 0.47**

**Prob > F = 0.6272**

**Baseline and postoperative shoulder range of motion (ROM) parameters, strength and functional scores**

|  | **155** |  | **145** |  | **135** |  | Adjusted | Model |
| --- | --- | --- | --- | --- | --- | --- | --- | --- |
| Pain NRS (0=no pain, 10=maximum pain) | n | mean (SD) | n | mean (SD) | n | mean (SD) | p-value | p-value* |
| Baseline | 46 | 6.1 (3.0) | 33 | 6.2 (2.7) | 129 | 6.4 (2.5) |  |  |
| 6 months | 49 | 1.4 (1.7) | 15 | 0.7 (0.9) | 129 | 1.6 (1.9) | 0.348 |  |
| 12 months | 44 | 1.4 (1.7) | 27 | 0.6 (1.2) | 129 | 1.3 (1.9) | 0.055 |  |
| 24 months | 47 | 1.7 (2.1) | 34 | 0.6 (1.2) | 128 | 1.5 (2.1) | 0.032 |  |

SD = standard deviation;
* Mixed model p-value for group effect adjusted for age, gender and baseline pre-operative values

**Stata output**

**Overall mixed model**

| Mixed-effects ML regression Number of obs = 564  Group variable: regid2 Number of groups = 204  Obs per group:  min = 1  avg = 2.8  max = 3  Wald chi2(7) = 23.93  Log likelihood = -1100.9414 Prob > chi2 = 0.0012  ------------------------------------------------------------------------------  cs_01 \| Coefficient Std. err. z P>\|z\| [95% conf. interval]  -------------+----------------------------------------------------------------  cs_010 \| .1381544 .0390856 3.53 0.000 .0615482 .2147607  _Ievent_12 \| -.1542042 .1489878 -1.04 0.301 -.4462149 .1378065  _Ievent_24 \| -.0081484 .1473048 -0.06 0.956 -.2968604 .2805637  Age \| -.0125973 .0159305 -0.79 0.429 -.0438205 .0186258  gender \| .2105951 .3196769 0.66 0.510 -.4159601 .8371503  _Igroup_2 \| -.8766809 .3427692 -2.56 0.011 -1.548496 -.2048656  _Igroup_3 \| .053557 .2647532 0.20 0.840 -.4653497 .5724638  _cons \| 1.591182 1.217861 1.31 0.191 -.7957829 3.978146  ------------------------------------------------------------------------------  ( 1) [cs_01]_Igroup_2 = 0  ( 2) [cs_01]_Igroup_3 = 0  chi2( 2) = 9.65  Prob > chi2 = 0.0080 | **Mixed-effects tobit regression Number of obs = 564**  **Uncensored = 301**  **Limits: Lower = 0 Left-censored = 263**  **Upper = 10 Right-censored = 0**  **Group variable: regid2 Number of groups = 204**  **Obs per group:**  **min = 1**  **avg = 2.8**  **max = 3**  **Integration method: mvaghermite Integration pts. = 7**  **Wald chi2(7) = 26.43**  **Log likelihood = -886.00518 Prob > chi2 = 0.0004**  **------------------------------------------------------------------------------**  **cs_01 \| Coefficient Std. err. z P>\|z\| [95% conf. interval]**  **-------------+----------------------------------------------------------------**  **cs_010 \| .2703189 .0771787 3.50 0.000 .1190513 .4215865**  **_Ievent_12 \| -.429119 .2542084 -1.69 0.091 -.9273582 .0691202**  **_Ievent_24 \| -.1784709 .2493632 -0.72 0.474 -.6672138 .3102721**  **Age \| -.034407 .0302174 -1.14 0.255 -.0936321 .024818**  **gender \| .0560501 .6210617 0.09 0.928 -1.161209 1.273309**  **_Igroup_2 \| -1.979458 .6850942 -2.89 0.004 -3.322218 -.6366981**  **_Igroup_3 \| -.0077228 .499888 -0.02 0.988 -.9874852 .9720396**  **_cons \| 1.561586 2.30495 0.68 0.498 -2.956033 6.079206**  **-------------+----------------------------------------------------------------**  **( 1) [cs_01]_Igroup_2 = 0**  **( 2) [cs_01]_Igroup_3 = 0**  **chi2( 2) = 10.97**  **Prob > chi2 = 0.0042** |
| --- | --- |

**Adjusted model at 6 months**

**------------------------------------------------------------------------------**

**cs_01 | Coefficient Std. err. t P>|t| [95% conf. interval]**

**-------------+----------------------------------------------------------------**

**cs_010 | .1067996 .052143 2.05 0.042 .0038895 .2097098**

**_Igroup_2 | -.7102656 .5430396 -1.31 0.193 -1.782015 .3614841**

**_Igroup_3 | .0242735 .3410031 0.07 0.943 -.6487345 .6972815**

**Age | -.0099744 .0209807 -0.48 0.635 -.0513821 .0314333**

**gender | -.2770209 .4327999 -0.64 0.523 -1.1312 .5771584**

**_cons | 1.689466 1.606987 1.05 0.295 -1.482103 4.861035**

**------------------------------------------------------------------------------**

**( 1) _Igroup_2 = 0**

**( 2) _Igroup_3 = 0**

**F( 2, 175) = 1.06**

**Prob > F = 0.3477**

**Adjusted model at 12 months**

**------------------------------------------------------------------------------**

**cs_01 | Coefficient Std. err. t P>|t| [95% conf. interval]**

**-------------+----------------------------------------------------------------**

**cs_010 | .1684743 .0499011 3.38 0.001 .0700116 .2669371**

**_Igroup_2 | -.8116391 .4425541 -1.83 0.068 -1.684868 .0615897**

**_Igroup_3 | .1437578 .340157 0.42 0.673 -.5274254 .8149409**

**Age | -.0101997 .0202435 -0.50 0.615 -.0501433 .029744**

**gender | .4935014 .4090403 1.21 0.229 -.3135993 1.300602**

**_cons | .9328907 1.537249 0.61 0.545 -2.100342 3.966124**

**------------------------------------------------------------------------------**

**( 1) _Igroup_2 = 0**

**( 2) _Igroup_3 = 0**

**F( 2, 181) = 2.95**

**Prob > F = 0.0550**

**Adjusted model at 24 months**

**------------------------------------------------------------------------------**

**cs_01 | Coefficient Std. err. t P>|t| [95% conf. interval]**

**-------------+----------------------------------------------------------------**

**cs_010 | .1450605 .0539684 2.69 0.008 .0386064 .2515146**

**_Igroup_2 | -.9387908 .459847 -2.04 0.043 -1.845852 -.0317295**

**_Igroup_3 | .1167805 .3725014 0.31 0.754 -.617989 .85155**

**Age | -.0217401 .022182 -0.98 0.328 -.0654947 .0220144**

**gender | .4820415 .4484494 1.07 0.284 -.4025375 1.36662**

**_cons | 2.145474 1.700291 1.26 0.209 -1.208399 5.499346**

**------------------------------------------------------------------------------**

**( 1) _Igroup_2 = 0**

**( 2) _Igroup_3 = 0**

**F( 2, 190) = 3.50**

**Prob > F = 0.0323**

**Baseline and postoperative shoulder range of motion (ROM) parameters, strength and functional scores**

|  | **155** |  | **145** |  | **135** |  | Adjusted | Model |
| --- | --- | --- | --- | --- | --- | --- | --- | --- |
| Strength, pain level and functional scores | n | mean (SD) | n | mean (SD) | n | mean (SD) | p-value | p-value* |
| CS Constant Murley Score (0-100=best) |  |  |  |  |  |  |  | 0.466 |
| Baseline | 42 | 27 (11) | 20 | 30 (14) | 122 | 31 (15) |  |  |
| 6 months | 40 | 65 (12) | 11 | 64 (10) | 114 | 62 (13) | 0.576 |  |
| 12 months | 38 | 69 (10) | 21 | 70 (10) | 105 | 67 (13) | 0.533 |  |
| 24 months | 33 | 68 (11) | 31 | 76 (9) | 83 | 68 (10) | 0.088 |  |

SD = standard deviation;
* Mixed model p-value for group effect adjusted for age, gender and baseline pre-operative values

**Stata output**

**Overall mixed model**

Mixed-effects ML regression Number of obs = 401

Group variable: regid2 Number of groups = 169

Obs per group:

min = 1

avg = 2.4

max = 3

Wald chi2(7) = 75.24

Log likelihood = -1457.0361 Prob > chi2 = 0.0000

------------------------------------------------------------------------------

cs | Coefficient Std. err. z P>|z| [95% conf. interval]

-------------+----------------------------------------------------------------

cs0 | .150219 .056723 2.65 0.008 .039044 .261394

_Ievent_12 | 4.132506 .7966308 5.19 0.000 2.571139 5.693874

_Ievent_24 | 6.221686 .8365738 7.44 0.000 4.582032 7.861341

Age | -.2089571 .122488 -1.71 0.088 -.449029 .0311149

gender | 1.782598 2.4949 0.71 0.475 -3.107317 6.672513

_Igroup_2 | 2.297325 2.911386 0.79 0.430 -3.408886 8.003537

_Igroup_3 | -1.06137 2.013145 -0.53 0.598 -5.007062 2.884323

_cons | 73.88214 9.618942 7.68 0.000 55.02936 92.73492

------------------------------------------------------------------------------

( 1) [cs]_Igroup_2 = 0

( 2) [cs]_Igroup_3 = 0

chi2( 2) = 1.53

Prob > chi2 = 0.4659

**Adjusted model at 6 months**

**------------------------------------------------------------------------------**

**cs | Coefficient Std. err. t P>|t| [95% conf. interval]**

**-------------+----------------------------------------------------------------**

**cs0 | .1246344 .0759539 1.64 0.103 -.0255496 .2748184**

**_Igroup_2 | -1.59335 4.670141 -0.34 0.733 -10.82764 7.640936**

**_Igroup_3 | -2.757861 2.622655 -1.05 0.295 -7.943647 2.427925**

**Age | -.272464 .1577425 -1.73 0.086 -.5843687 .0394407**

**gender | .3333821 3.52326 0.09 0.925 -6.633172 7.299936**

**_cons | 80.97584 12.42917 6.51 0.000 56.39961 105.5521**

**------------------------------------------------------------------------------**

**( 1) _Igroup_2 = 0**

**( 2) _Igroup_3 = 0**

**F( 2, 138) = 0.55**

**Prob > F = 0.5762**

**Adjusted model at 12 months**

**------------------------------------------------------------------------------**

**cs | Coefficient Std. err. t P>|t| [95% conf. interval]**

**-------------+----------------------------------------------------------------**

**cs0 | .2180033 .0736308 2.96 0.004 .0723441 .3636626**

**_Igroup_2 | -1.325041 3.840913 -0.34 0.731 -8.923284 6.273202**

**_Igroup_3 | -2.812907 2.517994 -1.12 0.266 -7.794101 2.168286**

**Age | -.1577327 .150871 -1.05 0.298 -.4561916 .1407262**

**gender | .9715522 3.254104 0.30 0.766 -5.465842 7.408947**

**_cons | 73.61862 11.92979 6.17 0.000 50.01865 97.21858**

**------------------------------------------------------------------------------**

**( 1) _Igroup_2 = 0**

**( 2) _Igroup_3 = 0**

**F( 2, 131) = 0.63**

**Prob > F = 0.5333**

**Adjusted model at 24 months**

**------------------------------------------------------------------------------**

**cs | Coefficient Std. err. t P>|t| [95% conf. interval]**

**-------------+----------------------------------------------------------------**

**cs0 | .1037975 .0647075 1.60 0.111 -.0243876 .2319826**

**_Igroup_2 | 6.836885 3.101641 2.20 0.030 .6925583 12.98121**

**_Igroup_3 | 2.020042 2.48169 0.81 0.417 -2.896167 6.93625**

**Age | -.0905872 .1477395 -0.61 0.541 -.3832579 .2020835**

**gender | 5.138864 2.717986 1.89 0.061 -.2454458 10.52317**

**_cons | 69.34874 11.40202 6.08 0.000 46.76142 91.93606**

**------------------------------------------------------------------------------**

**( 1) _Igroup_2 = 0**

**( 2) _Igroup_3 = 0**

**F( 2, 114) = 2.48**

**Prob > F = 0.0884**

**Baseline and postoperative shoulder range of motion (ROM) parameters, strength and functional scores**

|  | **155** |  | **145** |  | **135** |  | Adjusted | Model |
| --- | --- | --- | --- | --- | --- | --- | --- | --- |
| Strength, pain level and functional scores | n | mean (SD) | n | mean (SD) | n | mean (SD) | p-value | p-value* |
| SPADI (0=worst, 100=best) |  |  |  |  |  |  |  | 0.252 |
| Baseline | 46 | 33 (22) | 18 | 40 (19) | 128 | 34 (20) |  |  |
| 6 months | 50 | 73 (20) | 15 | 84 (14) | 131 | 78 (18) | 0.199 |  |
| 12 months | 44 | 76 (19) | 14 | 83 (14) | 130 | 82 (18) | 0.598 |  |
| 24 months | 47 | 74 (21) | 17 | 83 (15) | 129 | 80 (20) | 0.534 |  |

SD = standard deviation;
* Mixed model p-value for group effect adjusted for age, gender and baseline pre-operative values

**Stata output**

**Overall mixed model**

Mixed-effects ML regression Number of obs = 538

Group variable: regid2 Number of groups = 188

Obs per group:

min = 1

avg = 2.9

max = 3

Wald chi2(7) = 30.56

Log likelihood = -2201.523 Prob > chi2 = 0.0001

------------------------------------------------------------------------------

spadi | Coefficient Std. err. z P>|z| [95% conf. interval]

-------------+----------------------------------------------------------------

spadi0 | .2416513 .0580106 4.17 0.000 .1279526 .3553499

_Ievent_12 | 3.006832 1.110611 2.71 0.007 .8300749 5.18359

_Ievent_24 | 1.626029 1.101167 1.48 0.140 -.5322194 3.784278

Age | .1668051 .1808891 0.92 0.356 -.187731 .5213412

gender | -3.880645 3.797197 -1.02 0.307 -11.32301 3.561724

_Igroup_2 | 6.920023 4.591201 1.51 0.132 -2.078566 15.91861

_Igroup_3 | 3.764341 2.967172 1.27 0.205 -2.05121 9.579892

_cons | 53.87833 14.22961 3.79 0.000 25.9888 81.76785

------------------------------------------------------------------------------

( 1) [spadi]_Igroup_2 = 0

( 2) [spadi]_Igroup_3 = 0

chi2( 2) = 2.75

Prob > chi2 = 0.2524

**Adjusted model at 6 months**

**------------------------------------------------------------------------------**

**spadi | Coefficient Std. err. t P>|t| [95% conf. interval]**

**-------------+----------------------------------------------------------------**

**spadi0 | .2727358 .0671879 4.06 0.000 .1401432 .4053283**

**_Igroup_2 | 9.562839 5.361622 1.78 0.076 -1.018091 20.14377**

**_Igroup_3 | 3.196306 3.353471 0.95 0.342 -3.421626 9.814238**

**Age | .1866994 .2066773 0.90 0.368 -.2211694 .5945682**

**gender | -4.919437 4.305282 -1.14 0.255 -13.41573 3.576852**

**_cons | 51.49653 16.2068 3.18 0.002 19.5131 83.47995**

**------------------------------------------------------------------------------**

**( 1) _Igroup_2 = 0**

**( 2) _Igroup_3 = 0**

**F( 2, 177) = 1.63**

**Prob > F = 0.1994**

**Adjusted model at 12 months**

**------------------------------------------------------------------------------**

**spadi | Coefficient Std. err. t P>|t| [95% conf. interval]**

**-------------+----------------------------------------------------------------**

**spadi0 | .2204423 .0653877 3.37 0.001 .0913605 .3495241**

**_Igroup_2 | 3.977596 5.415166 0.73 0.464 -6.712486 14.66768**

**_Igroup_3 | 3.156723 3.354482 0.94 0.348 -3.465362 9.778808**

**Age | .0524503 .201676 0.26 0.795 -.3456783 .450579**

**gender | -5.058197 4.319512 -1.17 0.243 -13.58535 3.468953**

**_cons | 67.14438 15.84169 4.24 0.000 35.87128 98.41747**

**------------------------------------------------------------------------------**

**( 1) _Igroup_2 = 0**

**( 2) _Igroup_3 = 0**

**F( 2, 169) = 0.52**

**Prob > F = 0.5977**

**Adjusted model at 24 months**

**------------------------------------------------------------------------------**

**spadi | Coefficient Std. err. t P>|t| [95% conf. interval]**

**-------------+----------------------------------------------------------------**

**spadi0 | .2407613 .0710538 3.39 0.001 .100523 .3809996**

**_Igroup_2 | 5.922393 5.710307 1.04 0.301 -5.347991 17.19278**

**_Igroup_3 | 3.085559 3.723031 0.83 0.408 -4.262555 10.43367**

**Age | .2139077 .2252723 0.95 0.344 -.2307102 .6585257**

**gender | -3.312355 4.839182 -0.68 0.495 -12.86341 6.238698**

**_cons | 52.52027 17.84585 2.94 0.004 17.29807 87.74248**

**------------------------------------------------------------------------------**

**( 1) _Igroup_2 = 0**

**( 2) _Igroup_3 = 0**

**F( 2, 174) = 0.63**

**Prob > F = 0.5336**

**Baseline and postoperative shoulder range of motion (ROM) parameters, strength and functional scores**

|  | **155** |  | **145** |  | **135** |  | Adjusted | Model |
| --- | --- | --- | --- | --- | --- | --- | --- | --- |
| Strength, pain level and functional scores | n | mean (SD) | n | mean (SD) | n | mean (SD) | p-value | p-value* |
| Subjective Shoulder Value (0=worst, 100=best) |  |  |  |  |  |  |  | 0.325 |
| Baseline | 38 | 41 (20) | 34 | 34 (20) | 118 | 39 (20) |  |  |
| 6 months | 41 | 74 (18) | 12 | 78 (13) | 112 | 78 (16) | 0.856 |  |
| 12 months | 41 | 75 (18) | 26 | 78 (14) | 117 | 83 (13) | 0.110 |  |
| 24 months | 40 | 77 (18) | 34 | 85 (13) | 114 | 83 (14) | 0.323 |  |

SD = standard deviation;
* Mixed model p-value for group effect adjusted for age, gender and baseline pre-operative values

**Stata output**

**Overall mixed model**

Mixed-effects ML regression Number of obs = 467

Group variable: regid2 Number of groups = 185

Obs per group:

min = 1

avg = 2.5

max = 3

Wald chi2(7) = 27.76

Log likelihood = -1876.1257 Prob > chi2 = 0.0002

------------------------------------------------------------------------------

ssv | Coefficient Std. err. z P>|z| [95% conf. interval]

-------------+----------------------------------------------------------------

ssv0 | .1392634 .047192 2.95 0.003 .0467688 .231758

_Ievent_12 | 3.251282 1.241172 2.62 0.009 .8186307 5.683934

_Ievent_24 | 4.858623 1.24403 3.91 0.000 2.420368 7.296877

Age | .1308352 .1486003 0.88 0.379 -.160416 .4220864

gender | -1.901315 2.99406 -0.64 0.525 -7.769564 3.966935

_Igroup_2 | 4.363013 3.111267 1.40 0.161 -1.734958 10.46098

_Igroup_3 | 3.130001 2.545879 1.23 0.219 -1.85983 8.119832

_cons | 59.37792 11.67936 5.08 0.000 36.48679 82.26906

------------------------------------------------------------------------------

( 1) [ssv]_Igroup_2 = 0

( 2) [ssv]_Igroup_3 = 0

chi2( 2) = 2.25

Prob > chi2 = 0.3253

**Adjusted model at 6 months**

**------------------------------------------------------------------------------**

**ssv | Coefficient Std. err. t P>|t| [95% conf. interval]**

**-------------+----------------------------------------------------------------**

**ssv0 | .252104 .0725389 3.48 0.001 .1086443 .3955636**

**_Igroup_2 | 3.122909 5.625032 0.56 0.580 -8.001673 14.24749**

**_Igroup_3 | .6940567 3.615977 0.19 0.848 -6.457234 7.845347**

**Age | .206623 .2181861 0.95 0.345 -.224882 .638128**

**gender | -6.813807 4.915256 -1.39 0.168 -16.53467 2.907056**

**_cons | 51.3126 17.06099 3.01 0.003 17.57121 85.05399**

**------------------------------------------------------------------------------**

**( 1) _Igroup_2 = 0**

**( 2) _Igroup_3 = 0**

**F( 2, 135) = 0.16**

**Prob > F = 0.8563**

**Adjusted model at 12 months**

**------------------------------------------------------------------------------**

**ssv | Coefficient Std. err. t P>|t| [95% conf. interval]**

**-------------+----------------------------------------------------------------**

**ssv0 | .1246952 .0554456 2.25 0.026 .0151742 .2342161**

**_Igroup_2 | 2.140125 3.748981 0.57 0.569 -5.26519 9.545441**

**_Igroup_3 | 5.987618 2.953825 2.03 0.044 .1529655 11.82227**

**Age | .091367 .1788134 0.51 0.610 -.2618409 .4445748**

**gender | -1.500944 3.544341 -0.42 0.673 -8.502036 5.500148**

**_cons | 64.8699 14.0732 4.61 0.000 37.07129 92.66851**

**------------------------------------------------------------------------------**

**( 1) _Igroup_2 = 0**

**( 2) _Igroup_3 = 0**

**F( 2, 156) = 2.24**

**Prob > F = 0.1097**

**Adjusted model at 24 months**

**------------------------------------------------------------------------------**

**ssv | Coefficient Std. err. t P>|t| [95% conf. interval]**

**-------------+----------------------------------------------------------------**

**ssv0 | .0686813 .0590987 1.16 0.247 -.048044 .1854067**

**_Igroup_2 | 5.71481 3.796286 1.51 0.134 -1.783205 13.21283**

**_Igroup_3 | 3.463009 3.387196 1.02 0.308 -3.227015 10.15303**

**Age | .0617463 .1825038 0.34 0.736 -.2987156 .4222081**

**gender | 1.805731 3.766293 0.48 0.632 -5.633044 9.244506**

**_cons | 71.3016 14.53492 4.91 0.000 42.5938 100.0094**

**------------------------------------------------------------------------------**

**( 1) _Igroup_2 = 0**

**( 2) _Igroup_3 = 0**

**F( 2, 158) = 1.14**

**Prob > F = 0.3229**
