# Supplementary figures and images for: Analysis of three different reverse shoulder arthroplasty designs for cuff tear arthropathy – the combination of lateralization and distalization provides best mobility
Source: BMC Musculoskelet Disord. 2024 Mar 7;25:204. doi: 10.1186/s12891-024-07312-5 (PMC10918945; doi:10.1186/s12891-024-07312-5)

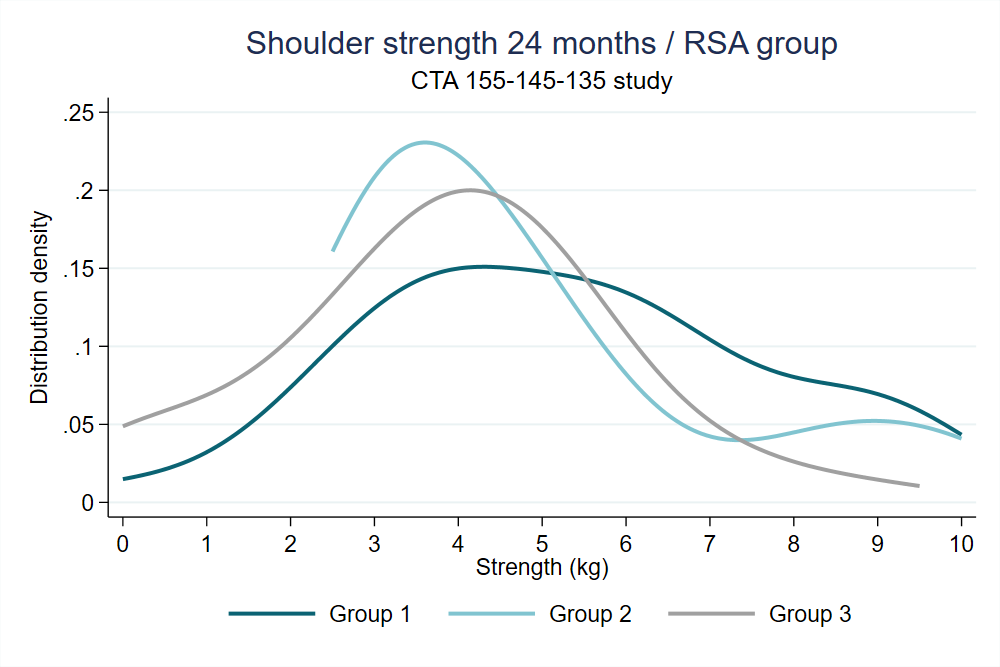

Supplement: Supplementary file 2 — Supplementary Material 2. [file 12891_2024_7312_MOESM2_ESM.zip › saroa-cta-02b-outcome-mixed-abdkraft_b-KdensCeiling24mo.png]

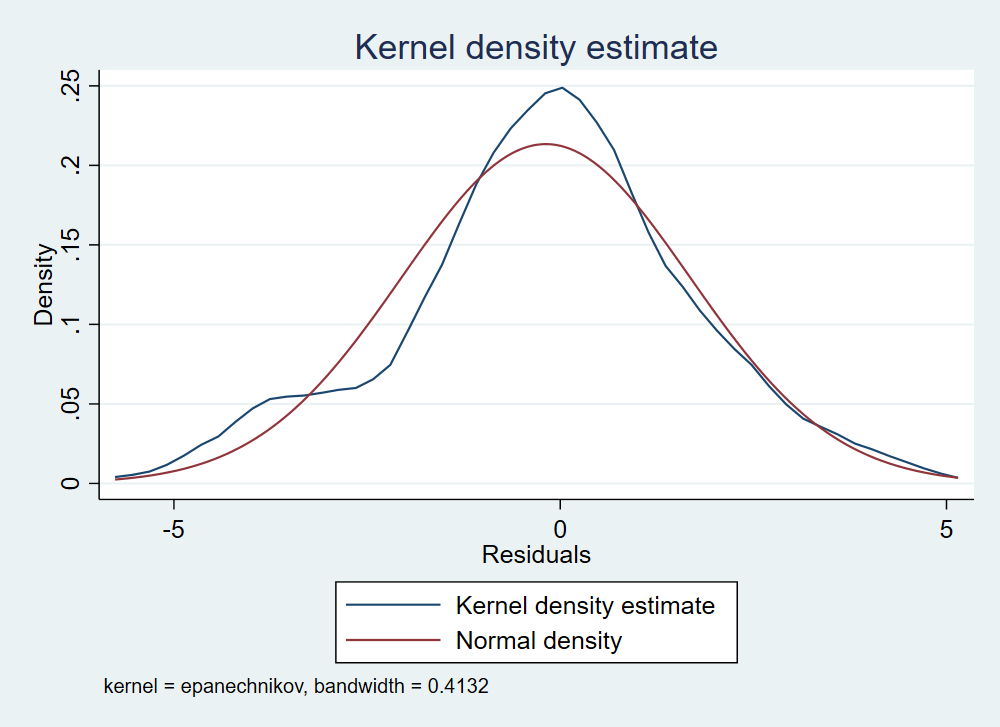

Supplement: Supplementary file 2 — Supplementary Material 2. [file 12891_2024_7312_MOESM2_ESM.zip › saroa-cta-02b-outcome-mixed-abdkraft_b-kdensity-12.png]

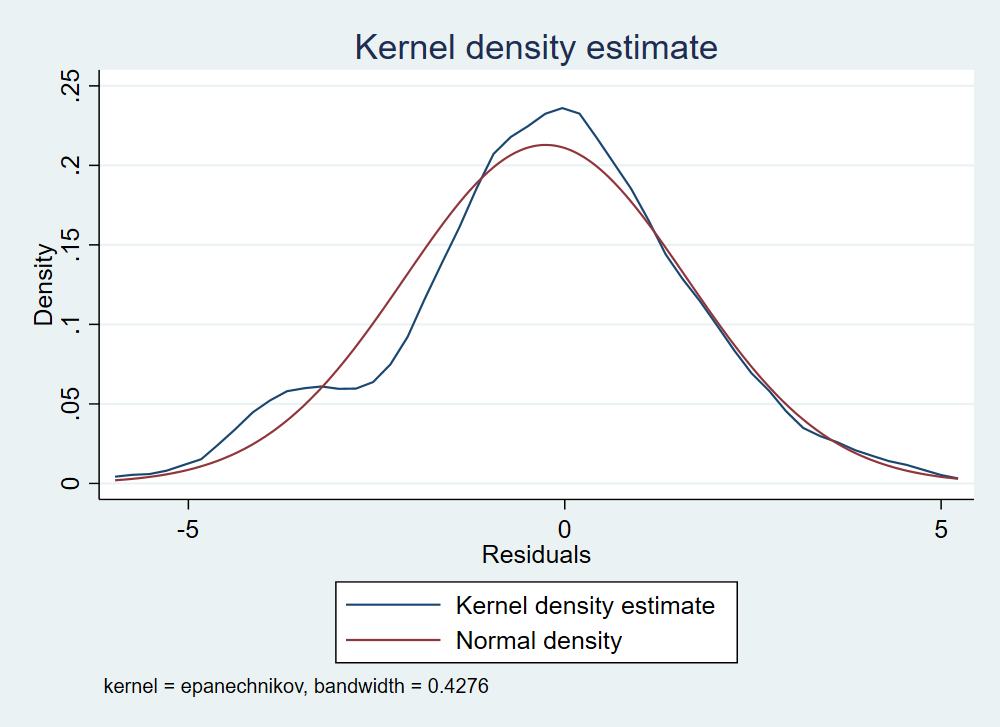

Supplement: Supplementary file 2 — Supplementary Material 2. [file 12891_2024_7312_MOESM2_ESM.zip › saroa-cta-02b-outcome-mixed-abdkraft_b-kdensity-24.png]

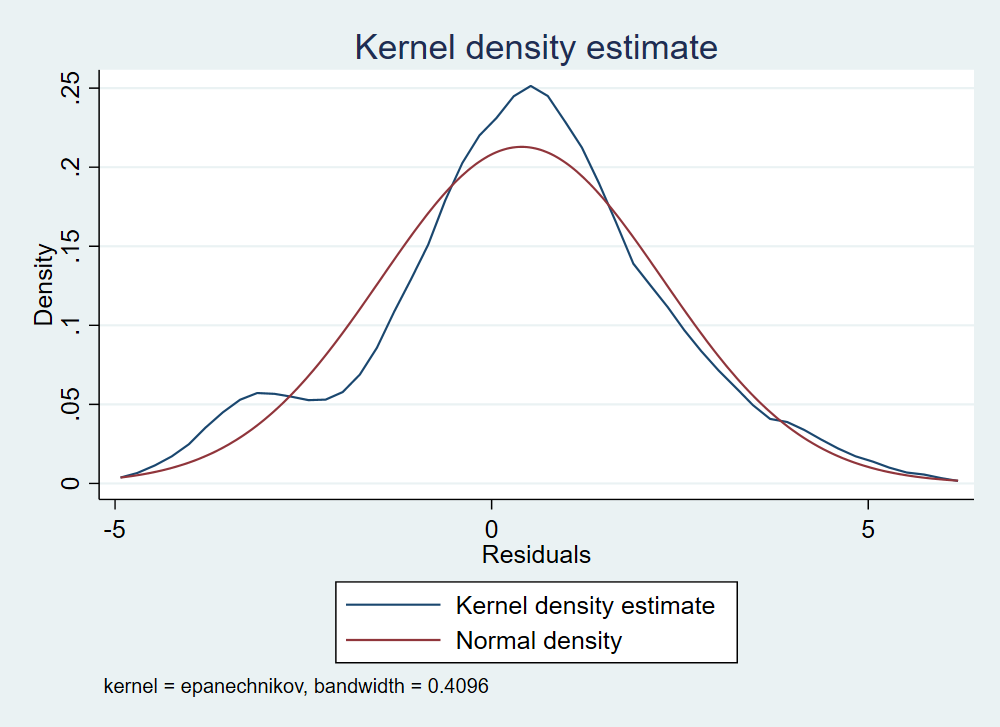

Supplement: Supplementary file 2 — Supplementary Material 2. [file 12891_2024_7312_MOESM2_ESM.zip › saroa-cta-02b-outcome-mixed-abdkraft_b-kdensity-6.png]

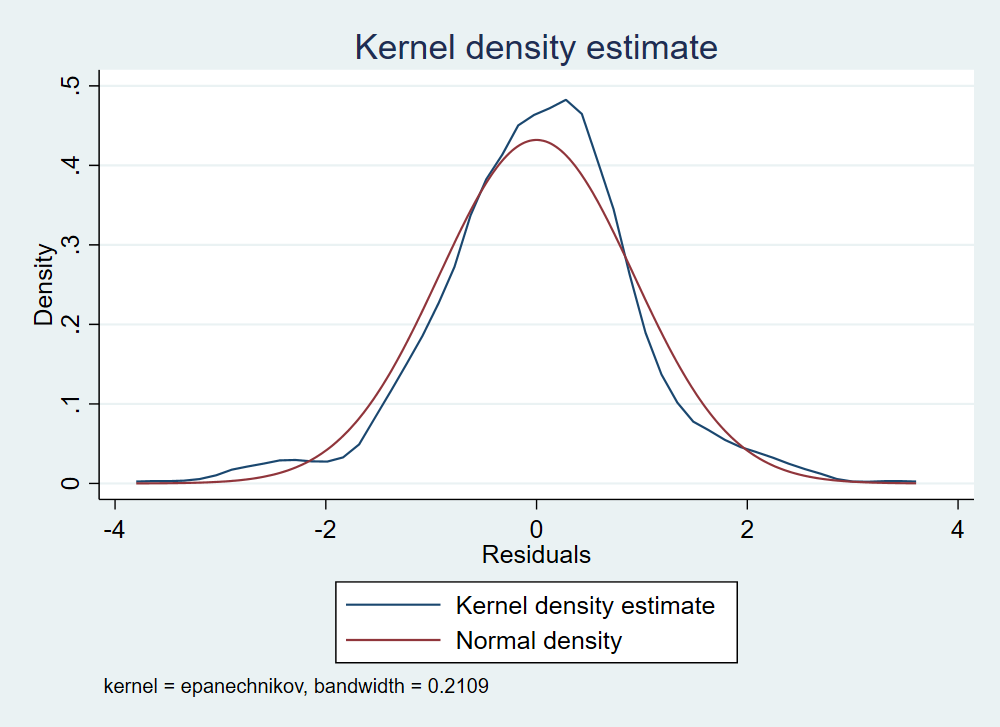

Supplement: Supplementary file 2 — Supplementary Material 2. [file 12891_2024_7312_MOESM2_ESM.zip › saroa-cta-02b-outcome-mixed-abdkraft_b-kdensity-mixed.png]

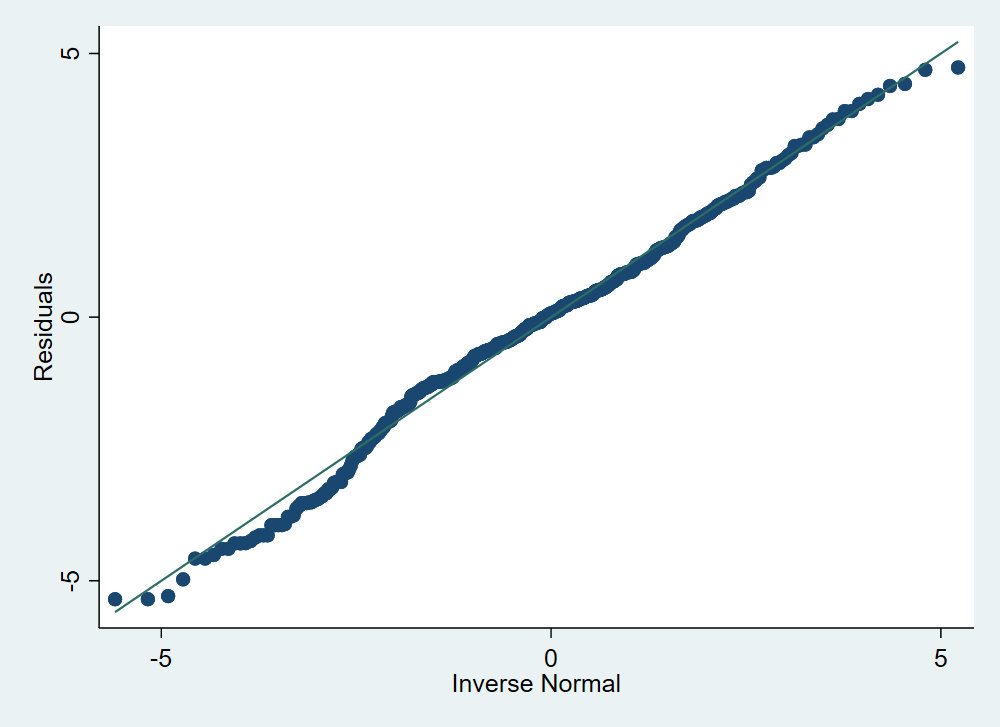

Supplement: Supplementary file 2 — Supplementary Material 2. [file 12891_2024_7312_MOESM2_ESM.zip › saroa-cta-02b-outcome-mixed-abdkraft_b-qnorm-12.png]

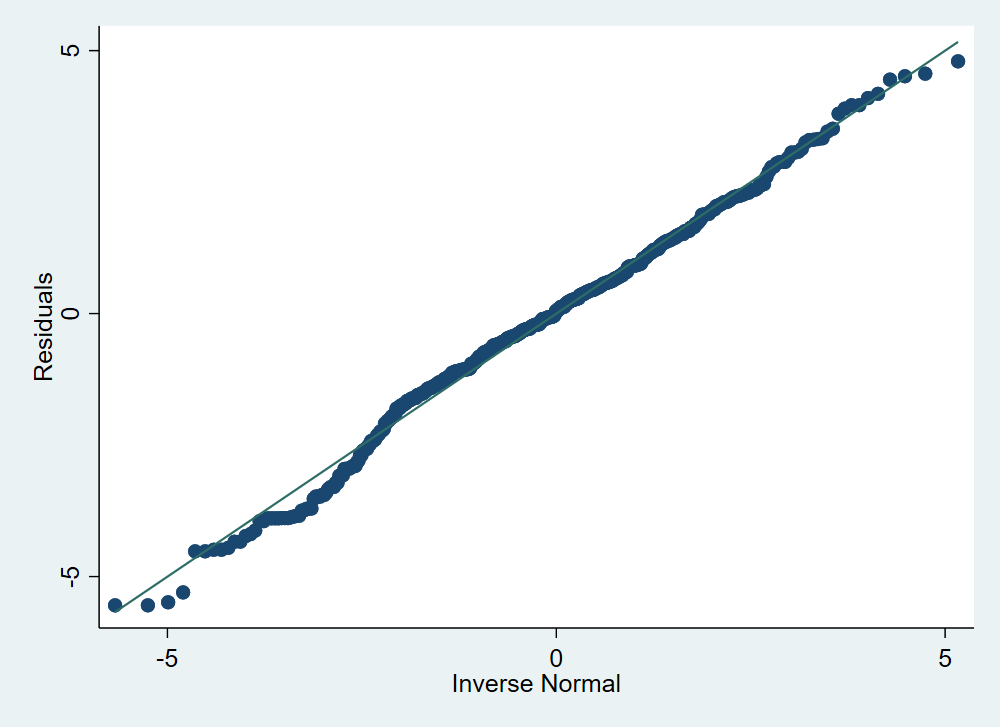

Supplement: Supplementary file 2 — Supplementary Material 2. [file 12891_2024_7312_MOESM2_ESM.zip › saroa-cta-02b-outcome-mixed-abdkraft_b-qnorm-24.png]

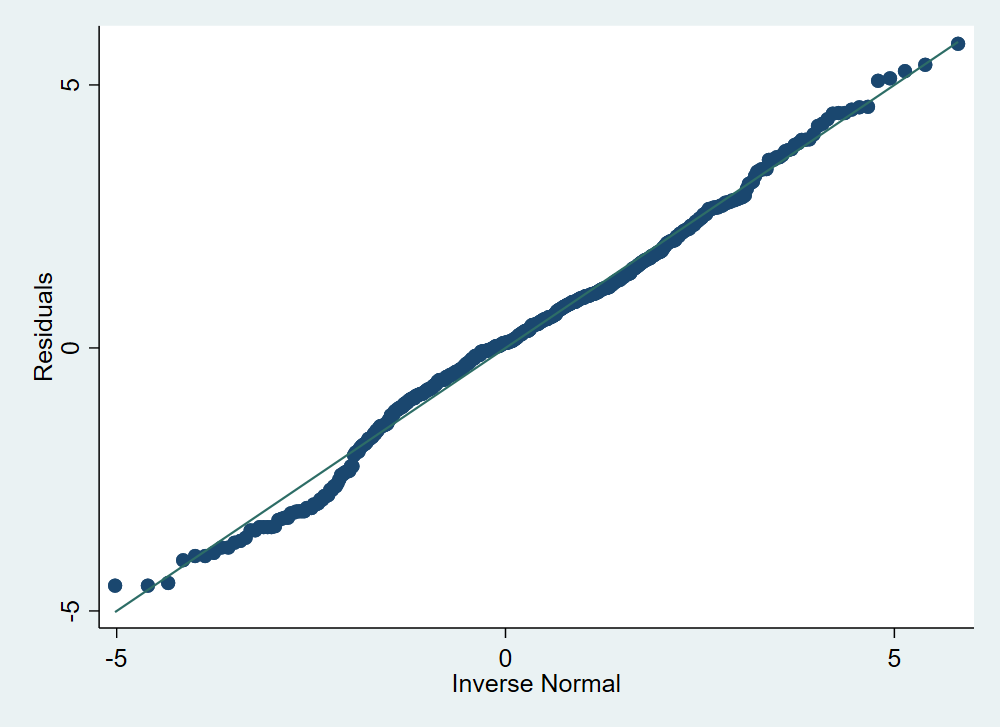

Supplement: Supplementary file 2 — Supplementary Material 2. [file 12891_2024_7312_MOESM2_ESM.zip › saroa-cta-02b-outcome-mixed-abdkraft_b-qnorm-6.png]

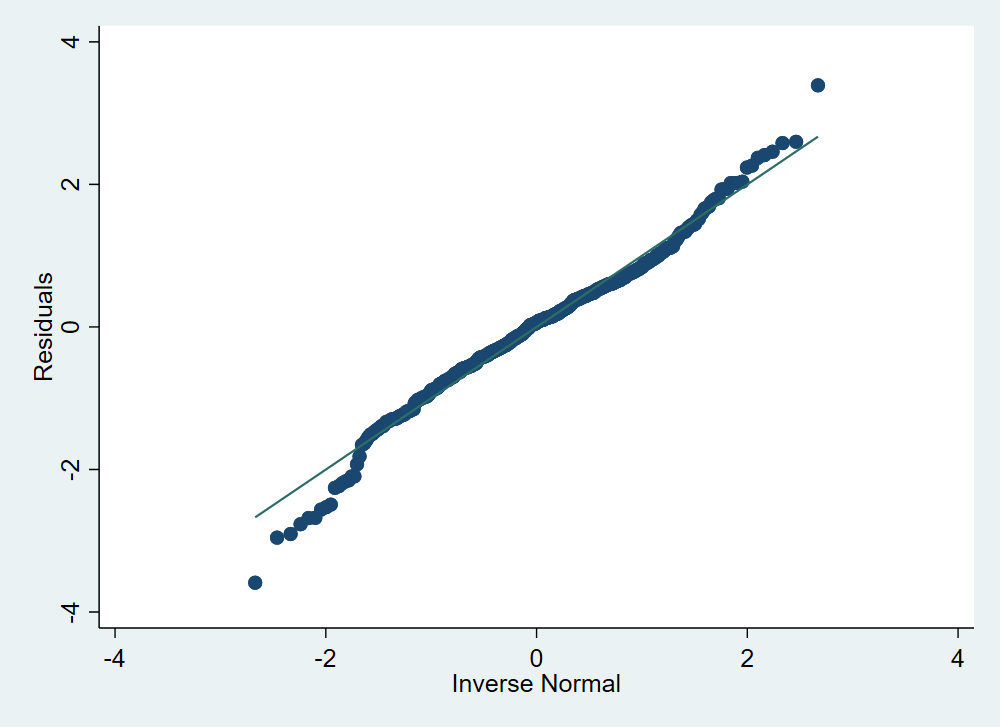

Supplement: Supplementary file 2 — Supplementary Material 2. [file 12891_2024_7312_MOESM2_ESM.zip › saroa-cta-02b-outcome-mixed-abdkraft_b-qnorm-mixed.png]

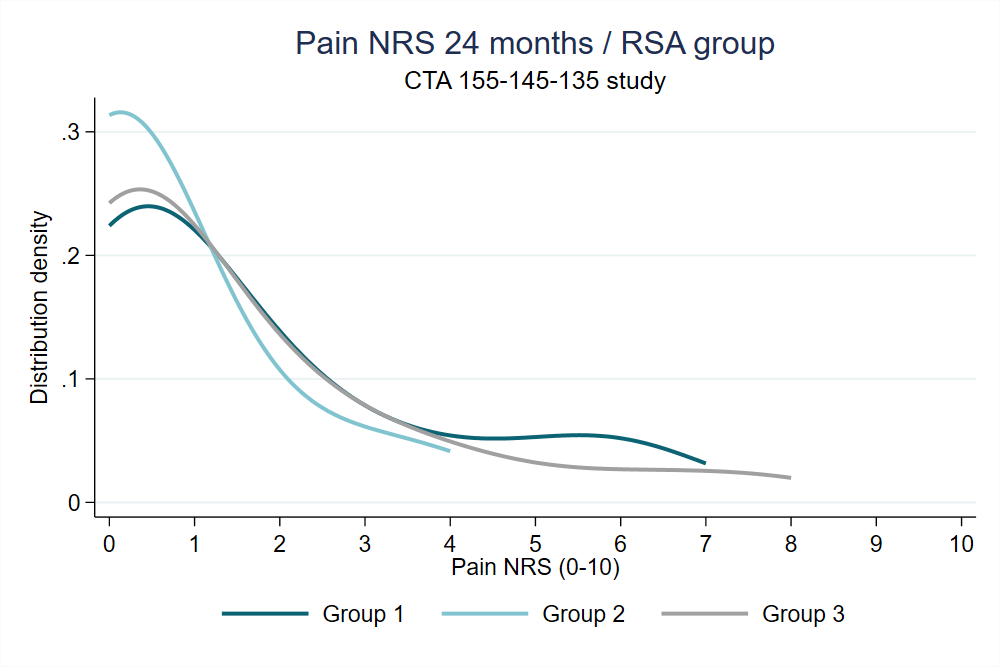

Supplement: Supplementary file 2 — Supplementary Material 2. [file 12891_2024_7312_MOESM2_ESM.zip › saroa-cta-02b-outcome-mixed-cs_01-KdensCeiling24mo.png]

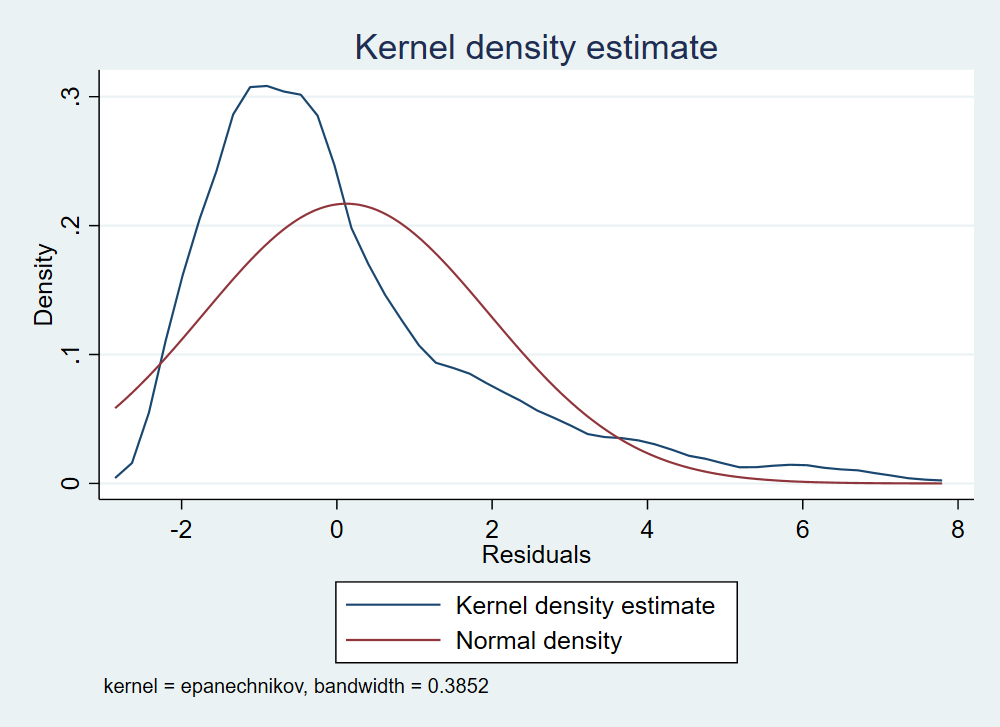

Supplement: Supplementary file 2 — Supplementary Material 2. [file 12891_2024_7312_MOESM2_ESM.zip › saroa-cta-02b-outcome-mixed-cs_01-kdensity-12.png]

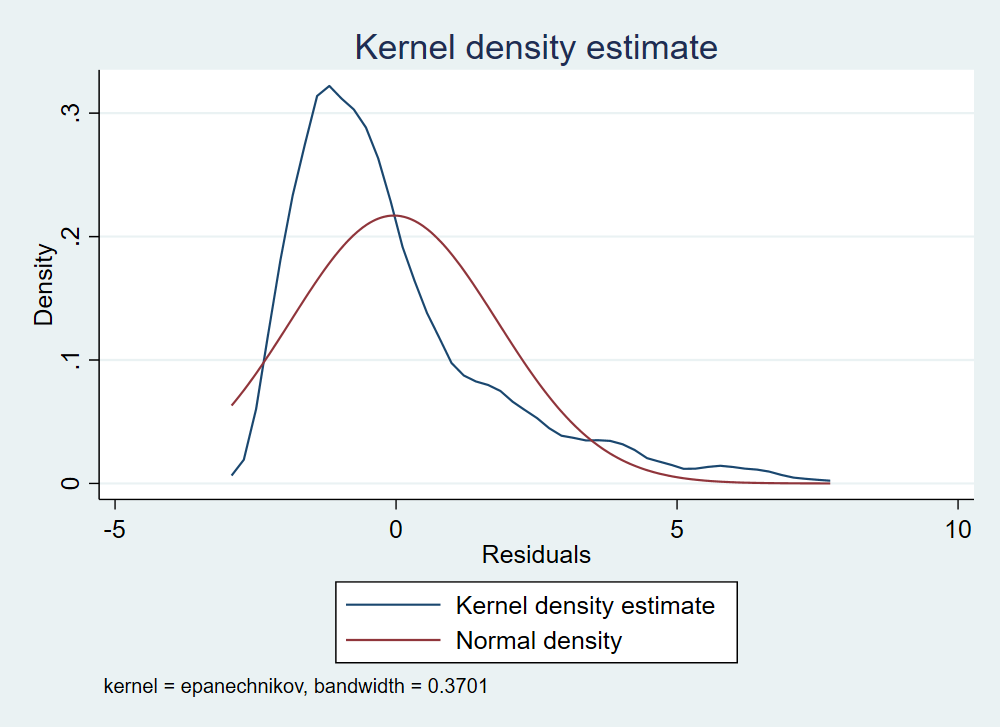

Supplement: Supplementary file 2 — Supplementary Material 2. [file 12891_2024_7312_MOESM2_ESM.zip › saroa-cta-02b-outcome-mixed-cs_01-kdensity-24.png]

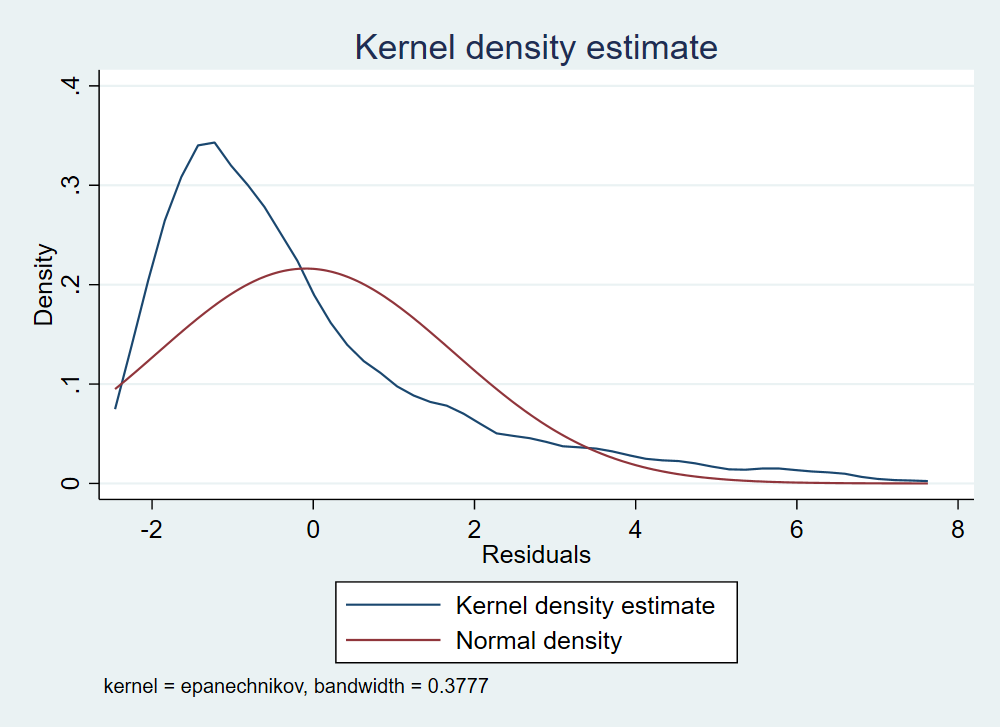

Supplement: Supplementary file 2 — Supplementary Material 2. [file 12891_2024_7312_MOESM2_ESM.zip › saroa-cta-02b-outcome-mixed-cs_01-kdensity-6.png]

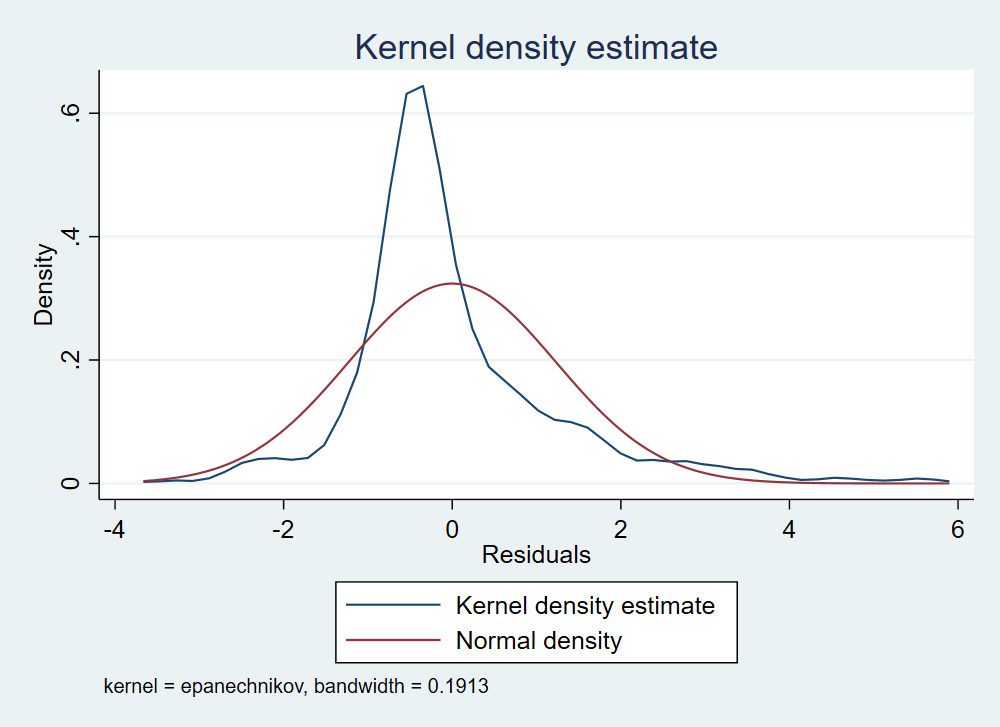

Supplement: Supplementary file 2 — Supplementary Material 2. [file 12891_2024_7312_MOESM2_ESM.zip › saroa-cta-02b-outcome-mixed-cs_01-kdensity-mixed.png]

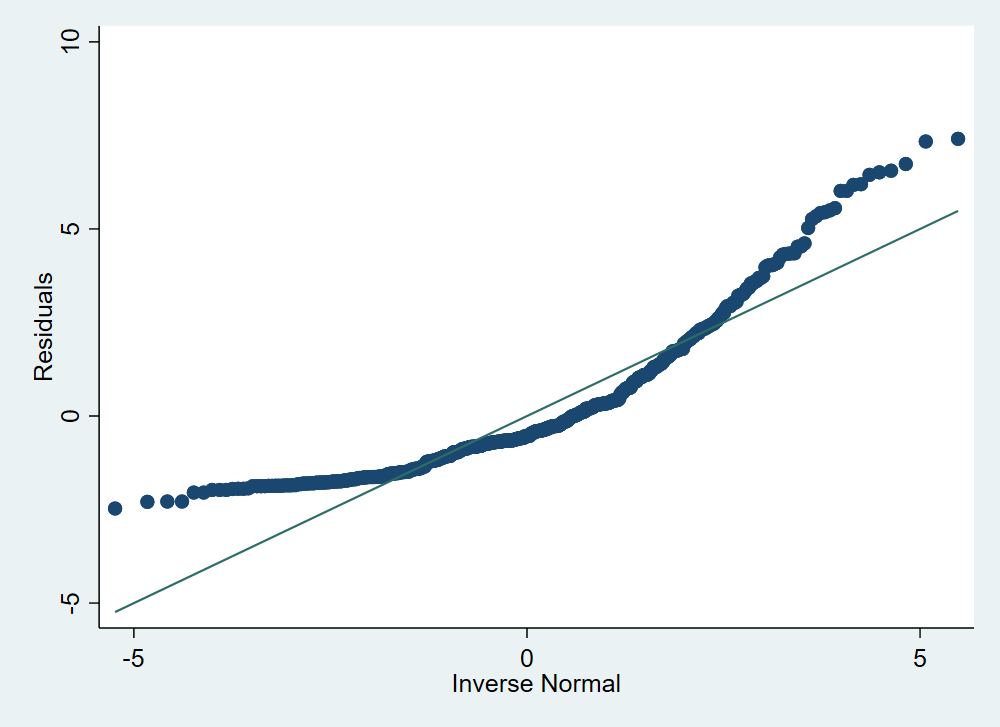

Supplement: Supplementary file 2 — Supplementary Material 2. [file 12891_2024_7312_MOESM2_ESM.zip › saroa-cta-02b-outcome-mixed-cs_01-qnorm-12.png]

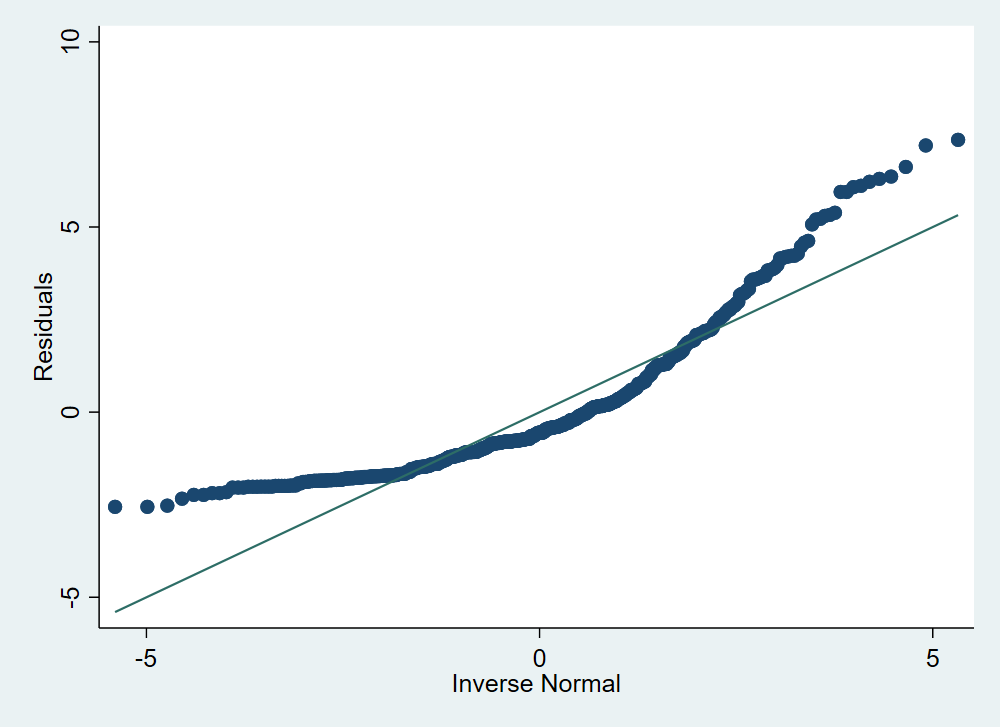

Supplement: Supplementary file 2 — Supplementary Material 2. [file 12891_2024_7312_MOESM2_ESM.zip › saroa-cta-02b-outcome-mixed-cs_01-qnorm-24.png]

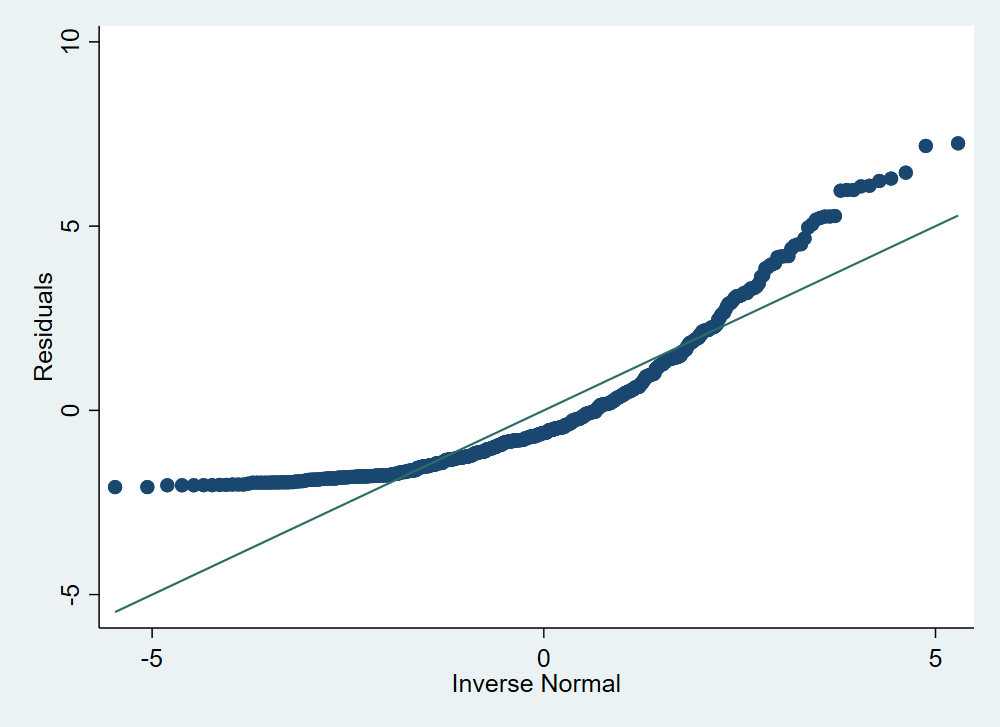

Supplement: Supplementary file 2 — Supplementary Material 2. [file 12891_2024_7312_MOESM2_ESM.zip › saroa-cta-02b-outcome-mixed-cs_01-qnorm-6.png]

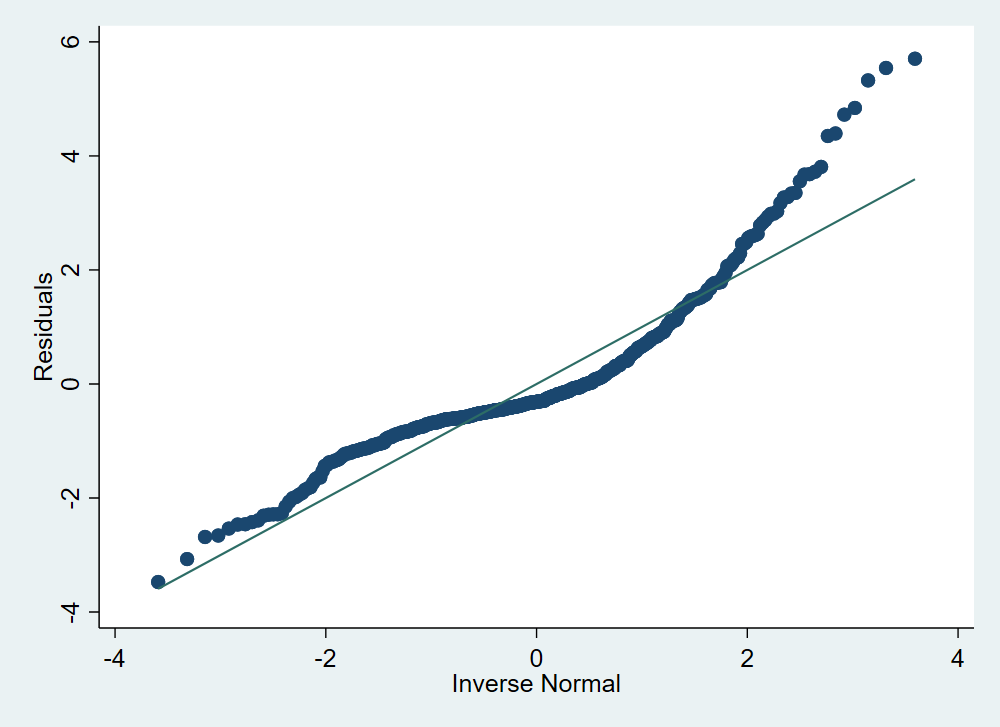

Supplement: Supplementary file 2 — Supplementary Material 2. [file 12891_2024_7312_MOESM2_ESM.zip › saroa-cta-02b-outcome-mixed-cs_01-qnorm-mixed.png]

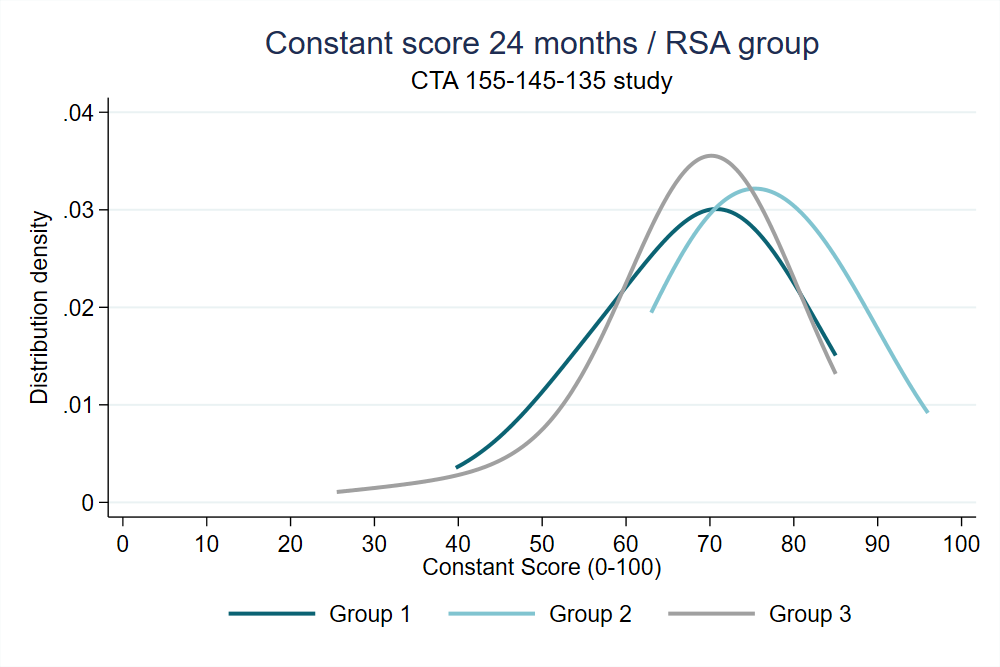

Supplement: Supplementary file 2 — Supplementary Material 2. [file 12891_2024_7312_MOESM2_ESM.zip › saroa-cta-02b-outcome-mixed-cs-KdensCeiling24mo.png]

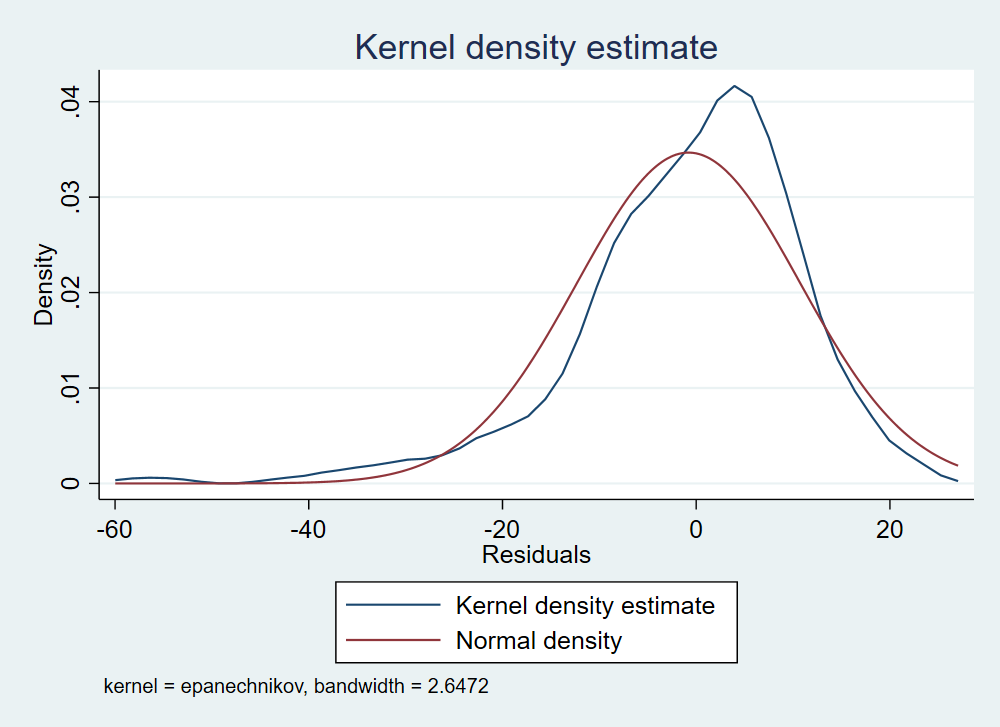

Supplement: Supplementary file 2 — Supplementary Material 2. [file 12891_2024_7312_MOESM2_ESM.zip › saroa-cta-02b-outcome-mixed-cs-kdensity-12.png]

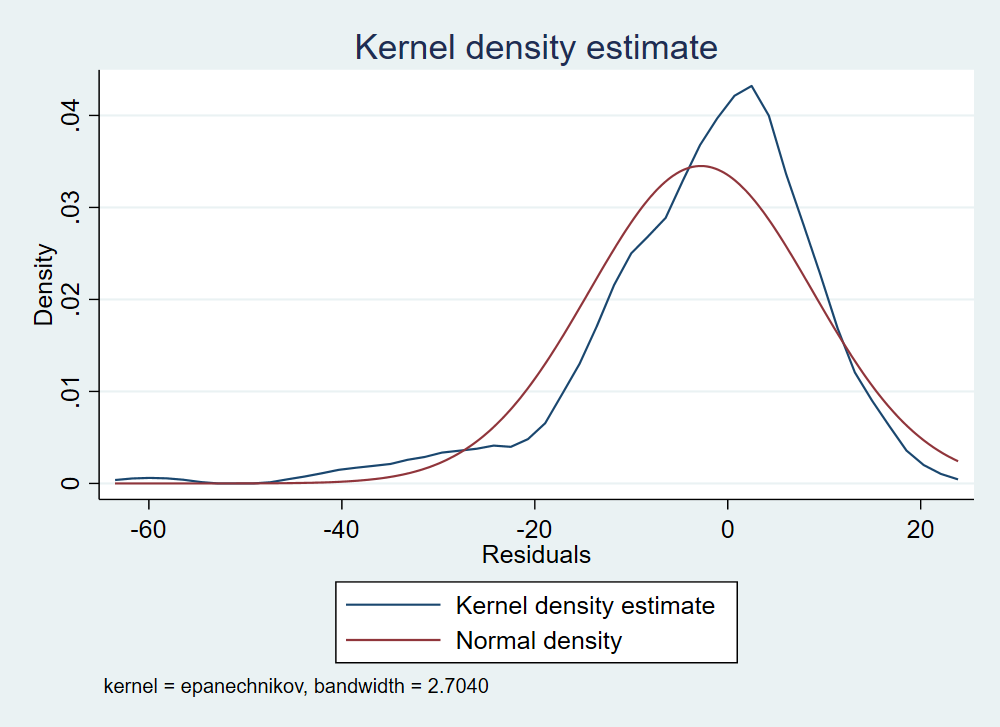

Supplement: Supplementary file 2 — Supplementary Material 2. [file 12891_2024_7312_MOESM2_ESM.zip › saroa-cta-02b-outcome-mixed-cs-kdensity-24.png]

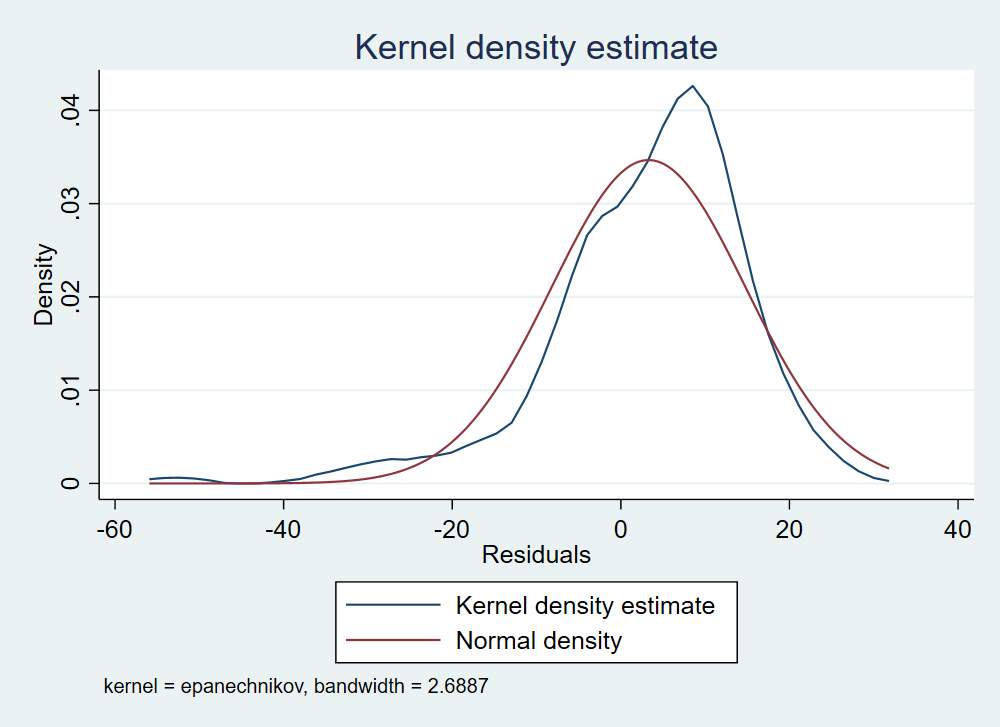

Supplement: Supplementary file 2 — Supplementary Material 2. [file 12891_2024_7312_MOESM2_ESM.zip › saroa-cta-02b-outcome-mixed-cs-kdensity-6.png]

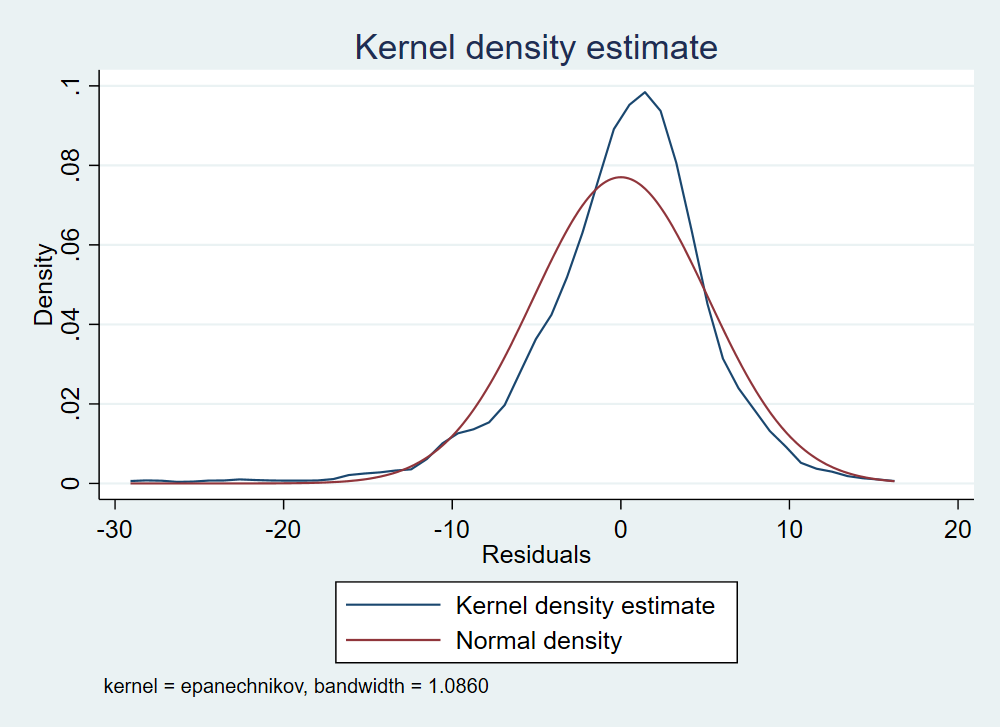

Supplement: Supplementary file 2 — Supplementary Material 2. [file 12891_2024_7312_MOESM2_ESM.zip › saroa-cta-02b-outcome-mixed-cs-kdensity-mixed.png]

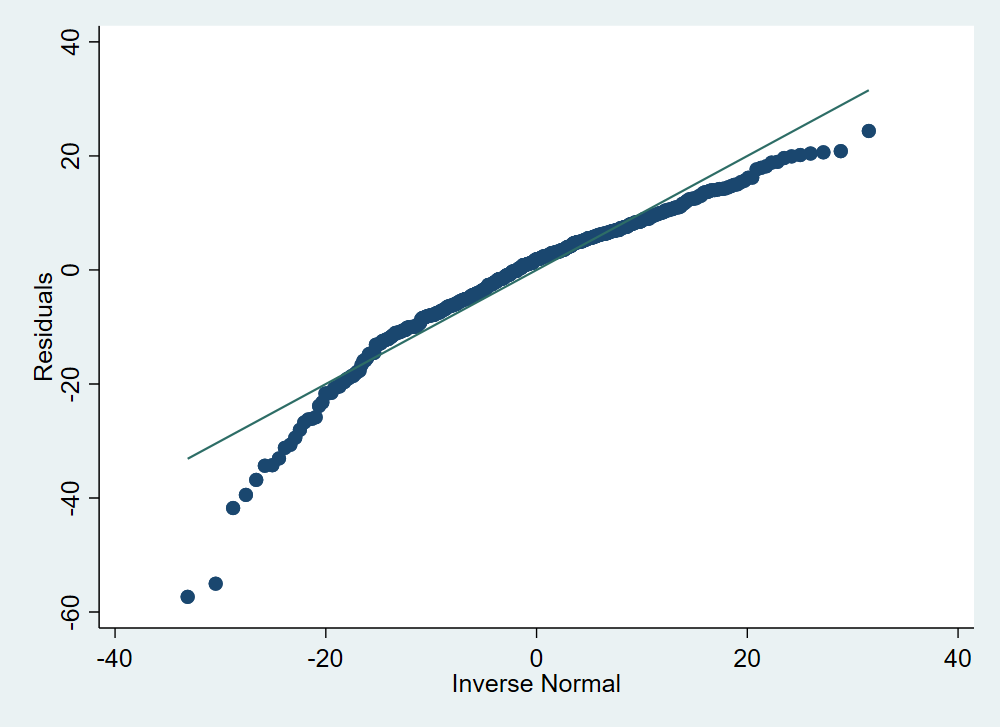

Supplement: Supplementary file 2 — Supplementary Material 2. [file 12891_2024_7312_MOESM2_ESM.zip › saroa-cta-02b-outcome-mixed-cs-qnorm-12.png]

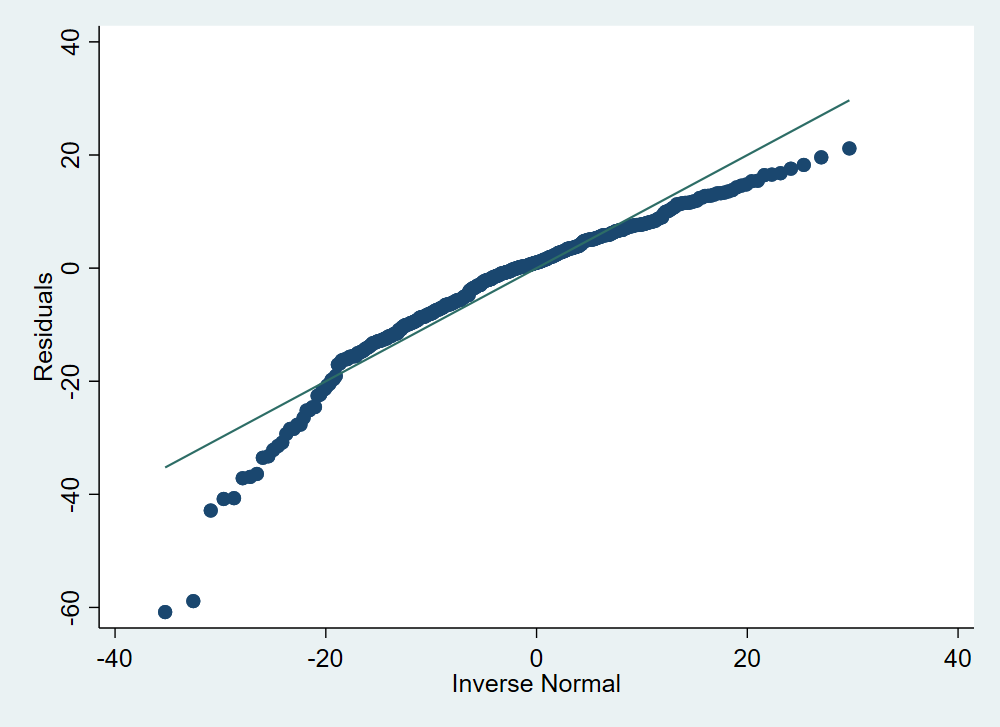

Supplement: Supplementary file 2 — Supplementary Material 2. [file 12891_2024_7312_MOESM2_ESM.zip › saroa-cta-02b-outcome-mixed-cs-qnorm-24.png]

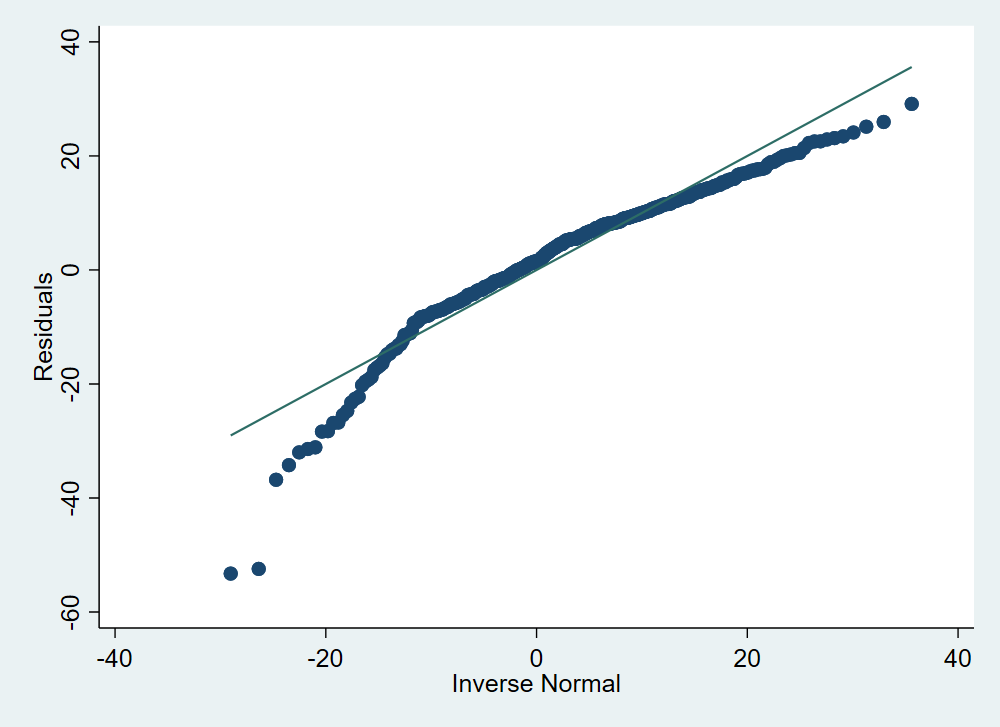

Supplement: Supplementary file 2 — Supplementary Material 2. [file 12891_2024_7312_MOESM2_ESM.zip › saroa-cta-02b-outcome-mixed-cs-qnorm-6.png]

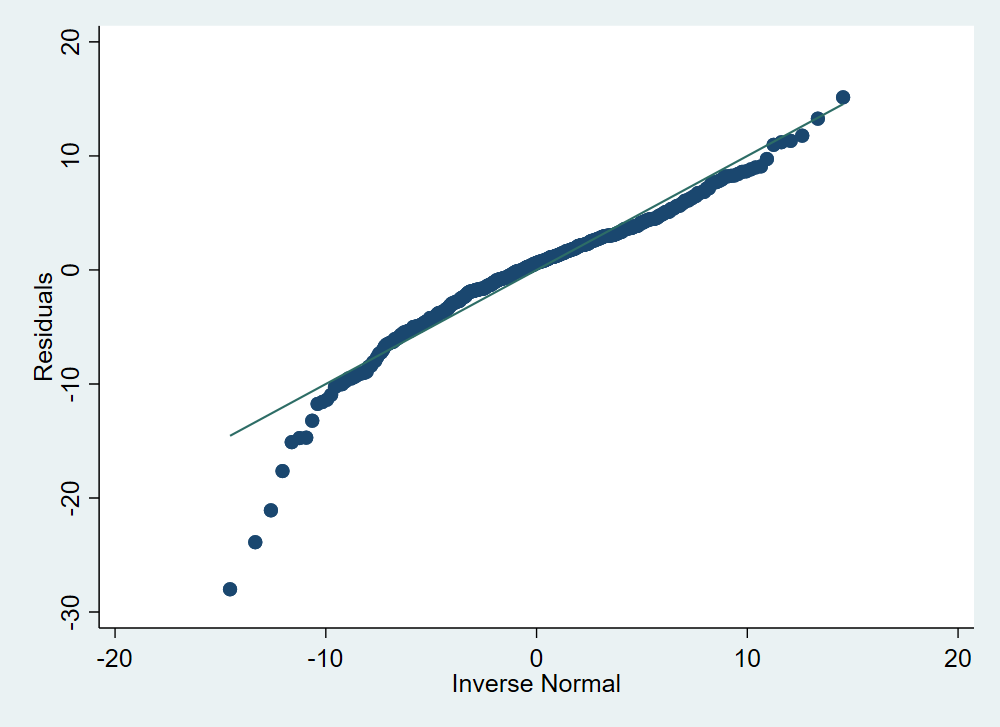

Supplement: Supplementary file 2 — Supplementary Material 2. [file 12891_2024_7312_MOESM2_ESM.zip › saroa-cta-02b-outcome-mixed-cs-qnorm-mixed.png]

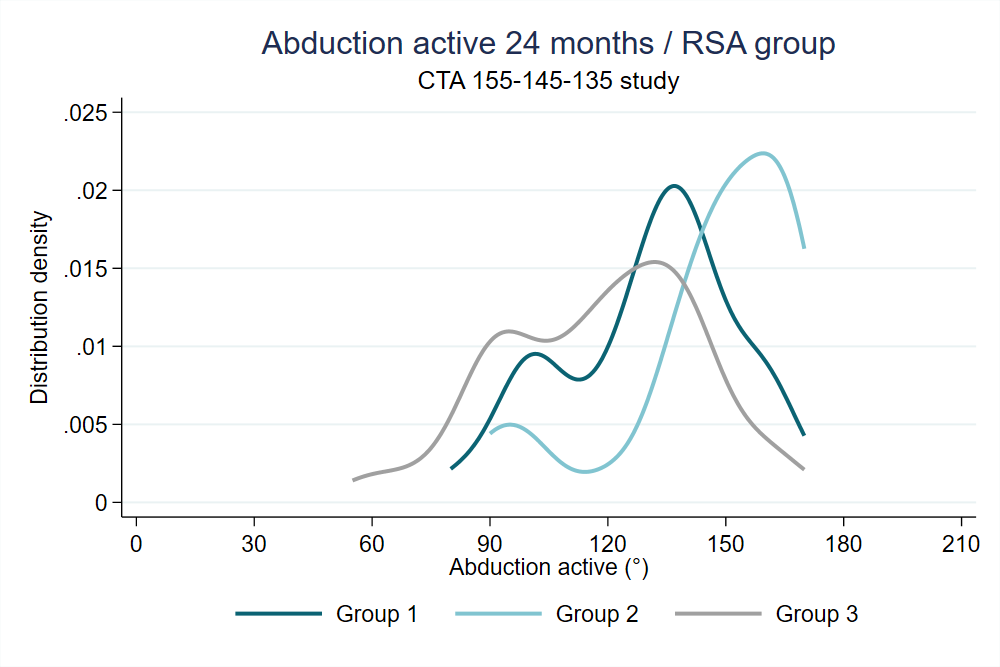

Supplement: Supplementary file 2 — Supplementary Material 2. [file 12891_2024_7312_MOESM2_ESM.zip › saroa-cta-02b-outcome-mixed-rm_abd_bakt-KdensCeiling24mo.png]

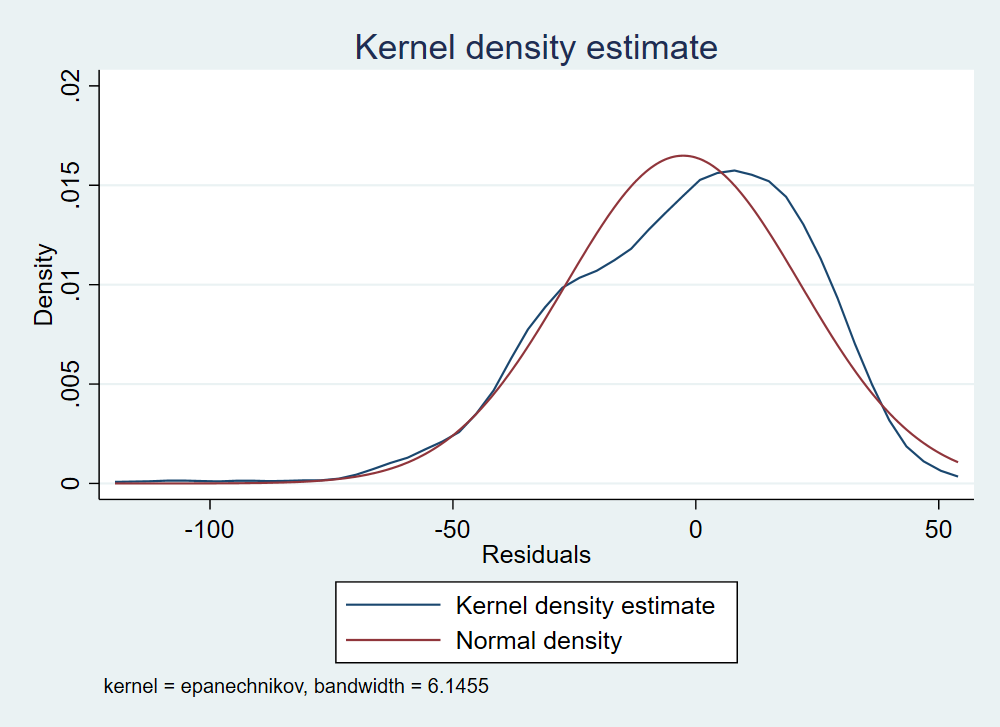

Supplement: Supplementary file 2 — Supplementary Material 2. [file 12891_2024_7312_MOESM2_ESM.zip › saroa-cta-02b-outcome-mixed-rm_abd_bakt-kdensity-12.png]

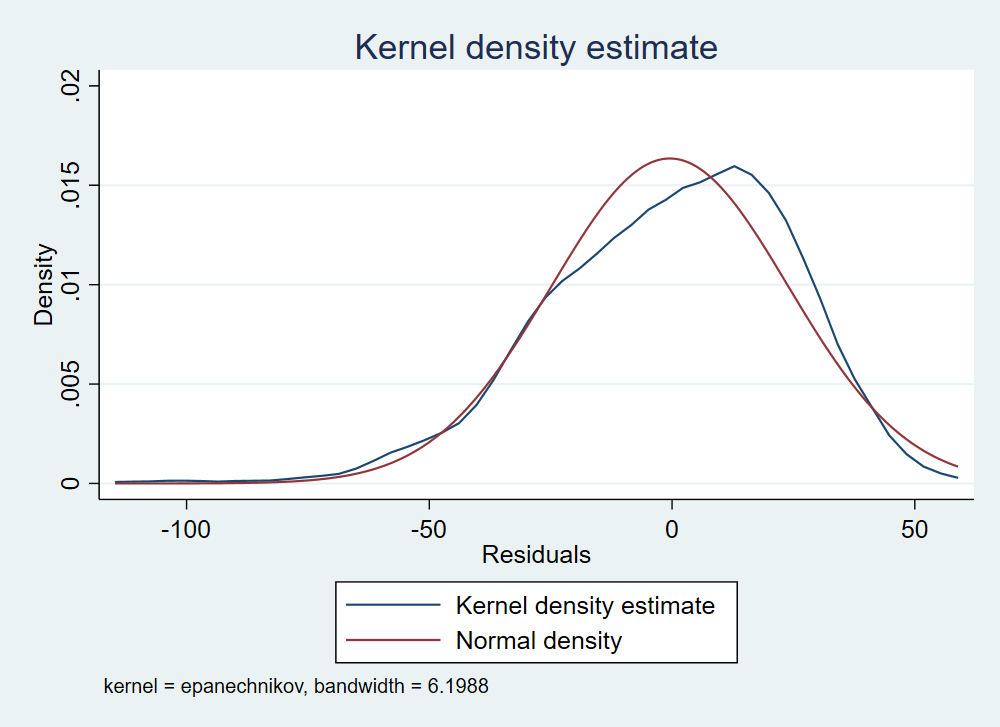

Supplement: Supplementary file 2 — Supplementary Material 2. [file 12891_2024_7312_MOESM2_ESM.zip › saroa-cta-02b-outcome-mixed-rm_abd_bakt-kdensity-24.png]

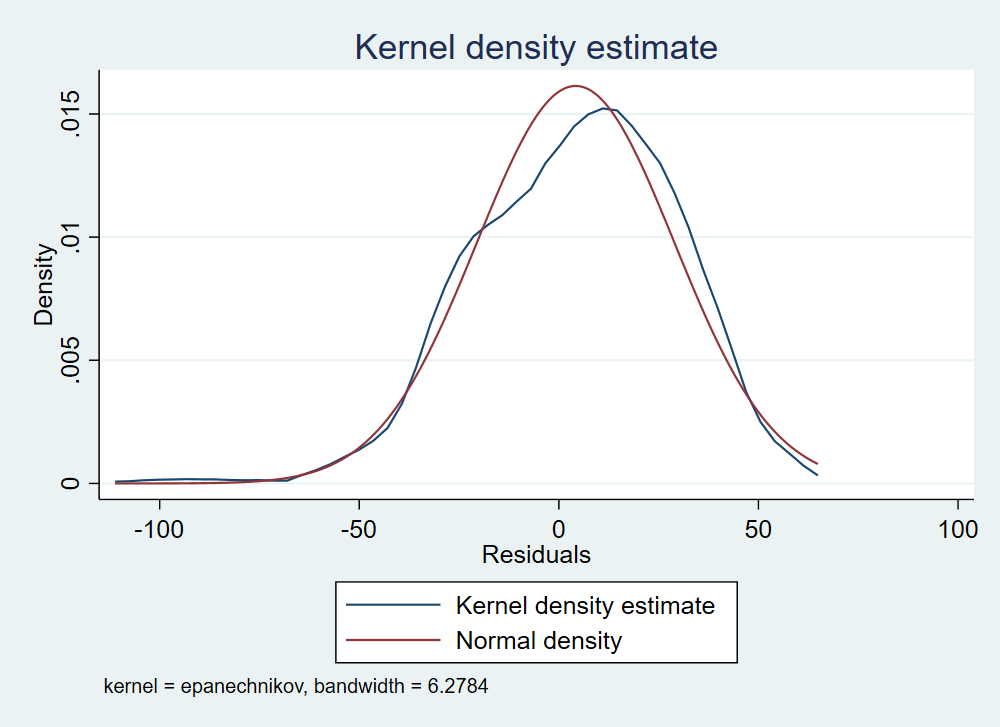

Supplement: Supplementary file 2 — Supplementary Material 2. [file 12891_2024_7312_MOESM2_ESM.zip › saroa-cta-02b-outcome-mixed-rm_abd_bakt-kdensity-6.png]

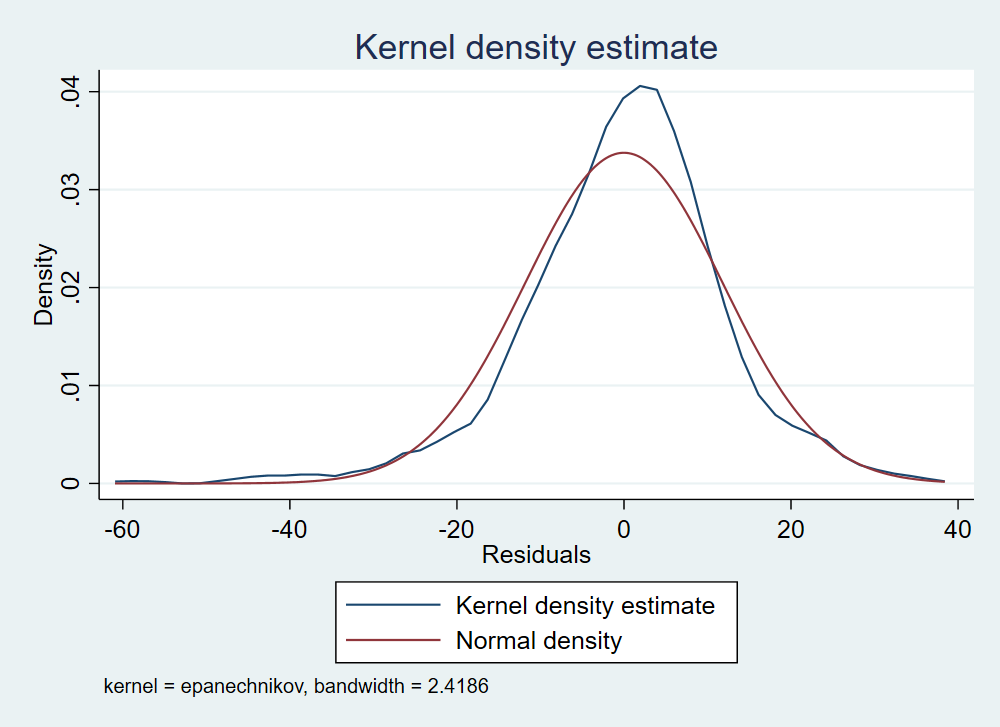

Supplement: Supplementary file 2 — Supplementary Material 2. [file 12891_2024_7312_MOESM2_ESM.zip › saroa-cta-02b-outcome-mixed-rm_abd_bakt-kdensity-mixed.png]

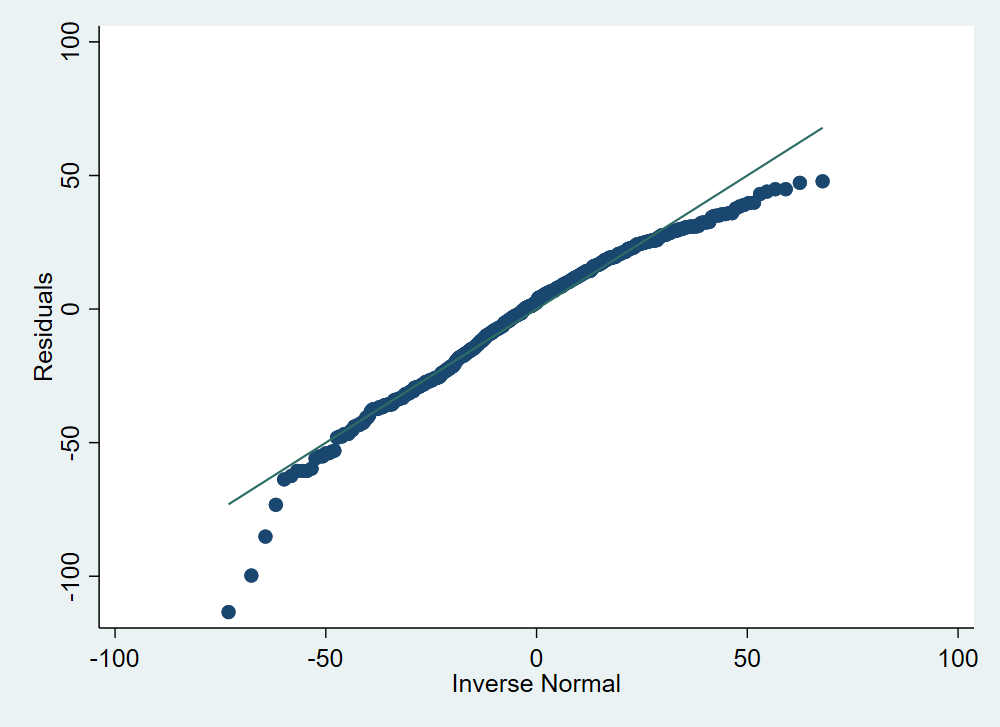

Supplement: Supplementary file 2 — Supplementary Material 2. [file 12891_2024_7312_MOESM2_ESM.zip › saroa-cta-02b-outcome-mixed-rm_abd_bakt-qnorm-12.png]

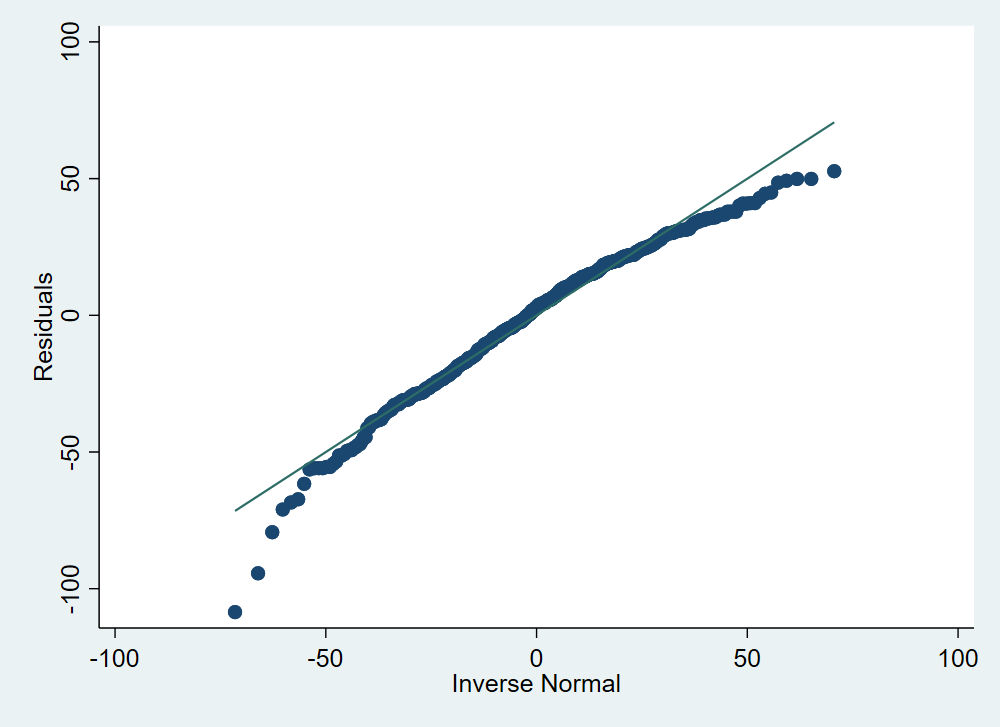

Supplement: Supplementary file 2 — Supplementary Material 2. [file 12891_2024_7312_MOESM2_ESM.zip › saroa-cta-02b-outcome-mixed-rm_abd_bakt-qnorm-24.png]

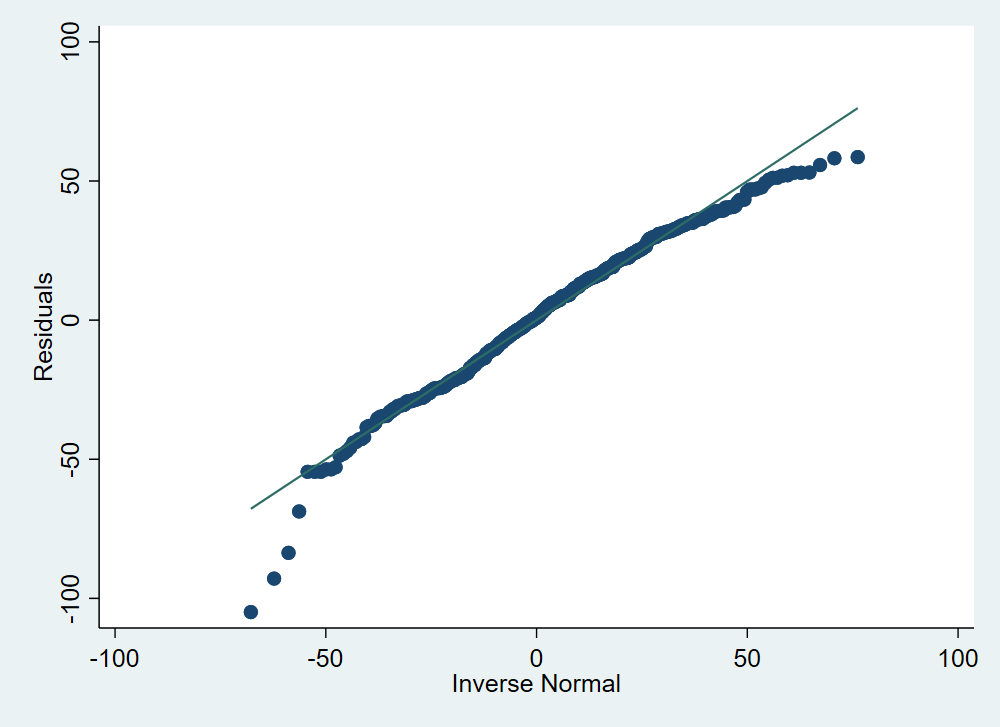

Supplement: Supplementary file 2 — Supplementary Material 2. [file 12891_2024_7312_MOESM2_ESM.zip › saroa-cta-02b-outcome-mixed-rm_abd_bakt-qnorm-6.png]

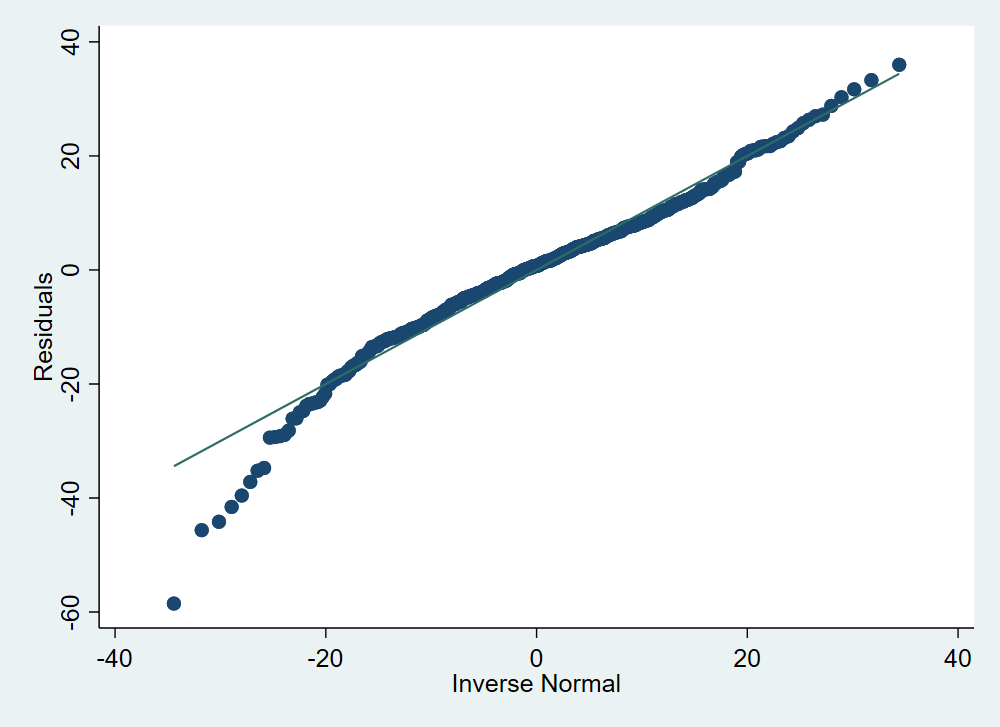

Supplement: Supplementary file 2 — Supplementary Material 2. [file 12891_2024_7312_MOESM2_ESM.zip › saroa-cta-02b-outcome-mixed-rm_abd_bakt-qnorm-mixed.png]

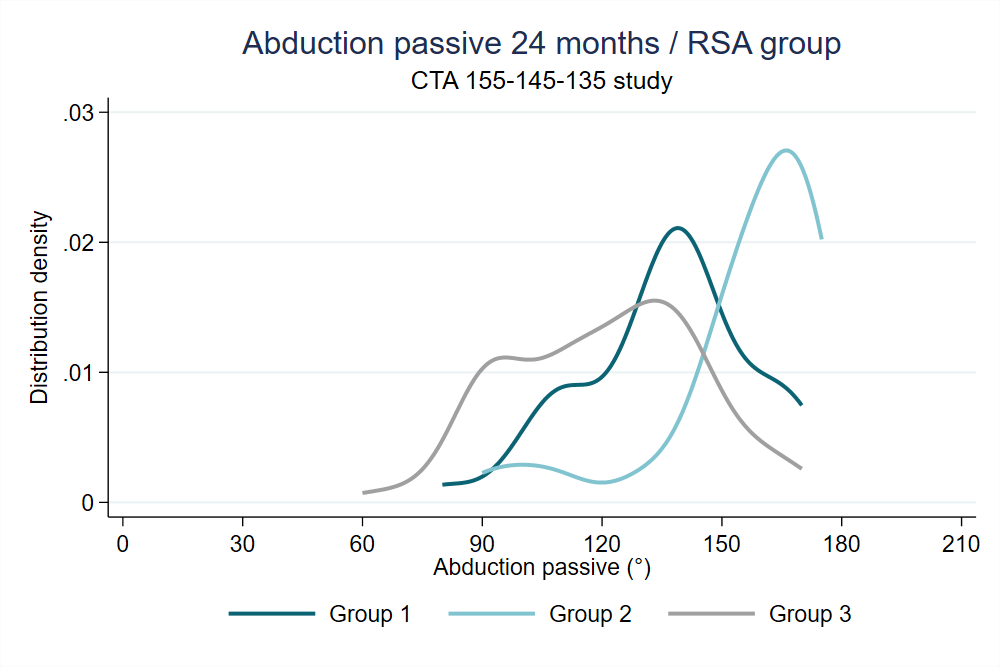

Supplement: Supplementary file 2 — Supplementary Material 2. [file 12891_2024_7312_MOESM2_ESM.zip › saroa-cta-02b-outcome-mixed-rm_abd_bpas-KdensCeiling24mo.png]

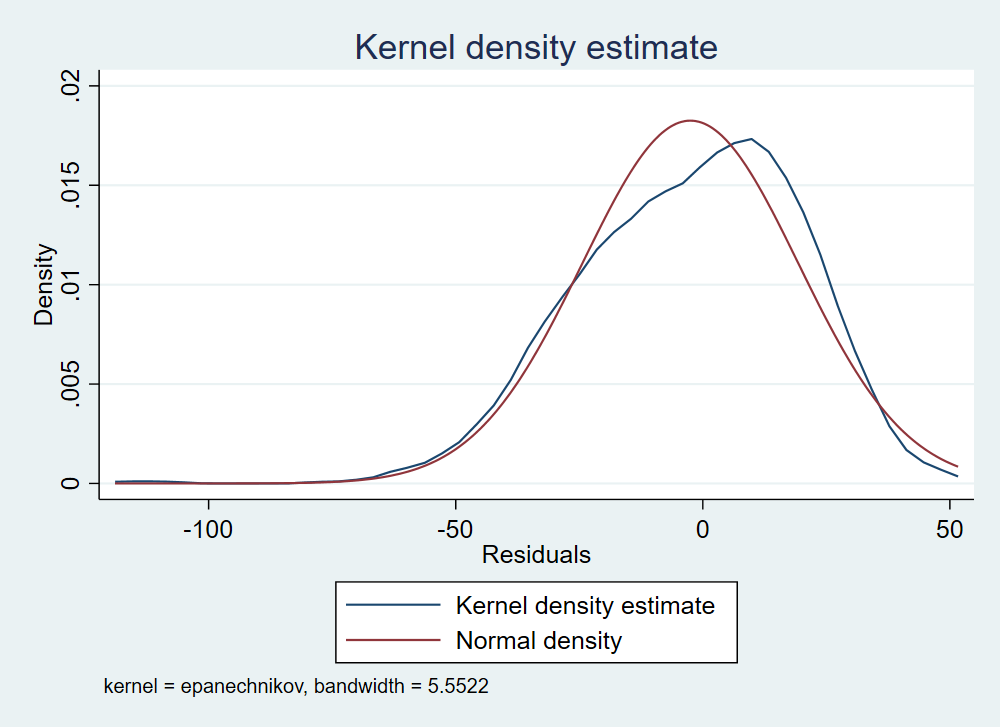

Supplement: Supplementary file 2 — Supplementary Material 2. [file 12891_2024_7312_MOESM2_ESM.zip › saroa-cta-02b-outcome-mixed-rm_abd_bpas-kdensity-12.png]

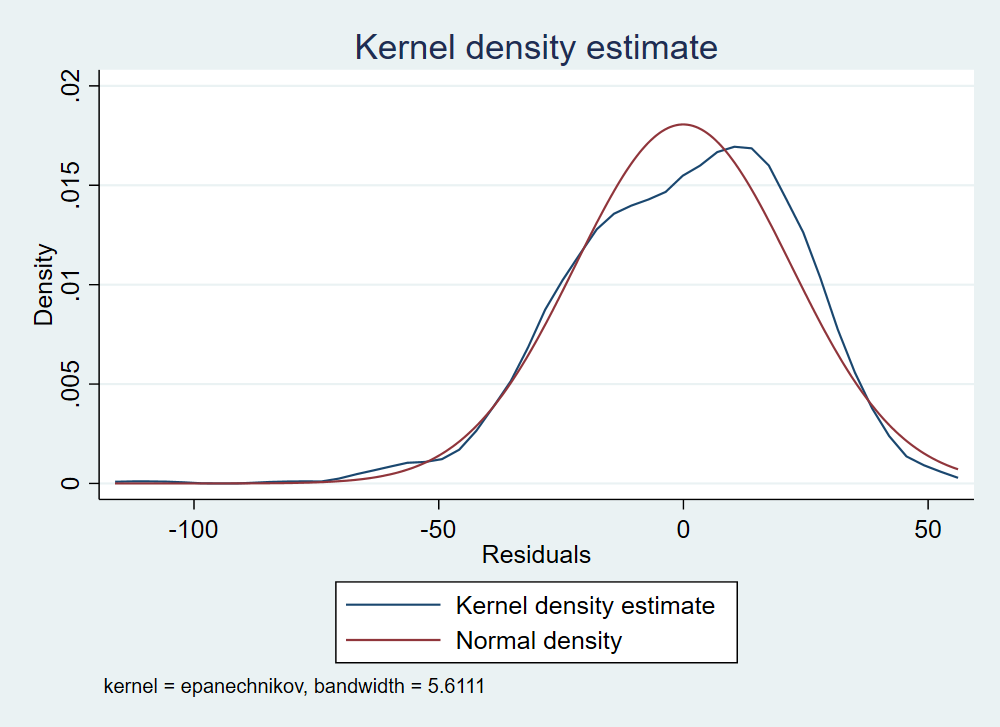

Supplement: Supplementary file 2 — Supplementary Material 2. [file 12891_2024_7312_MOESM2_ESM.zip › saroa-cta-02b-outcome-mixed-rm_abd_bpas-kdensity-24.png]

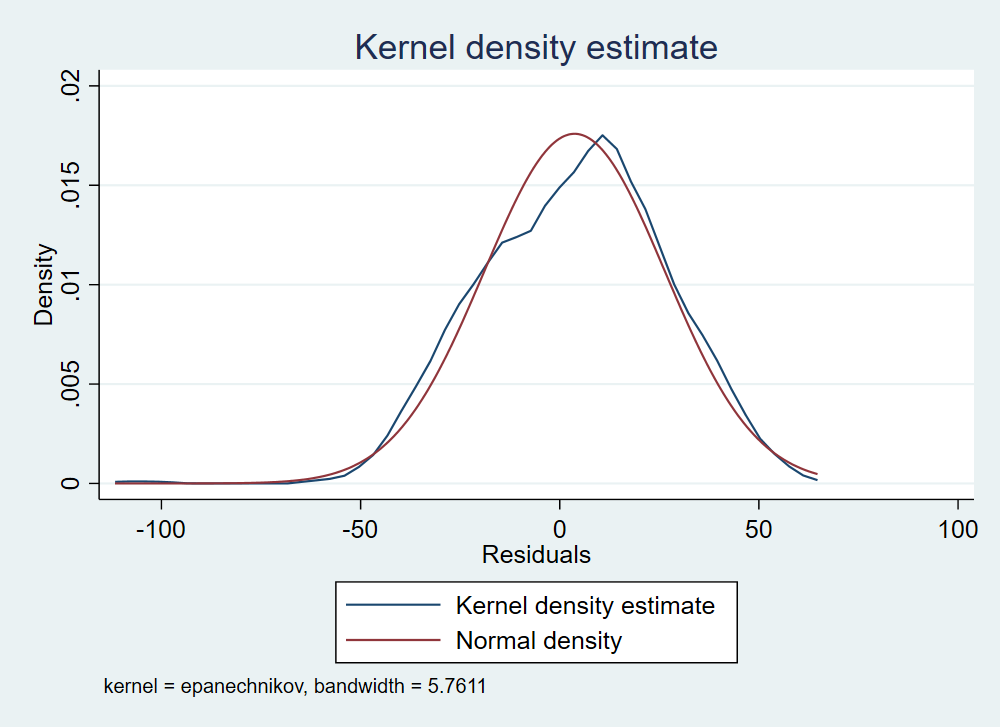

Supplement: Supplementary file 2 — Supplementary Material 2. [file 12891_2024_7312_MOESM2_ESM.zip › saroa-cta-02b-outcome-mixed-rm_abd_bpas-kdensity-6.png]

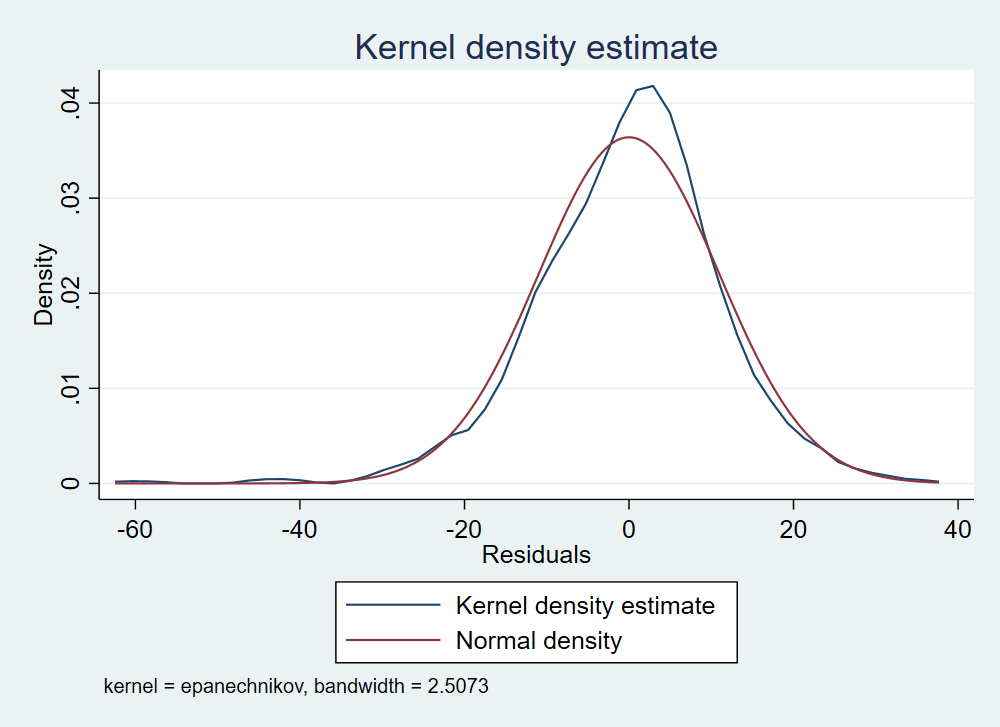

Supplement: Supplementary file 2 — Supplementary Material 2. [file 12891_2024_7312_MOESM2_ESM.zip › saroa-cta-02b-outcome-mixed-rm_abd_bpas-kdensity-mixed.png]

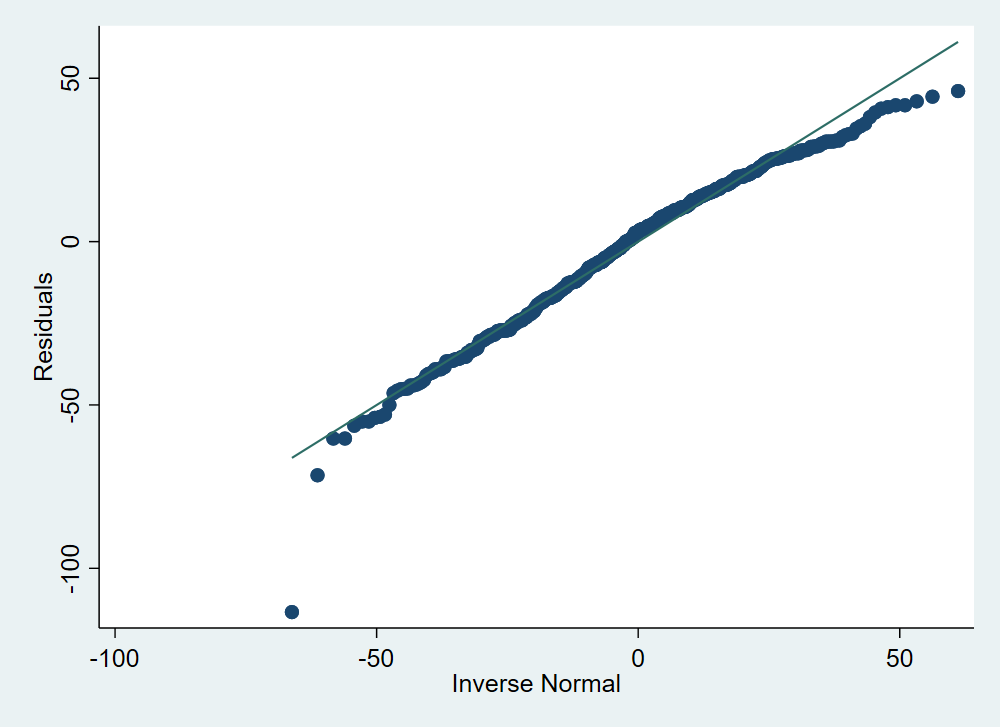

Supplement: Supplementary file 2 — Supplementary Material 2. [file 12891_2024_7312_MOESM2_ESM.zip › saroa-cta-02b-outcome-mixed-rm_abd_bpas-qnorm-12.png]

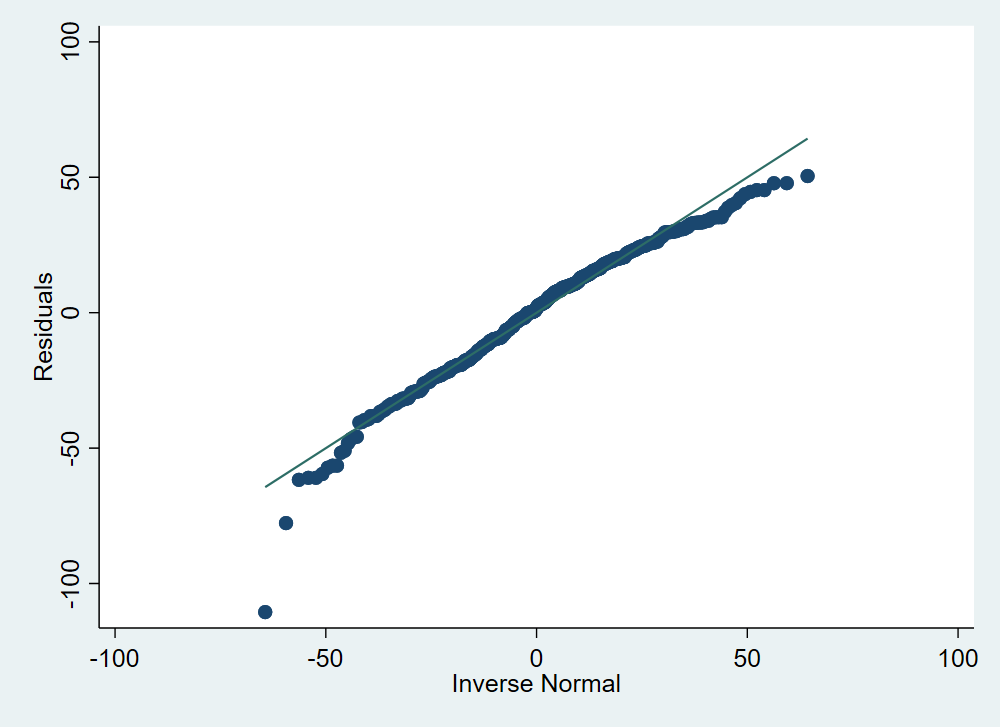

Supplement: Supplementary file 2 — Supplementary Material 2. [file 12891_2024_7312_MOESM2_ESM.zip › saroa-cta-02b-outcome-mixed-rm_abd_bpas-qnorm-24.png]

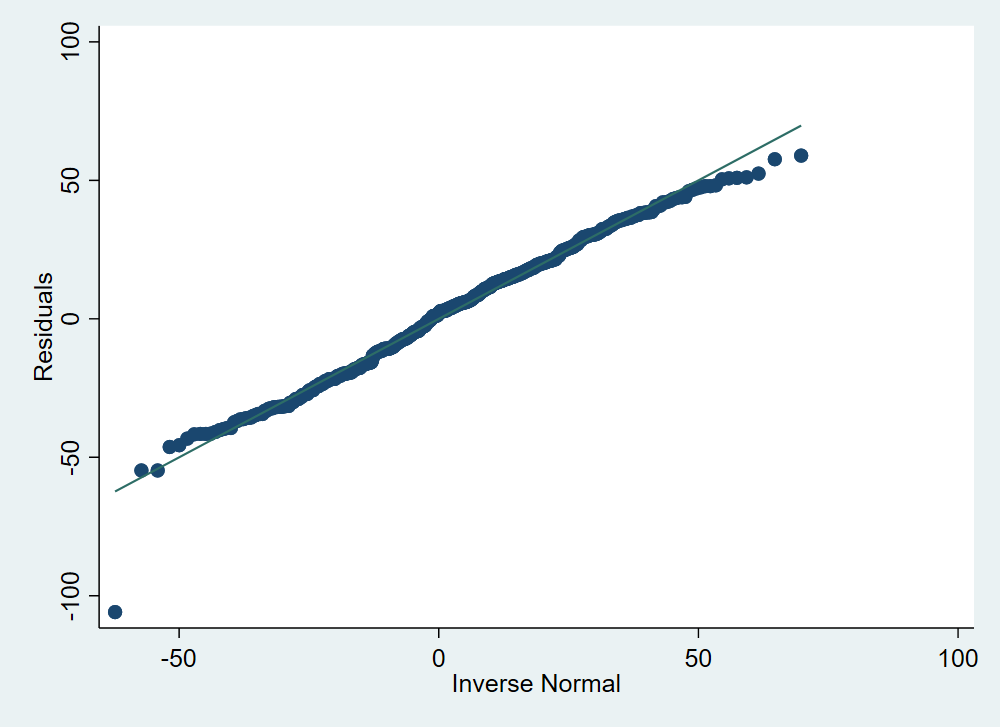

Supplement: Supplementary file 2 — Supplementary Material 2. [file 12891_2024_7312_MOESM2_ESM.zip › saroa-cta-02b-outcome-mixed-rm_abd_bpas-qnorm-6.png]

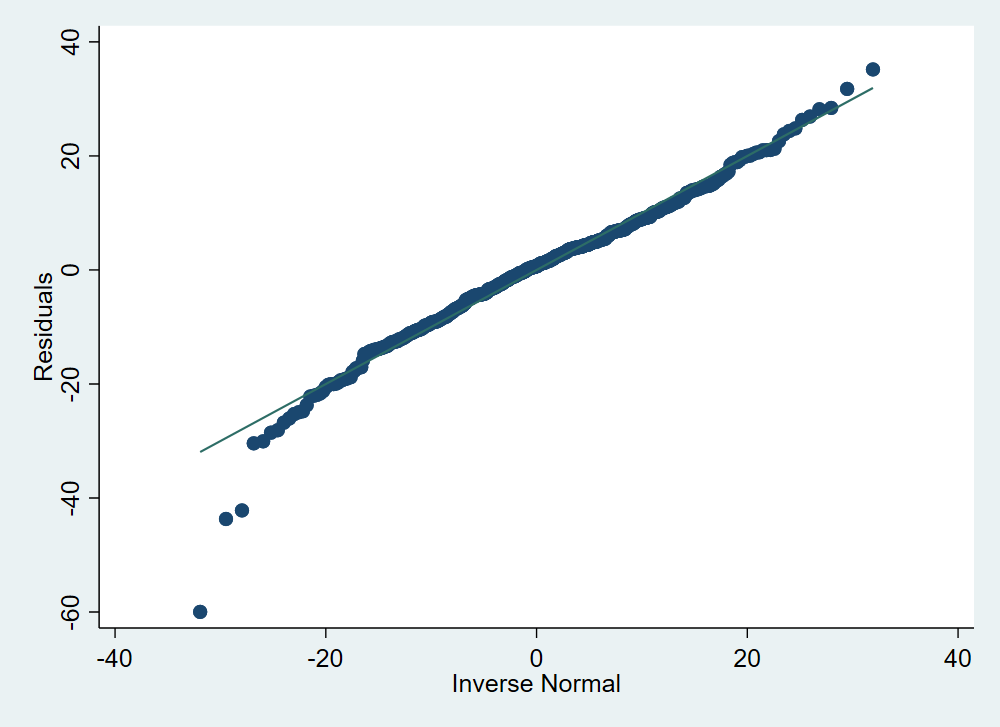

Supplement: Supplementary file 2 — Supplementary Material 2. [file 12891_2024_7312_MOESM2_ESM.zip › saroa-cta-02b-outcome-mixed-rm_abd_bpas-qnorm-mixed.png]

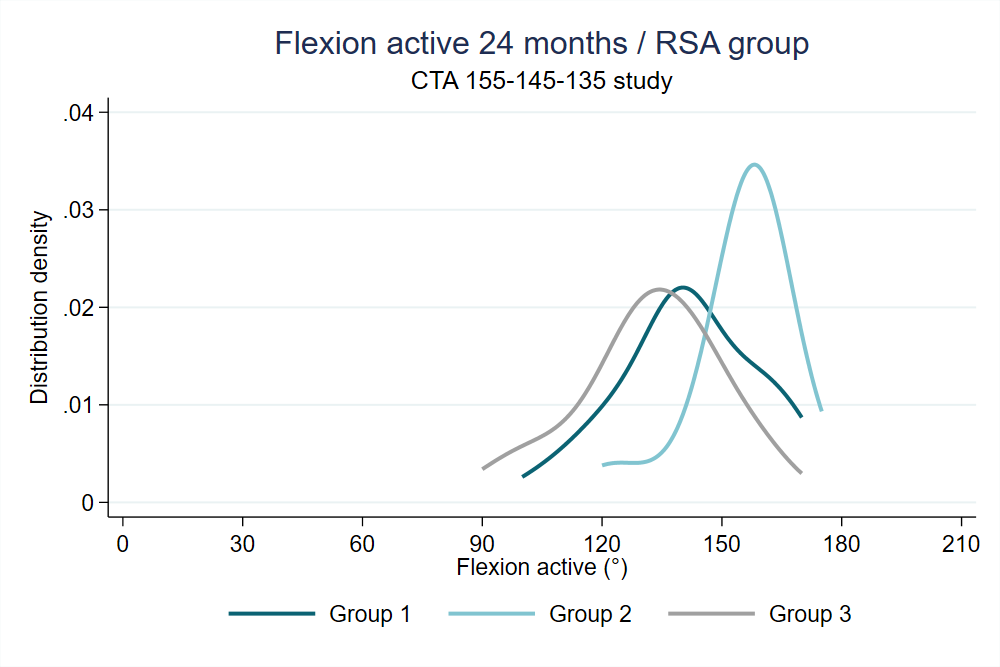

Supplement: Supplementary file 2 — Supplementary Material 2. [file 12891_2024_7312_MOESM2_ESM.zip › saroa-cta-02b-outcome-mixed-rm_ante_bakt-KdensCeiling24mo.png]

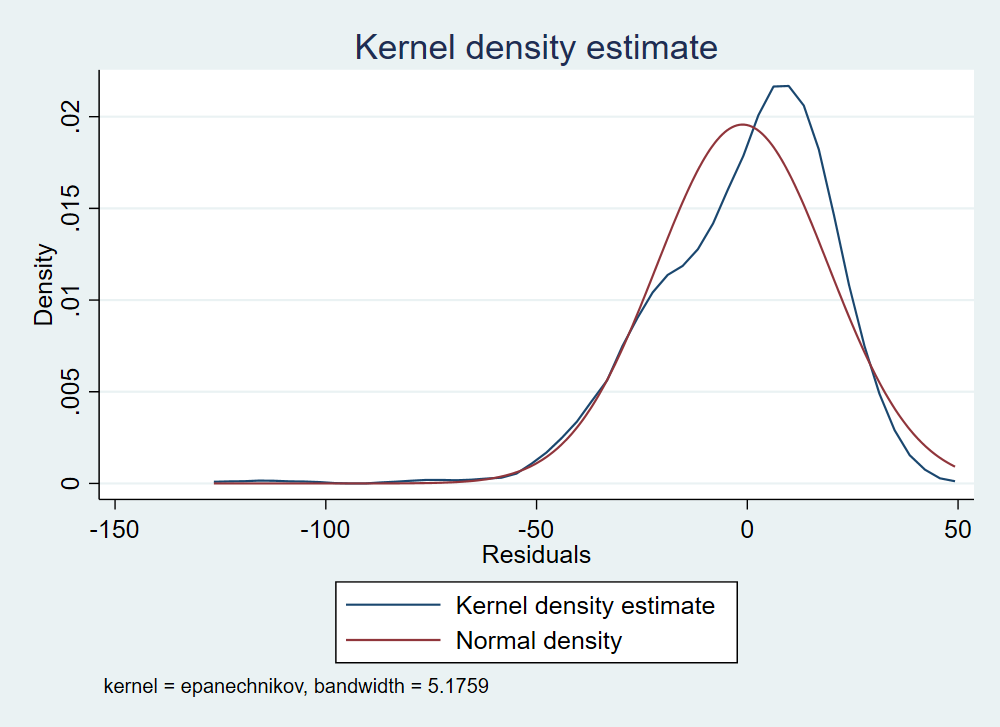

Supplement: Supplementary file 2 — Supplementary Material 2. [file 12891_2024_7312_MOESM2_ESM.zip › saroa-cta-02b-outcome-mixed-rm_ante_bakt-kdensity-12.png]

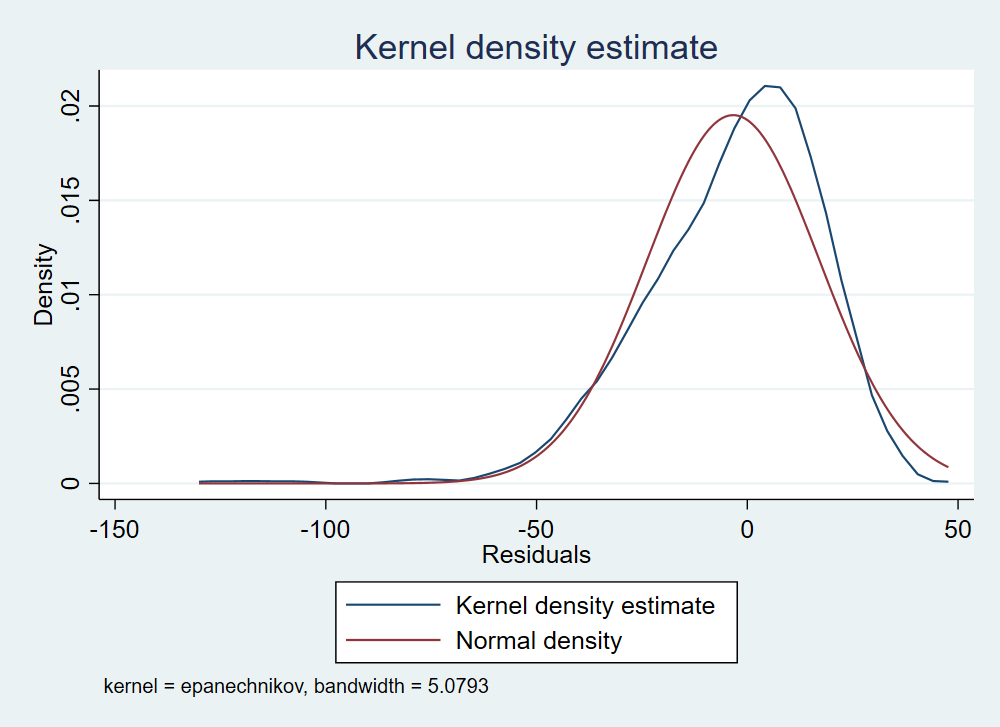

Supplement: Supplementary file 2 — Supplementary Material 2. [file 12891_2024_7312_MOESM2_ESM.zip › saroa-cta-02b-outcome-mixed-rm_ante_bakt-kdensity-24.png]

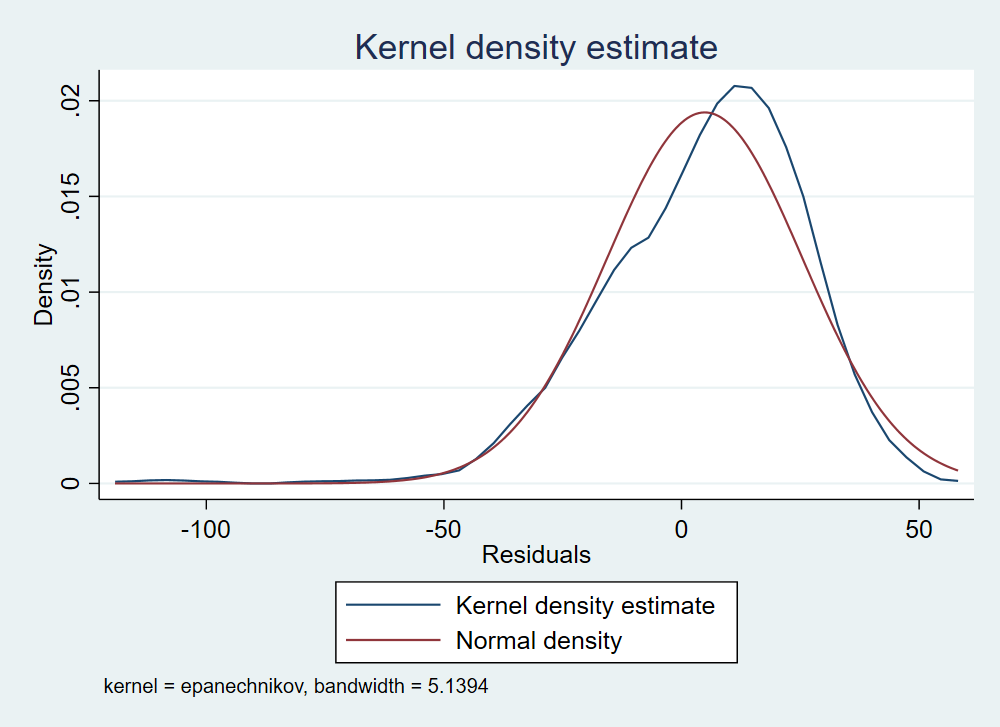

Supplement: Supplementary file 2 — Supplementary Material 2. [file 12891_2024_7312_MOESM2_ESM.zip › saroa-cta-02b-outcome-mixed-rm_ante_bakt-kdensity-6.png]

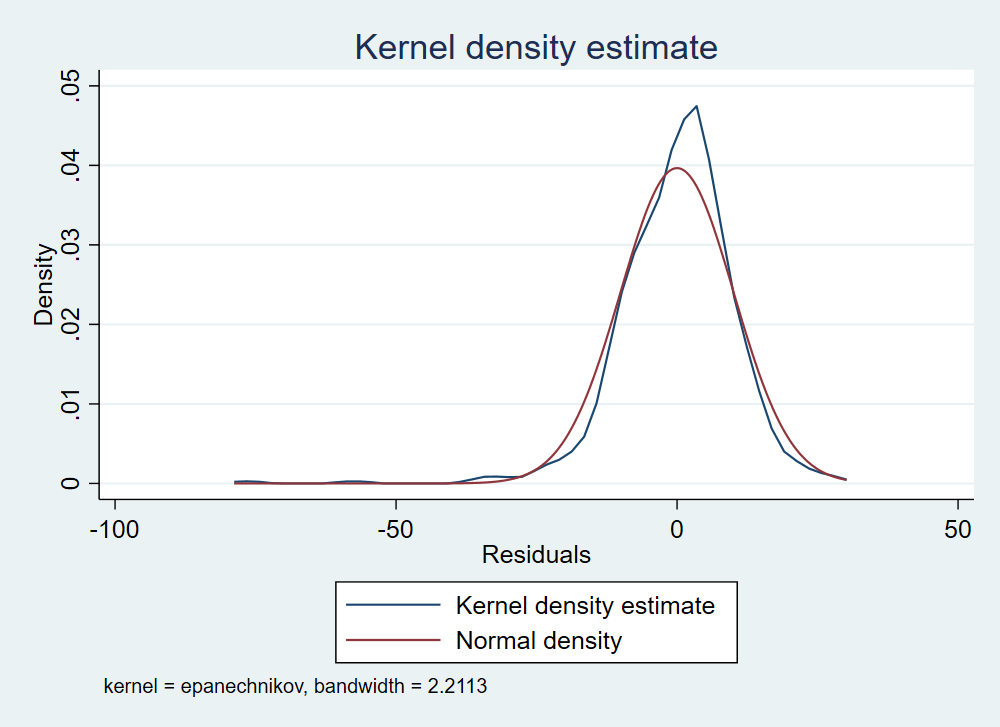

Supplement: Supplementary file 2 — Supplementary Material 2. [file 12891_2024_7312_MOESM2_ESM.zip › saroa-cta-02b-outcome-mixed-rm_ante_bakt-kdensity-mixed.png]

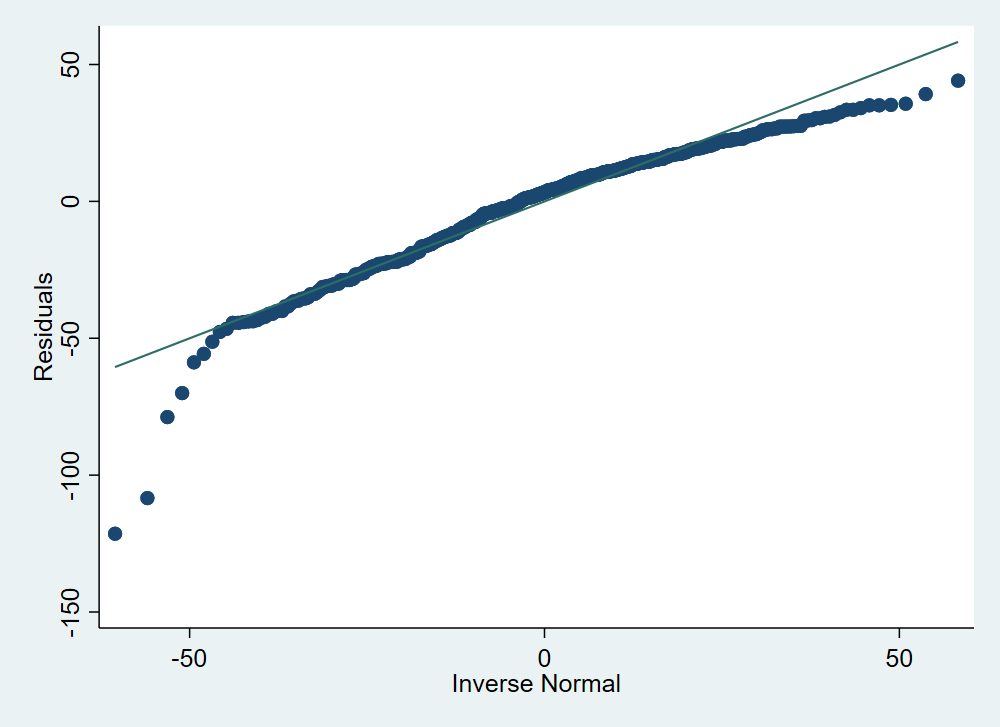

Supplement: Supplementary file 2 — Supplementary Material 2. [file 12891_2024_7312_MOESM2_ESM.zip › saroa-cta-02b-outcome-mixed-rm_ante_bakt-qnorm-12.png]

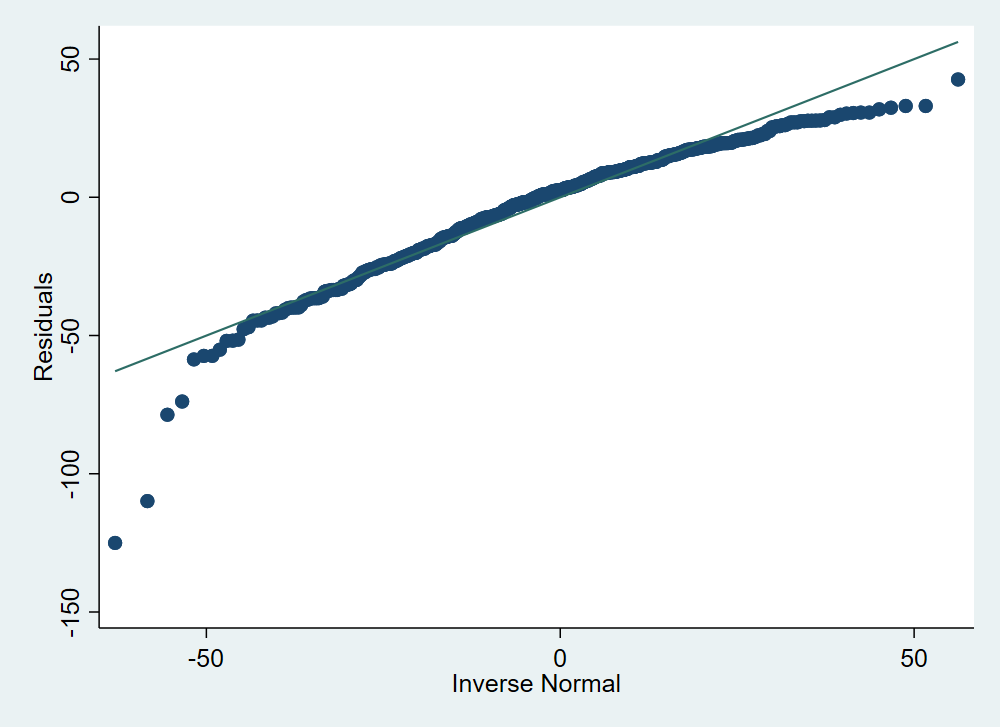

Supplement: Supplementary file 2 — Supplementary Material 2. [file 12891_2024_7312_MOESM2_ESM.zip › saroa-cta-02b-outcome-mixed-rm_ante_bakt-qnorm-24.png]

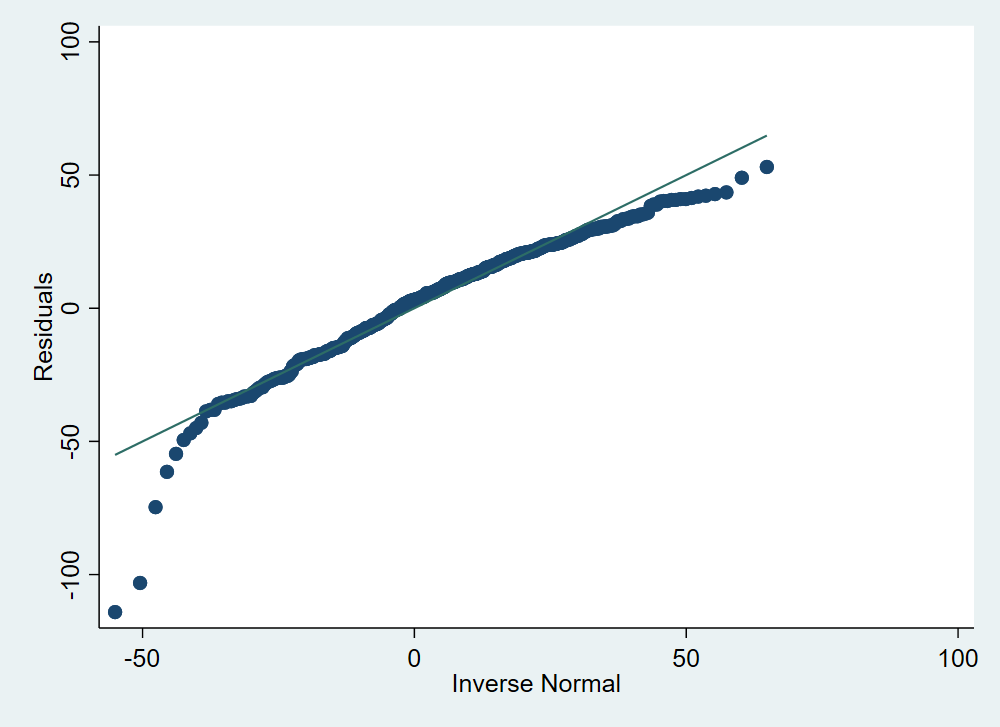

Supplement: Supplementary file 2 — Supplementary Material 2. [file 12891_2024_7312_MOESM2_ESM.zip › saroa-cta-02b-outcome-mixed-rm_ante_bakt-qnorm-6.png]

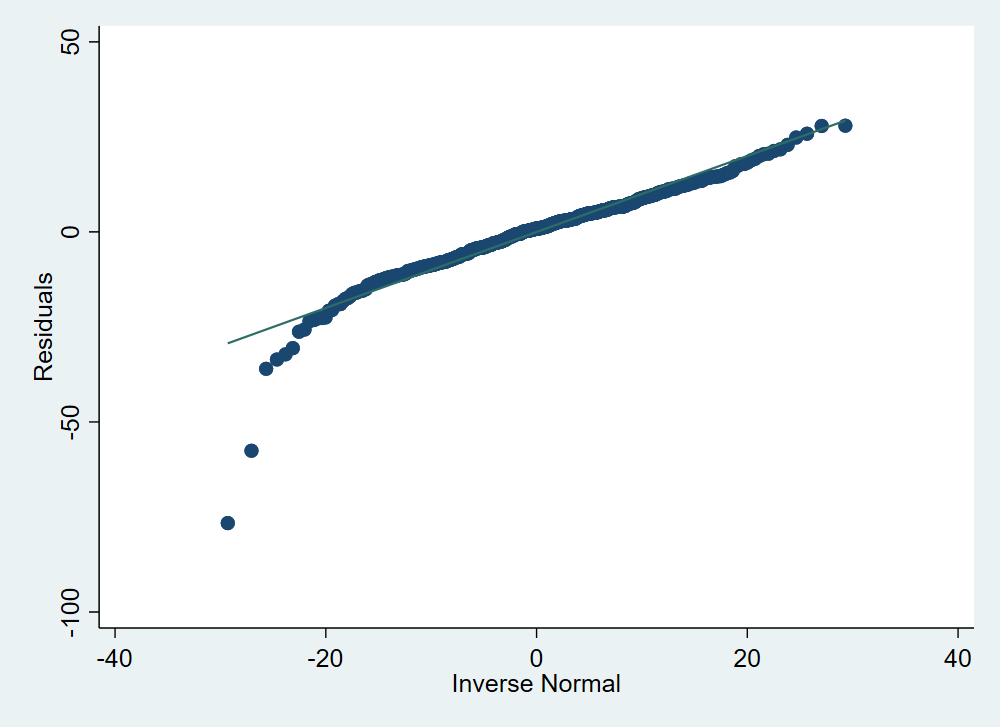

Supplement: Supplementary file 2 — Supplementary Material 2. [file 12891_2024_7312_MOESM2_ESM.zip › saroa-cta-02b-outcome-mixed-rm_ante_bakt-qnorm-mixed.png]

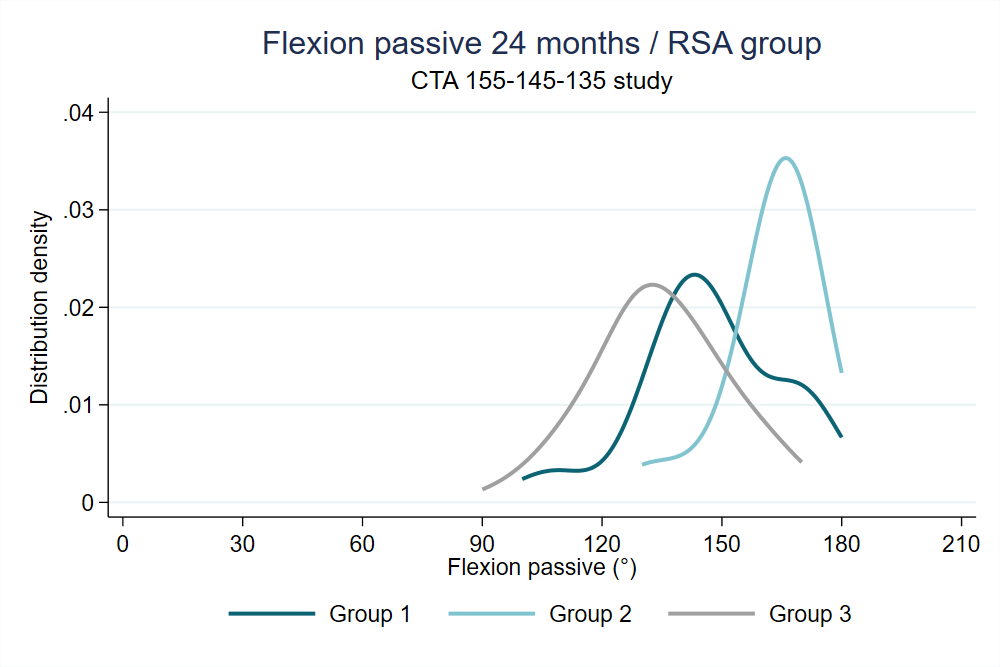

Supplement: Supplementary file 2 — Supplementary Material 2. [file 12891_2024_7312_MOESM2_ESM.zip › saroa-cta-02b-outcome-mixed-rm_ante_bpas-KdensCeiling24mo.png]

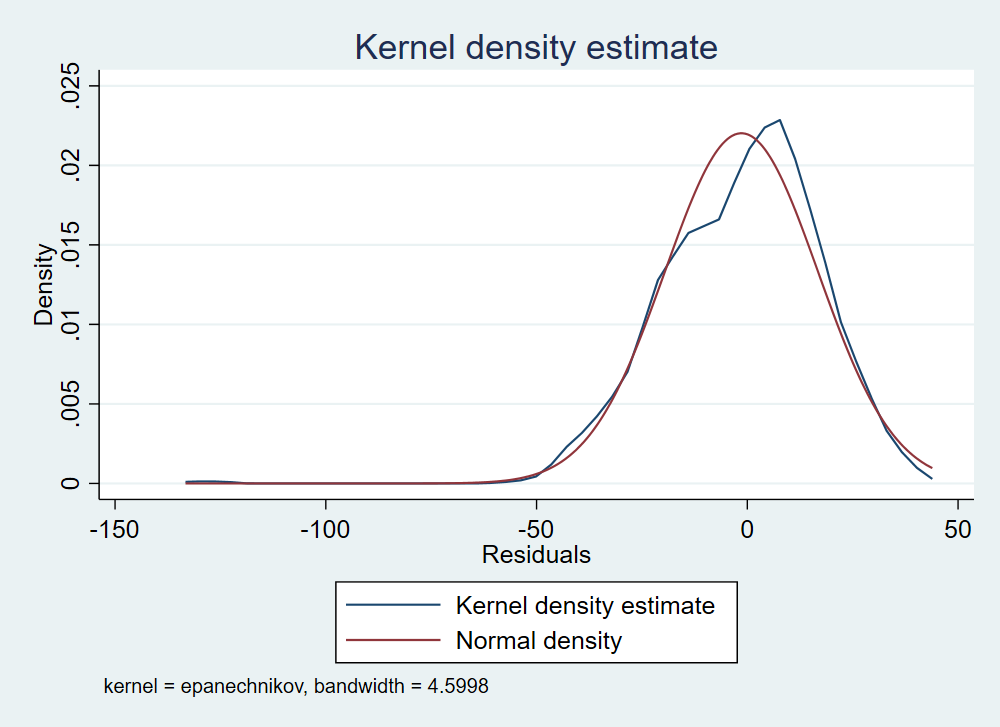

Supplement: Supplementary file 2 — Supplementary Material 2. [file 12891_2024_7312_MOESM2_ESM.zip › saroa-cta-02b-outcome-mixed-rm_ante_bpas-kdensity-12.png]

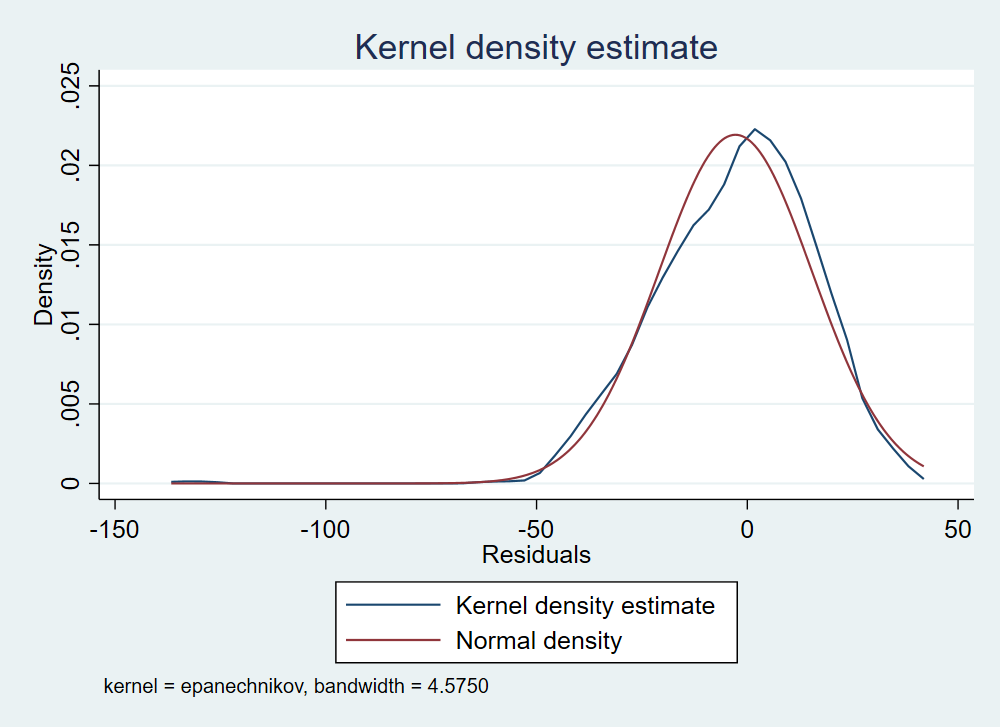

Supplement: Supplementary file 2 — Supplementary Material 2. [file 12891_2024_7312_MOESM2_ESM.zip › saroa-cta-02b-outcome-mixed-rm_ante_bpas-kdensity-24.png]

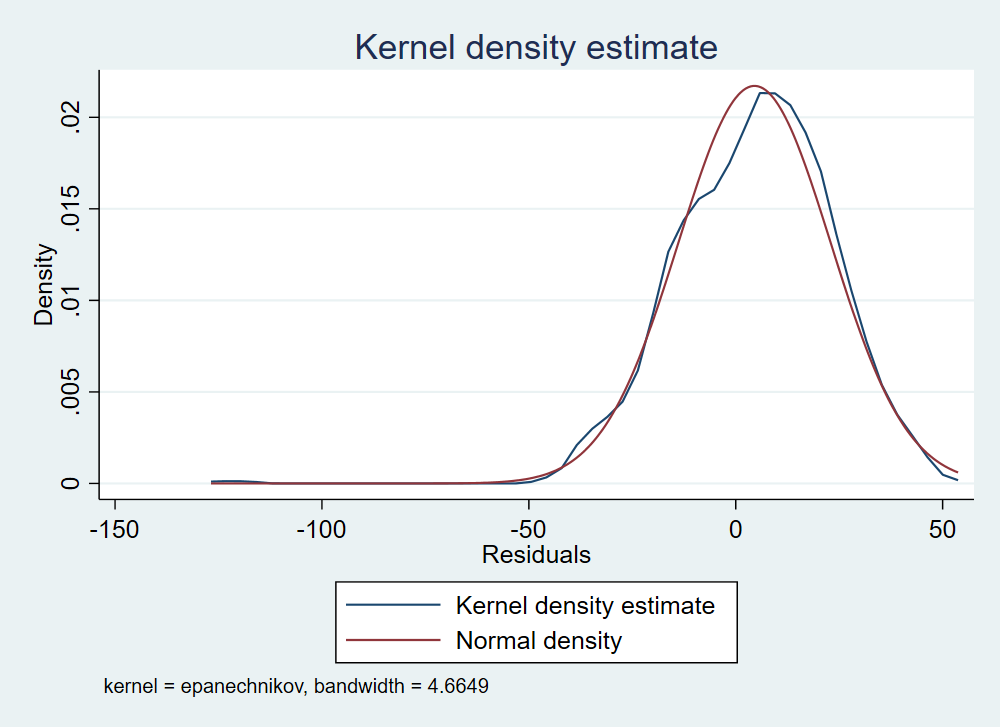

Supplement: Supplementary file 2 — Supplementary Material 2. [file 12891_2024_7312_MOESM2_ESM.zip › saroa-cta-02b-outcome-mixed-rm_ante_bpas-kdensity-6.png]

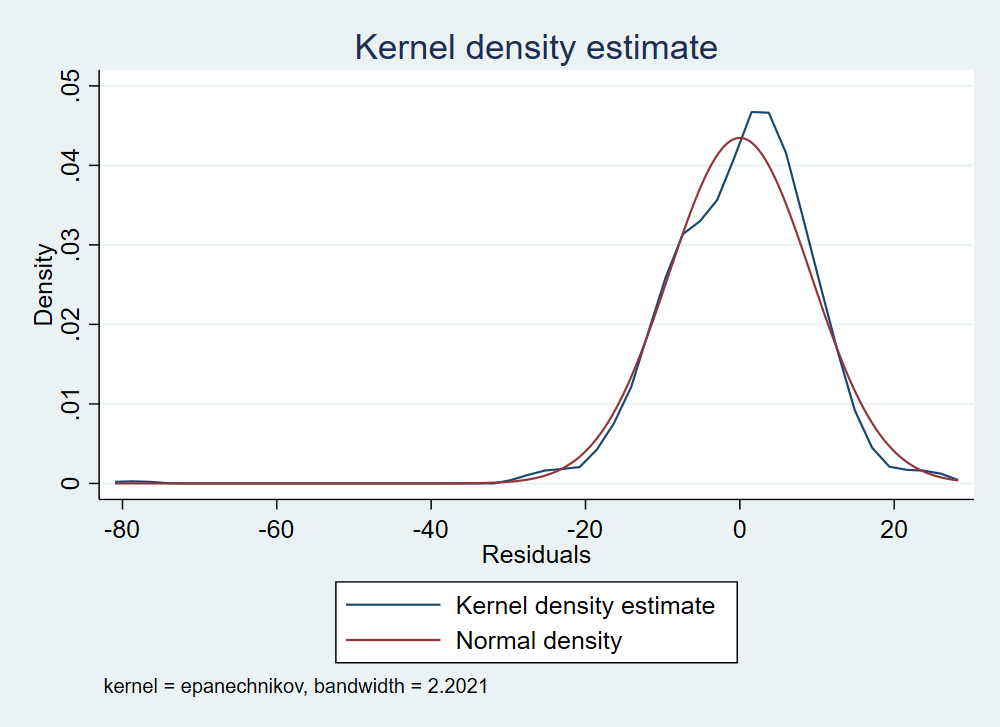

Supplement: Supplementary file 2 — Supplementary Material 2. [file 12891_2024_7312_MOESM2_ESM.zip › saroa-cta-02b-outcome-mixed-rm_ante_bpas-kdensity-mixed.png]

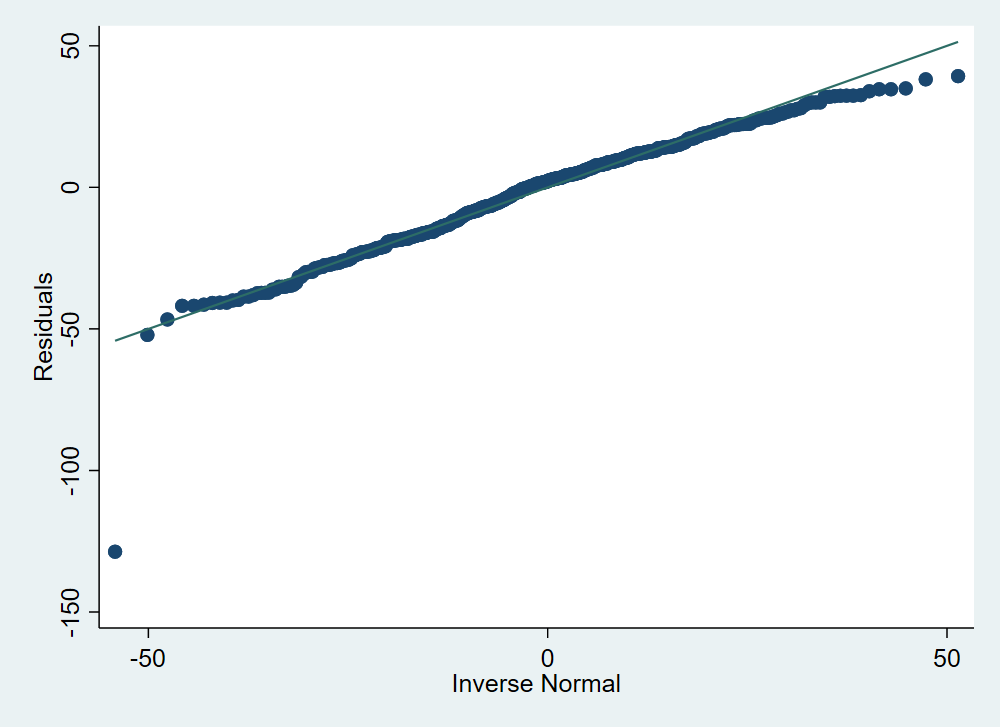

Supplement: Supplementary file 2 — Supplementary Material 2. [file 12891_2024_7312_MOESM2_ESM.zip › saroa-cta-02b-outcome-mixed-rm_ante_bpas-qnorm-12.png]

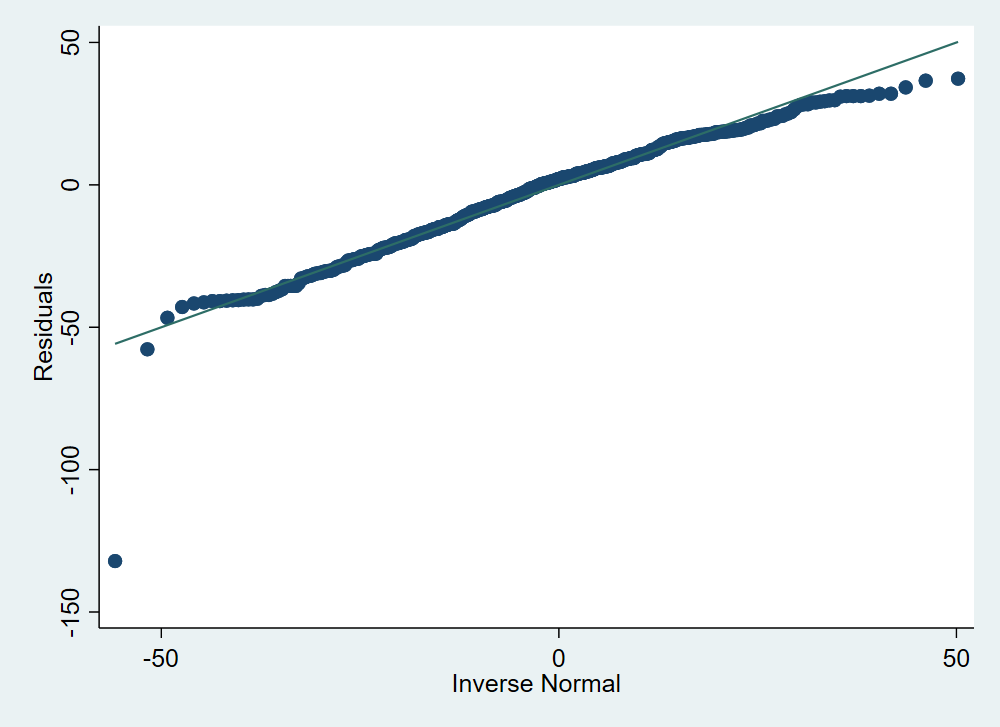

Supplement: Supplementary file 2 — Supplementary Material 2. [file 12891_2024_7312_MOESM2_ESM.zip › saroa-cta-02b-outcome-mixed-rm_ante_bpas-qnorm-24.png]

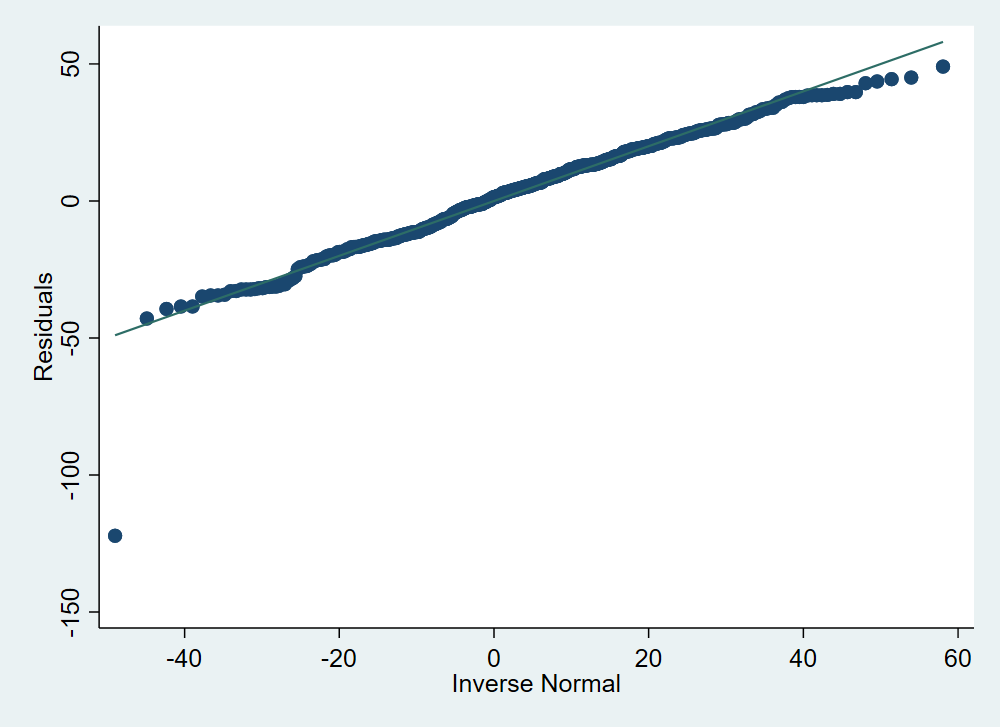

Supplement: Supplementary file 2 — Supplementary Material 2. [file 12891_2024_7312_MOESM2_ESM.zip › saroa-cta-02b-outcome-mixed-rm_ante_bpas-qnorm-6.png]

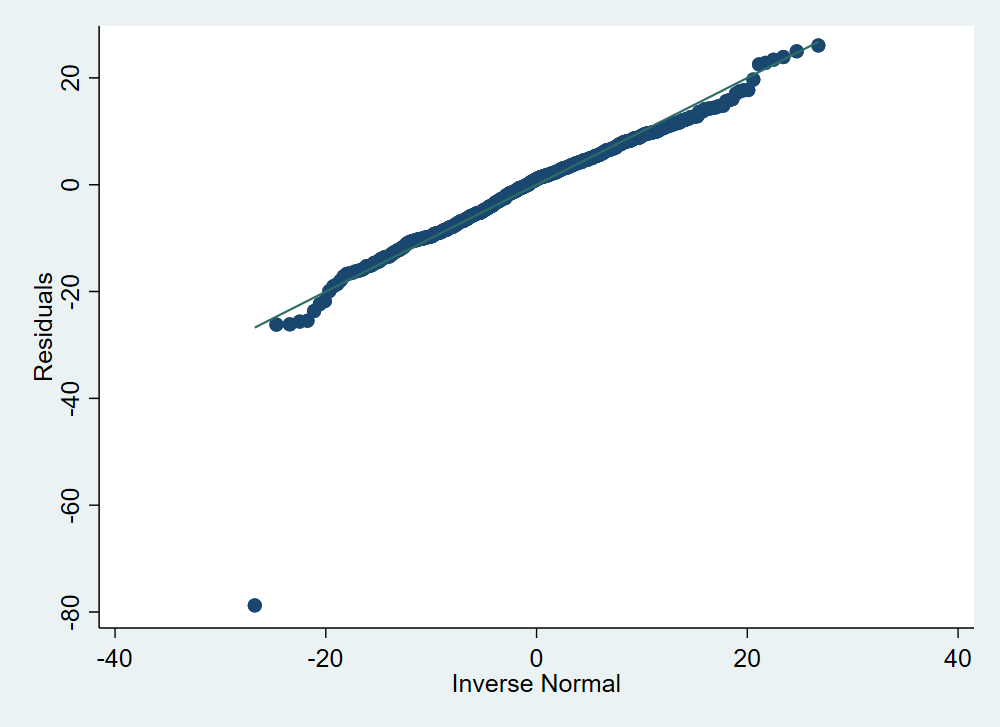

Supplement: Supplementary file 2 — Supplementary Material 2. [file 12891_2024_7312_MOESM2_ESM.zip › saroa-cta-02b-outcome-mixed-rm_ante_bpas-qnorm-mixed.png]

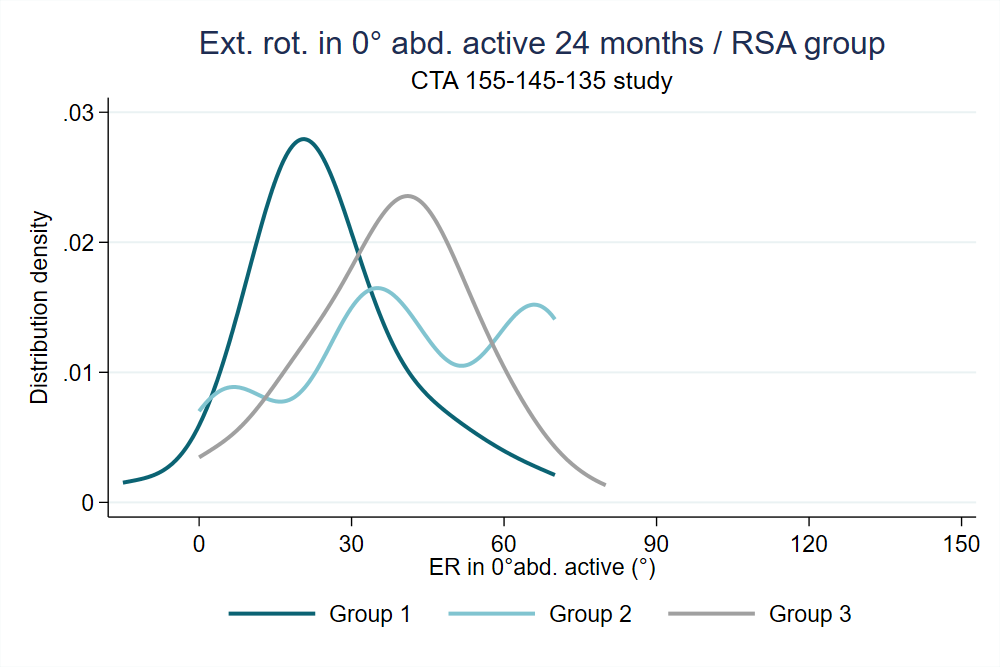

Supplement: Supplementary file 2 — Supplementary Material 2. [file 12891_2024_7312_MOESM2_ESM.zip › saroa-cta-02b-outcome-mixed-rm_ar0_bakt-KdensCeiling24mo.png]

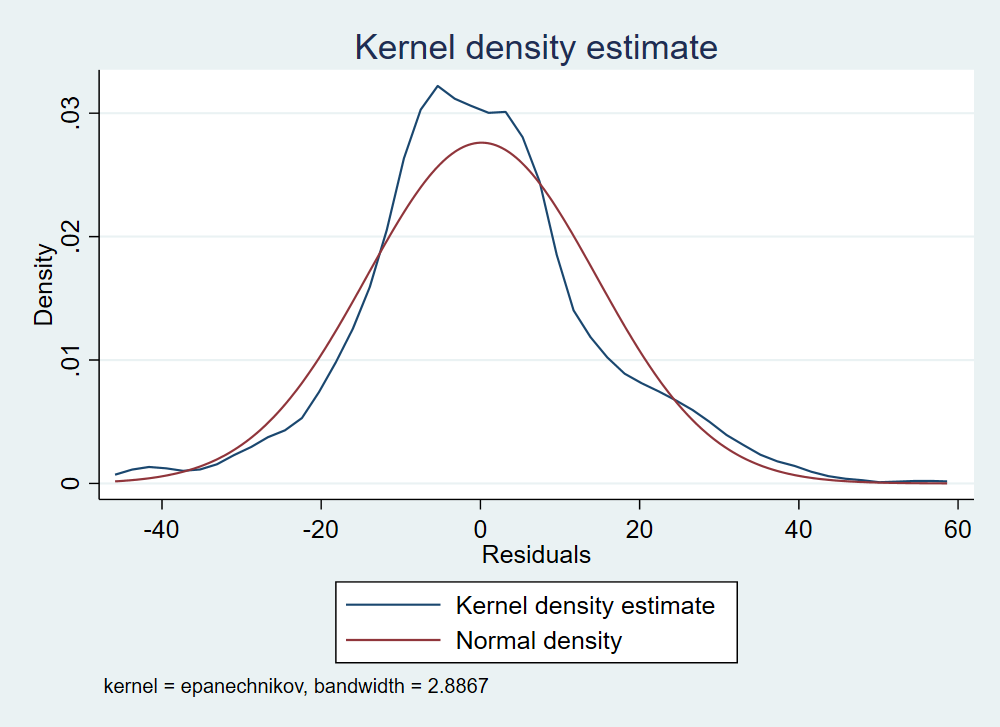

Supplement: Supplementary file 2 — Supplementary Material 2. [file 12891_2024_7312_MOESM2_ESM.zip › saroa-cta-02b-outcome-mixed-rm_ar0_bakt-kdensity-12.png]

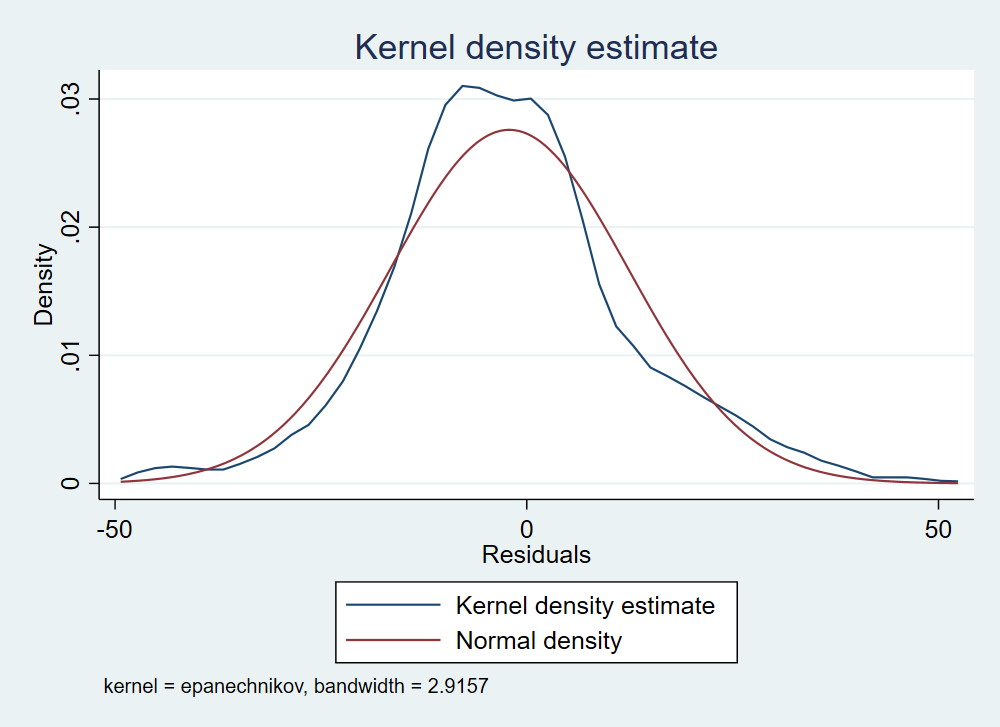

Supplement: Supplementary file 2 — Supplementary Material 2. [file 12891_2024_7312_MOESM2_ESM.zip › saroa-cta-02b-outcome-mixed-rm_ar0_bakt-kdensity-24.png]

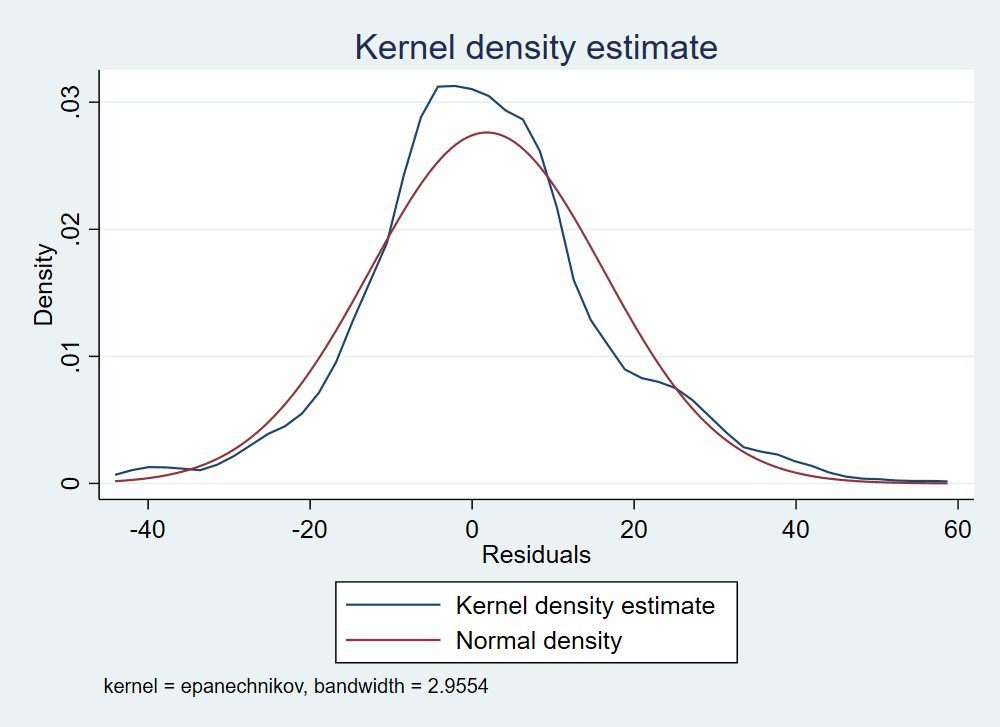

Supplement: Supplementary file 2 — Supplementary Material 2. [file 12891_2024_7312_MOESM2_ESM.zip › saroa-cta-02b-outcome-mixed-rm_ar0_bakt-kdensity-6.png]

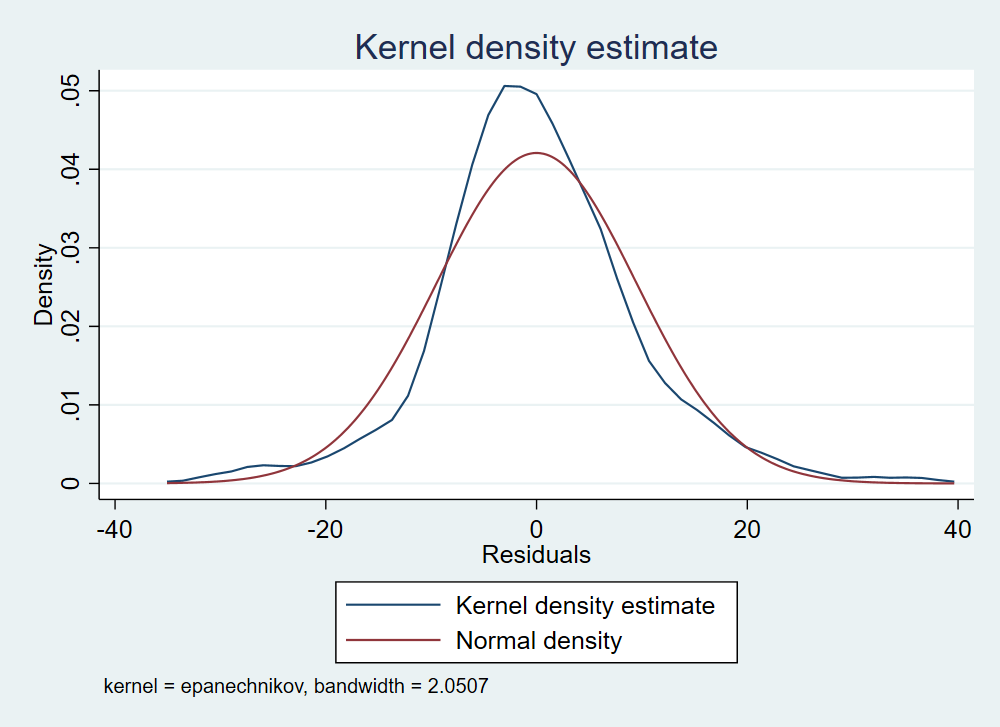

Supplement: Supplementary file 2 — Supplementary Material 2. [file 12891_2024_7312_MOESM2_ESM.zip › saroa-cta-02b-outcome-mixed-rm_ar0_bakt-kdensity-mixed.png]

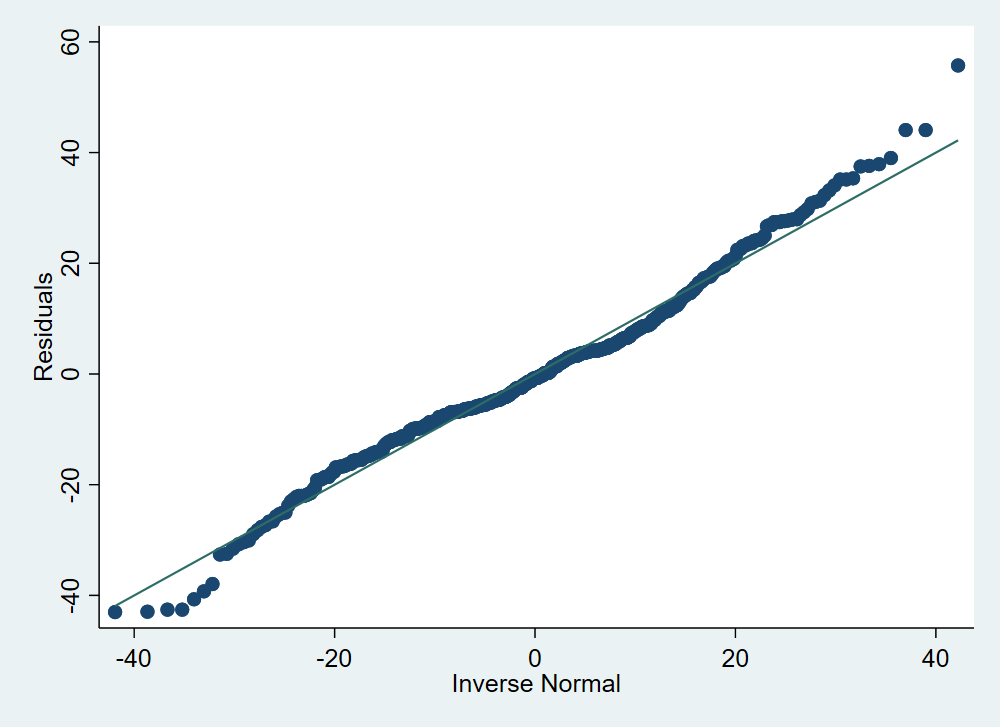

Supplement: Supplementary file 2 — Supplementary Material 2. [file 12891_2024_7312_MOESM2_ESM.zip › saroa-cta-02b-outcome-mixed-rm_ar0_bakt-qnorm-12.png]

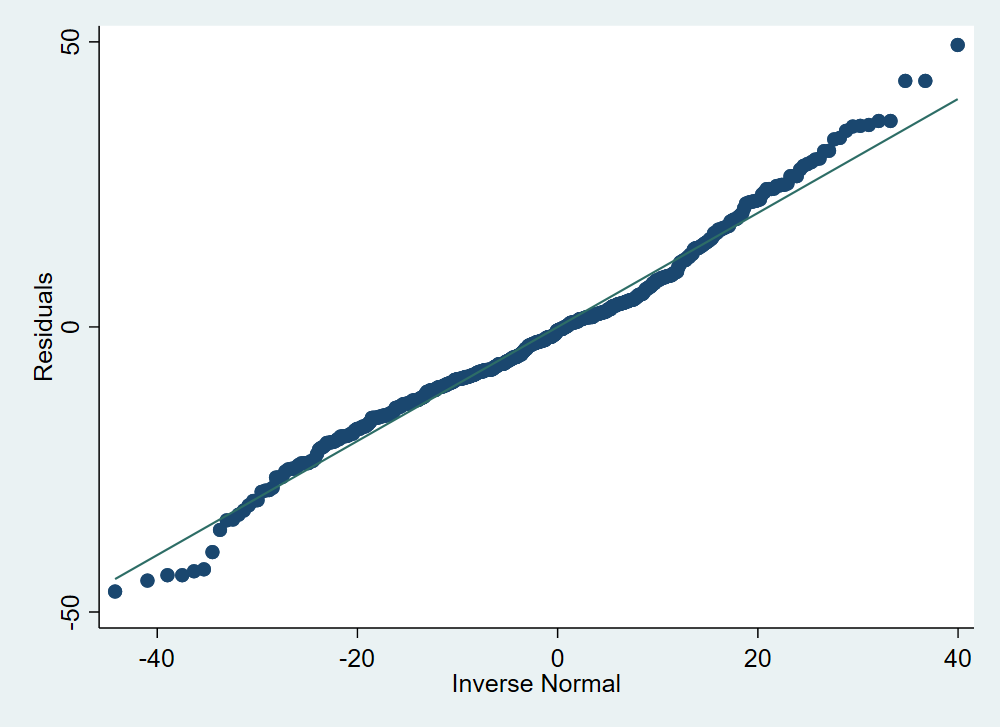

Supplement: Supplementary file 2 — Supplementary Material 2. [file 12891_2024_7312_MOESM2_ESM.zip › saroa-cta-02b-outcome-mixed-rm_ar0_bakt-qnorm-24.png]

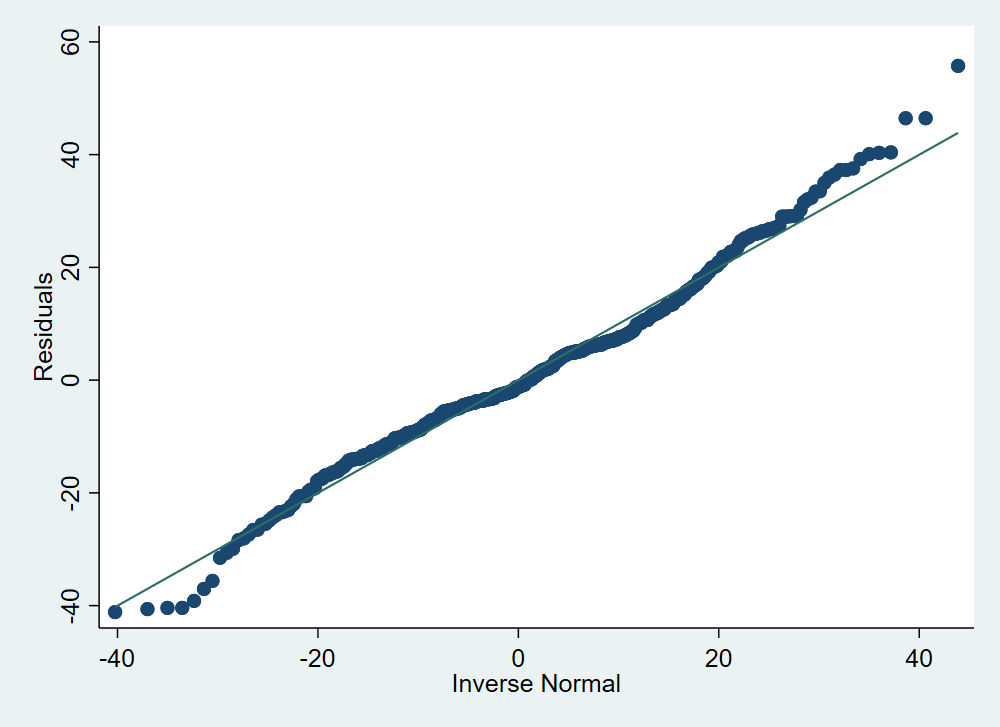

Supplement: Supplementary file 2 — Supplementary Material 2. [file 12891_2024_7312_MOESM2_ESM.zip › saroa-cta-02b-outcome-mixed-rm_ar0_bakt-qnorm-6.png]

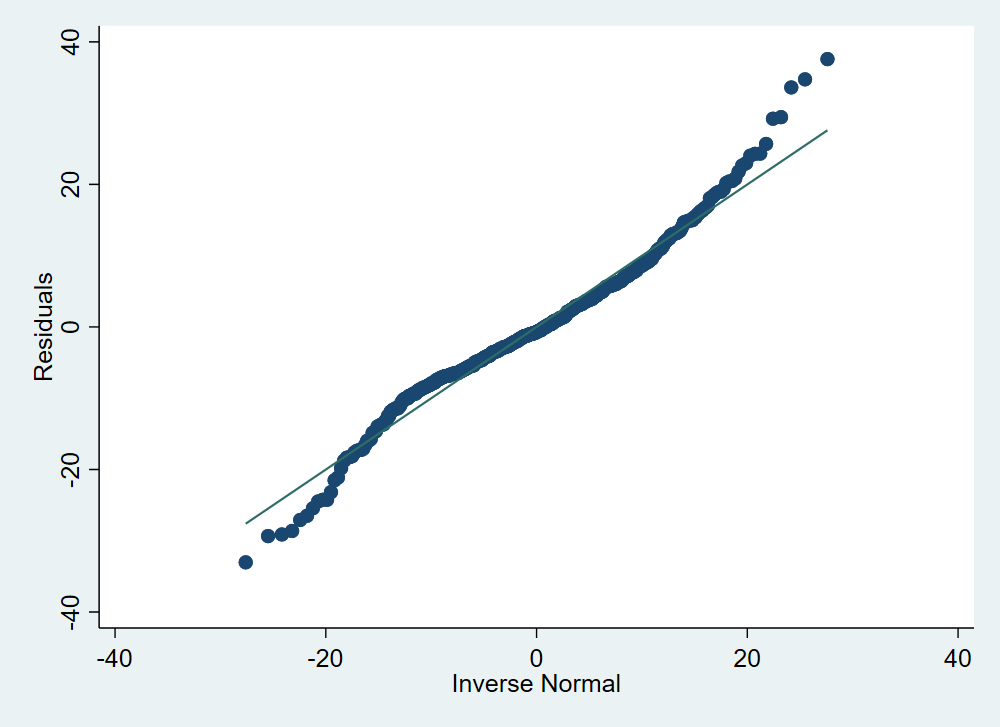

Supplement: Supplementary file 2 — Supplementary Material 2. [file 12891_2024_7312_MOESM2_ESM.zip › saroa-cta-02b-outcome-mixed-rm_ar0_bakt-qnorm-mixed.png]

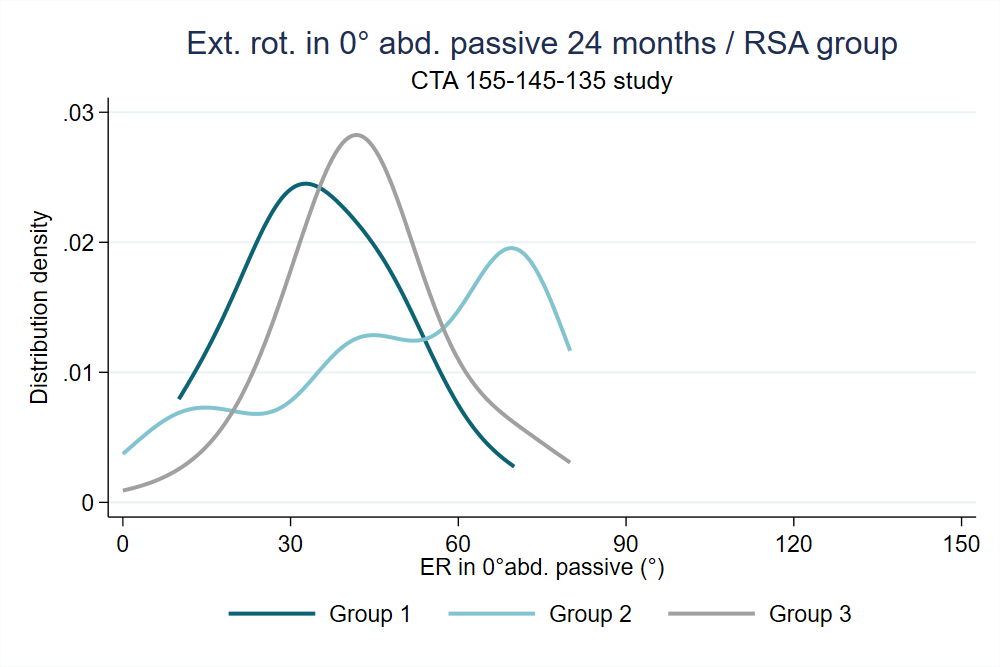

Supplement: Supplementary file 2 — Supplementary Material 2. [file 12891_2024_7312_MOESM2_ESM.zip › saroa-cta-02b-outcome-mixed-rm_ar0_bpas-KdensCeiling24mo.png]

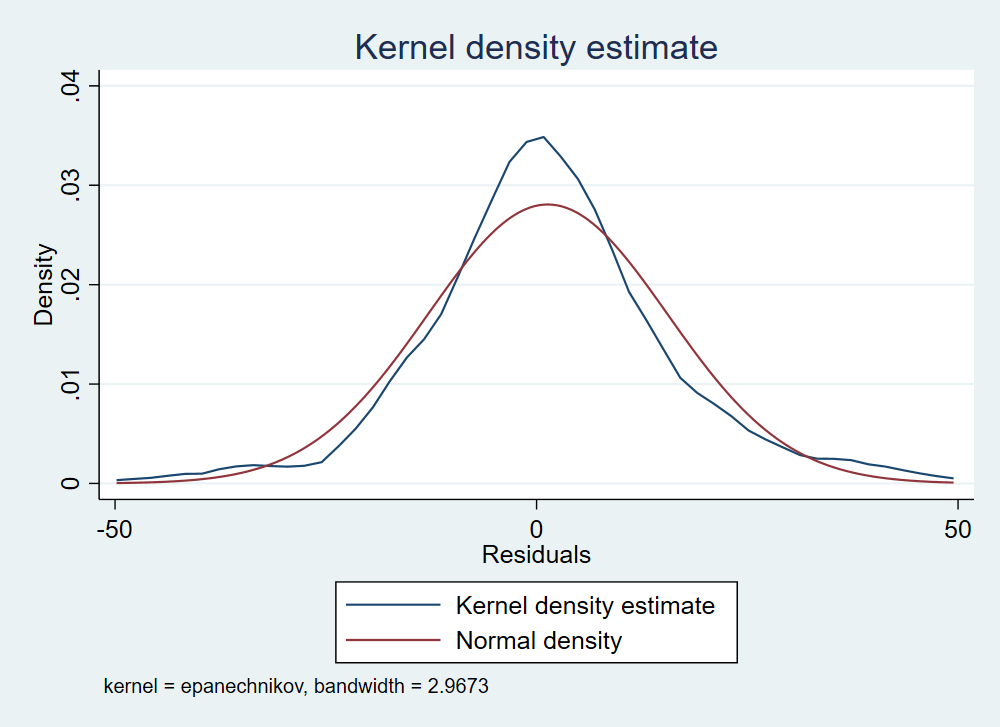

Supplement: Supplementary file 2 — Supplementary Material 2. [file 12891_2024_7312_MOESM2_ESM.zip › saroa-cta-02b-outcome-mixed-rm_ar0_bpas-kdensity-12.png]

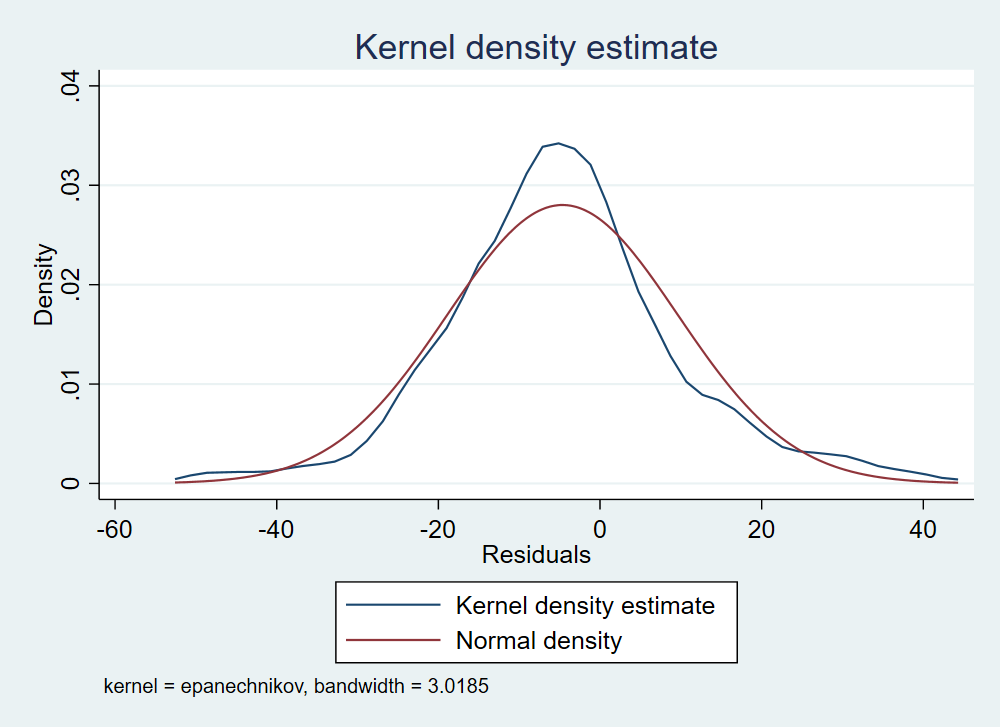

Supplement: Supplementary file 2 — Supplementary Material 2. [file 12891_2024_7312_MOESM2_ESM.zip › saroa-cta-02b-outcome-mixed-rm_ar0_bpas-kdensity-24.png]

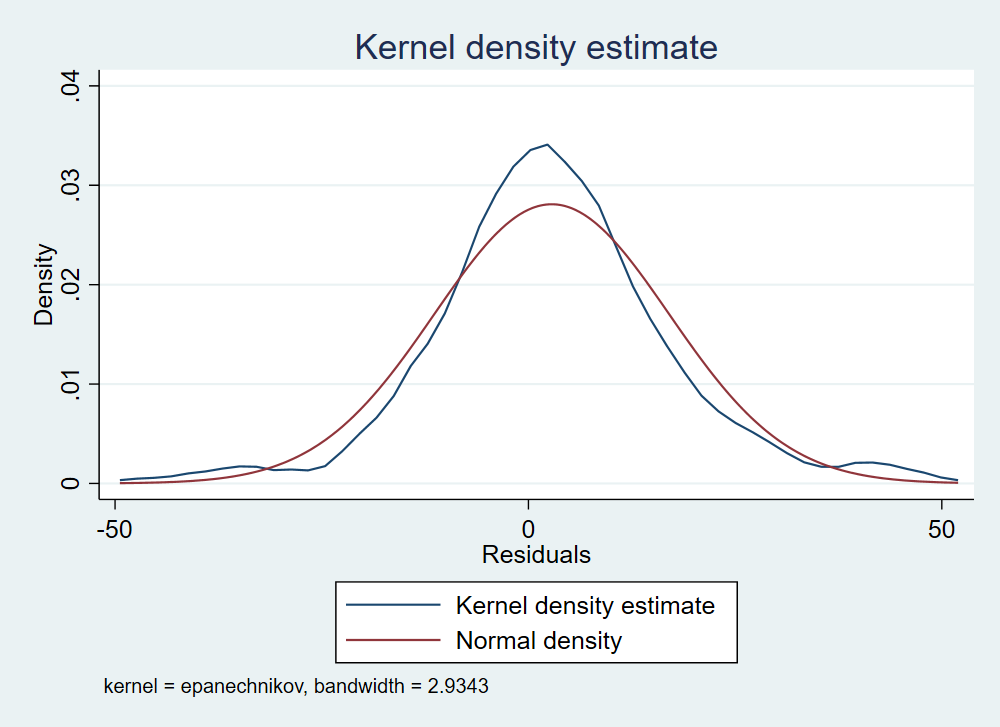

Supplement: Supplementary file 2 — Supplementary Material 2. [file 12891_2024_7312_MOESM2_ESM.zip › saroa-cta-02b-outcome-mixed-rm_ar0_bpas-kdensity-6.png]

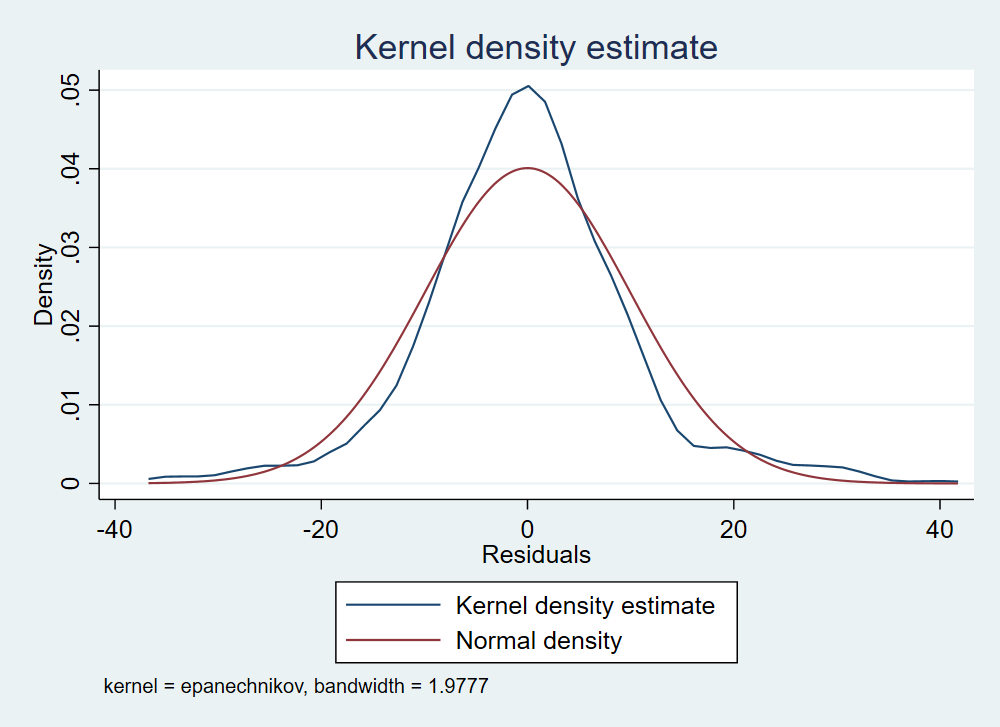

Supplement: Supplementary file 2 — Supplementary Material 2. [file 12891_2024_7312_MOESM2_ESM.zip › saroa-cta-02b-outcome-mixed-rm_ar0_bpas-kdensity-mixed.png]

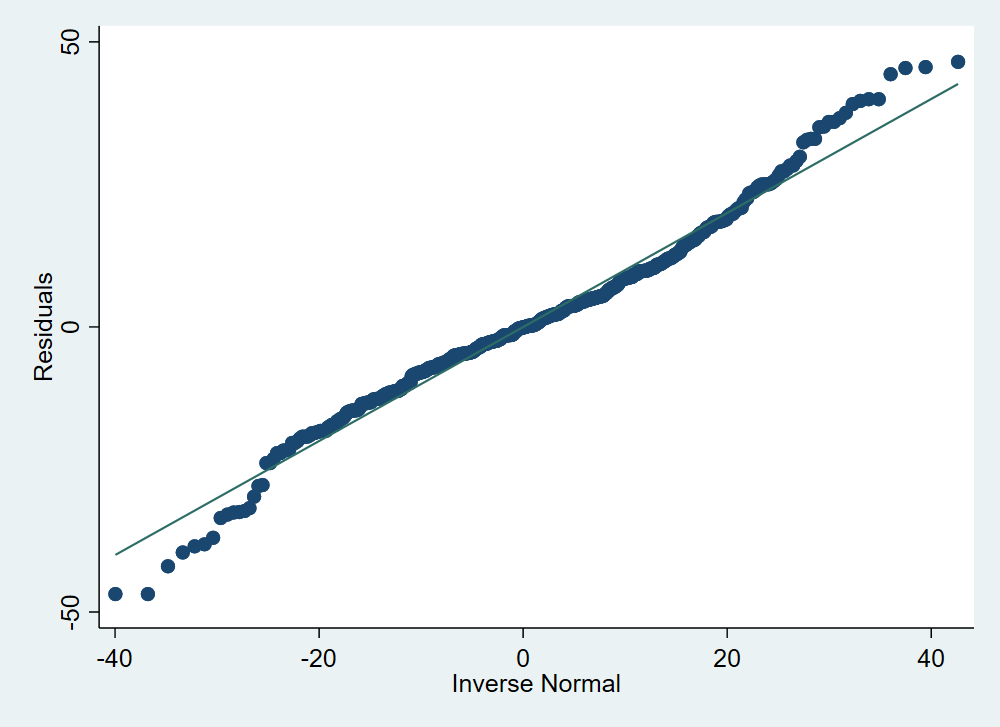

Supplement: Supplementary file 2 — Supplementary Material 2. [file 12891_2024_7312_MOESM2_ESM.zip › saroa-cta-02b-outcome-mixed-rm_ar0_bpas-qnorm-12.png]

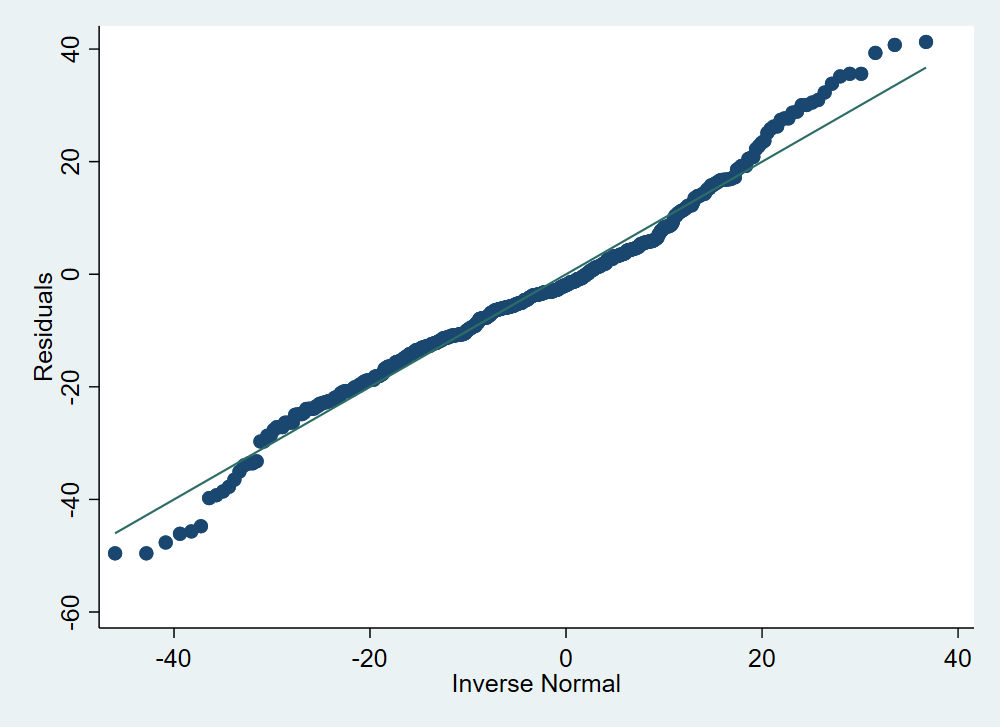

Supplement: Supplementary file 2 — Supplementary Material 2. [file 12891_2024_7312_MOESM2_ESM.zip › saroa-cta-02b-outcome-mixed-rm_ar0_bpas-qnorm-24.png]

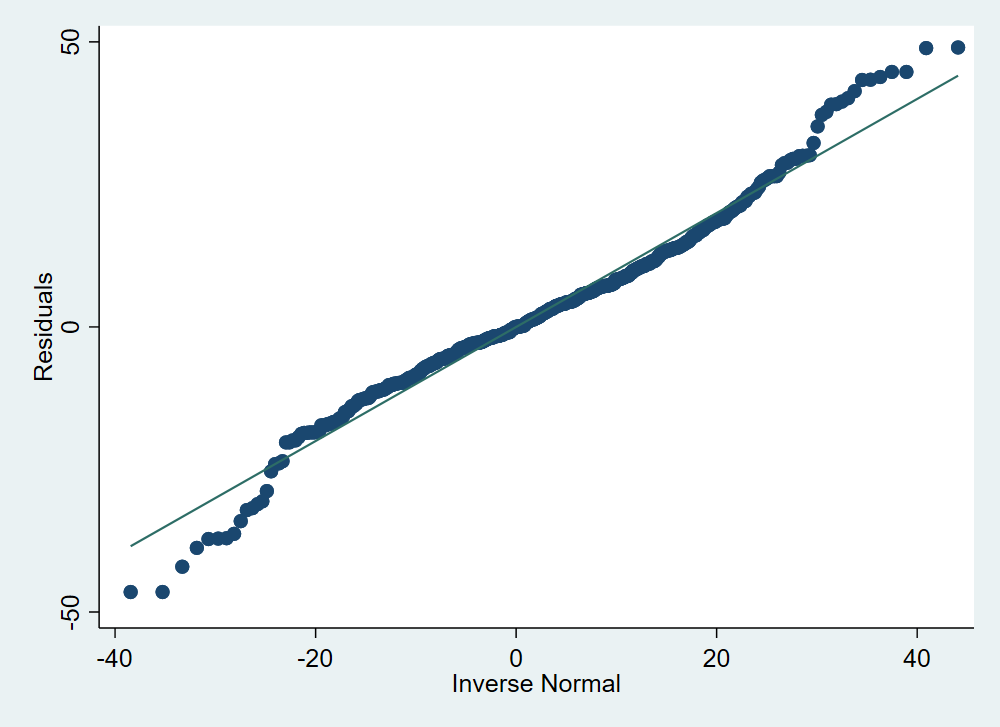

Supplement: Supplementary file 2 — Supplementary Material 2. [file 12891_2024_7312_MOESM2_ESM.zip › saroa-cta-02b-outcome-mixed-rm_ar0_bpas-qnorm-6.png]

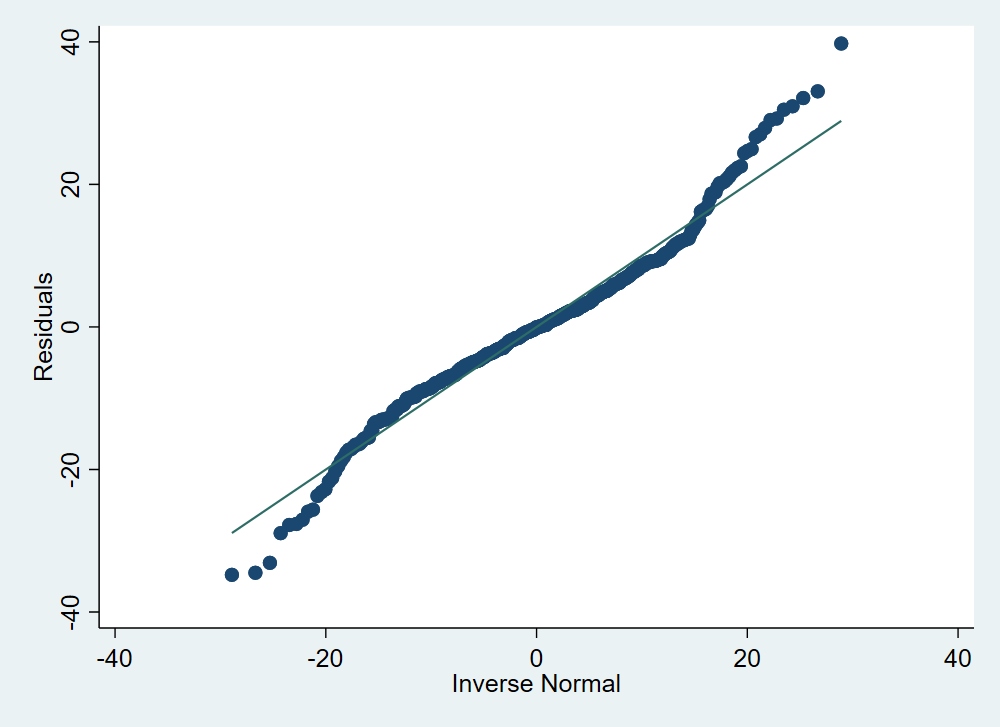

Supplement: Supplementary file 2 — Supplementary Material 2. [file 12891_2024_7312_MOESM2_ESM.zip › saroa-cta-02b-outcome-mixed-rm_ar0_bpas-qnorm-mixed.png]

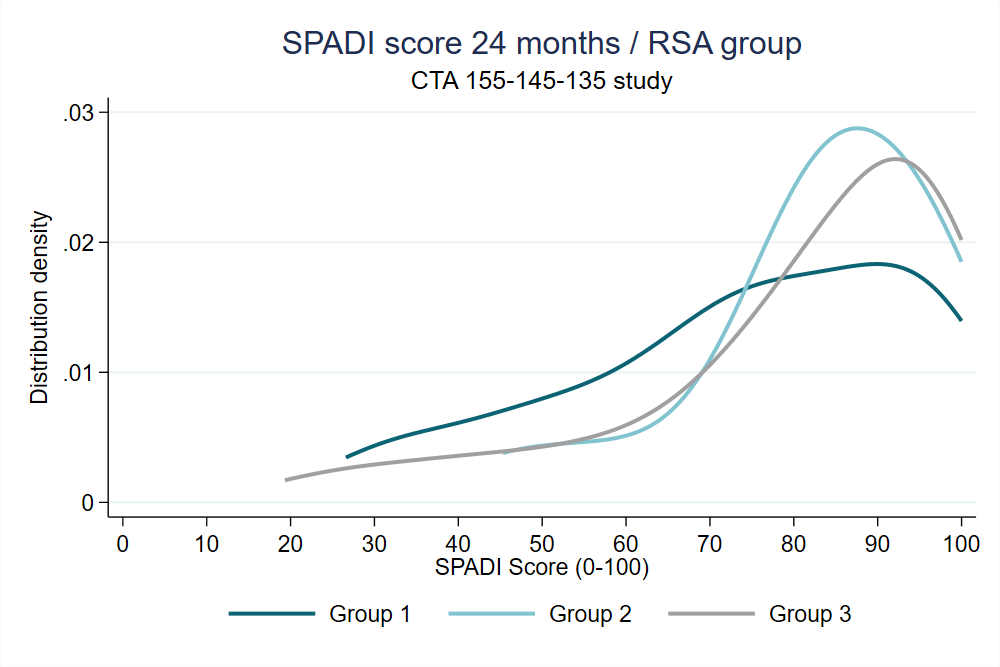

Supplement: Supplementary file 2 — Supplementary Material 2. [file 12891_2024_7312_MOESM2_ESM.zip › saroa-cta-02b-outcome-mixed-spadi-KdensCeiling24mo.png]

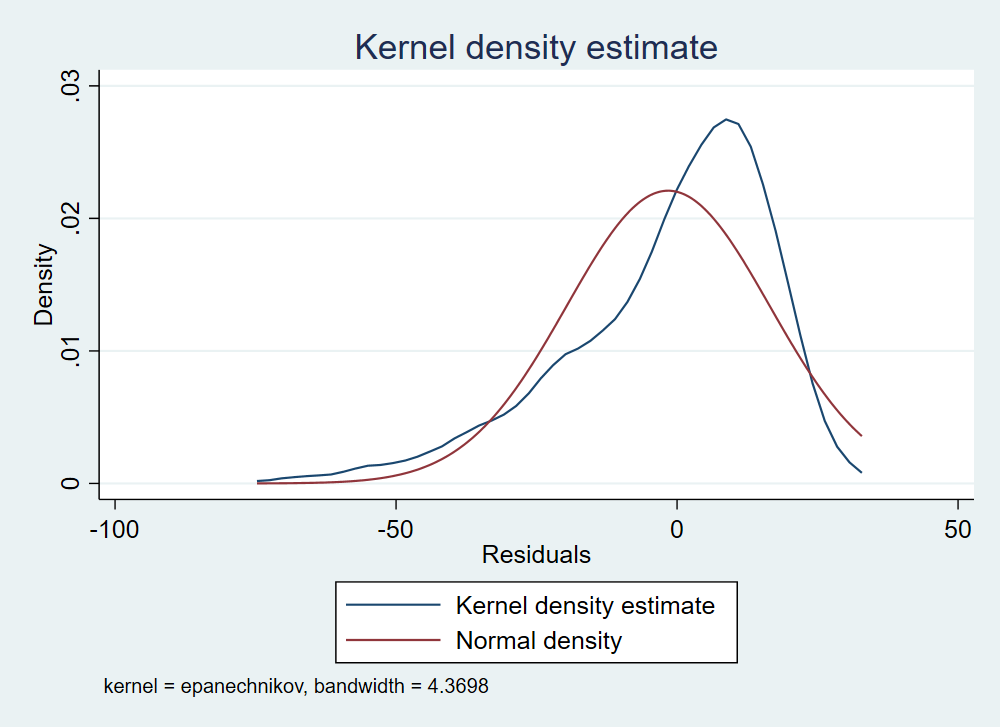

Supplement: Supplementary file 2 — Supplementary Material 2. [file 12891_2024_7312_MOESM2_ESM.zip › saroa-cta-02b-outcome-mixed-spadi-kdensity-12.png]

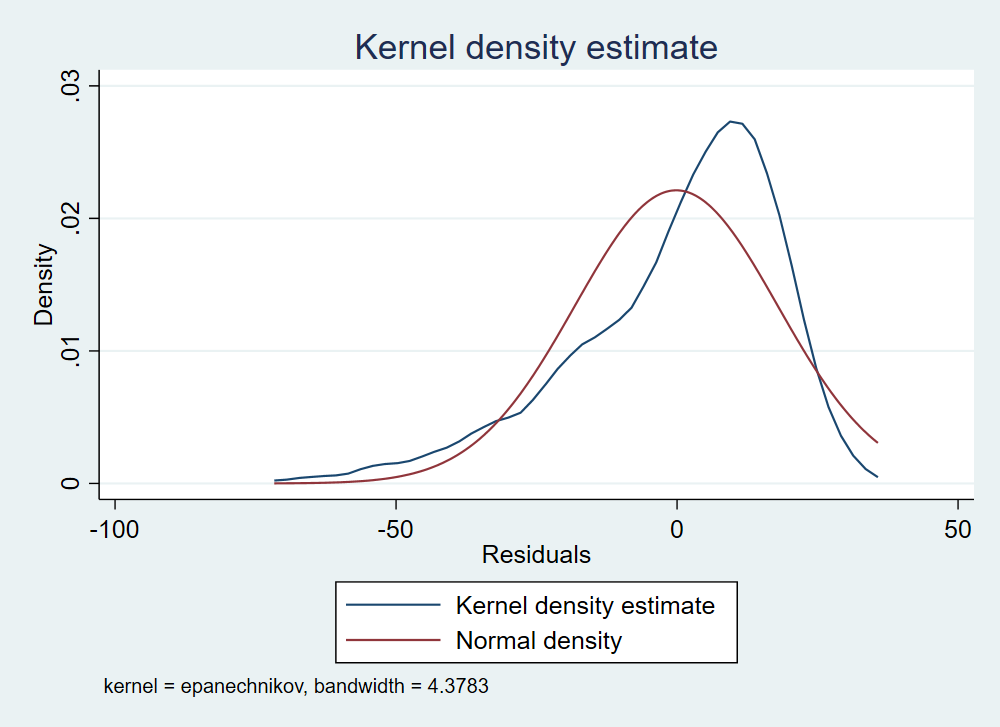

Supplement: Supplementary file 2 — Supplementary Material 2. [file 12891_2024_7312_MOESM2_ESM.zip › saroa-cta-02b-outcome-mixed-spadi-kdensity-24.png]

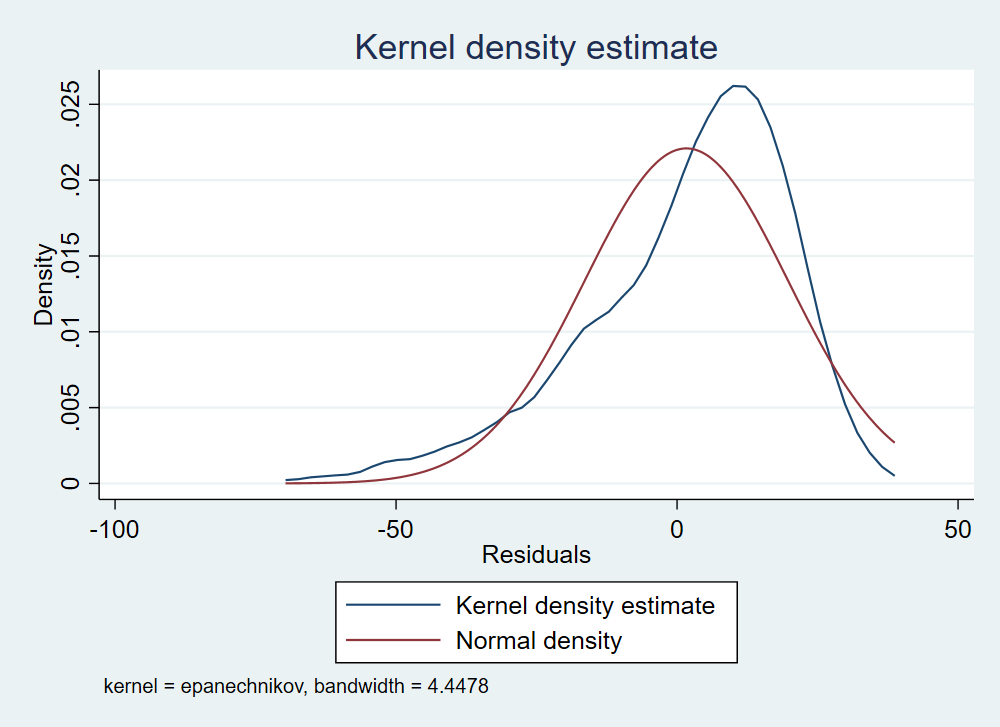

Supplement: Supplementary file 2 — Supplementary Material 2. [file 12891_2024_7312_MOESM2_ESM.zip › saroa-cta-02b-outcome-mixed-spadi-kdensity-6.png]

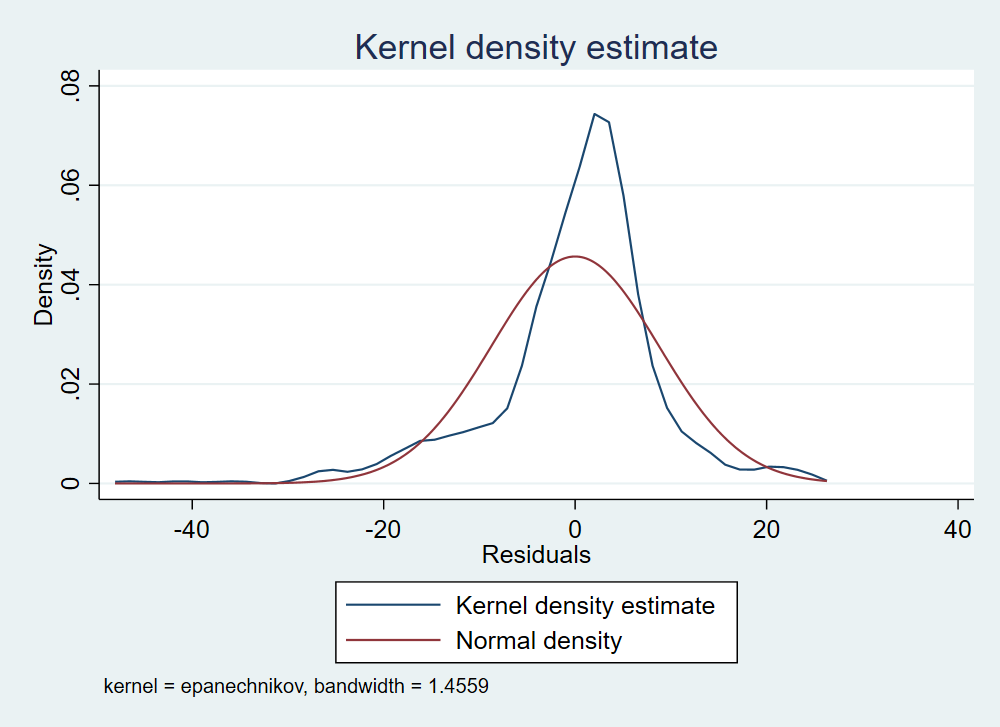

Supplement: Supplementary file 2 — Supplementary Material 2. [file 12891_2024_7312_MOESM2_ESM.zip › saroa-cta-02b-outcome-mixed-spadi-kdensity-mixed.png]

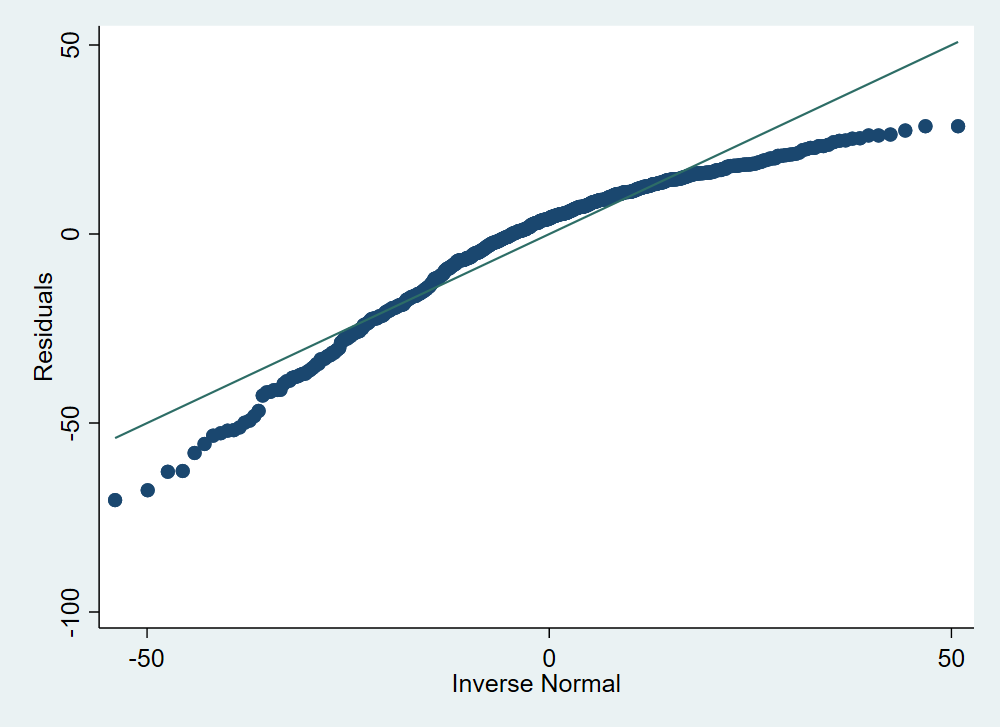

Supplement: Supplementary file 2 — Supplementary Material 2. [file 12891_2024_7312_MOESM2_ESM.zip › saroa-cta-02b-outcome-mixed-spadi-qnorm-12.png]

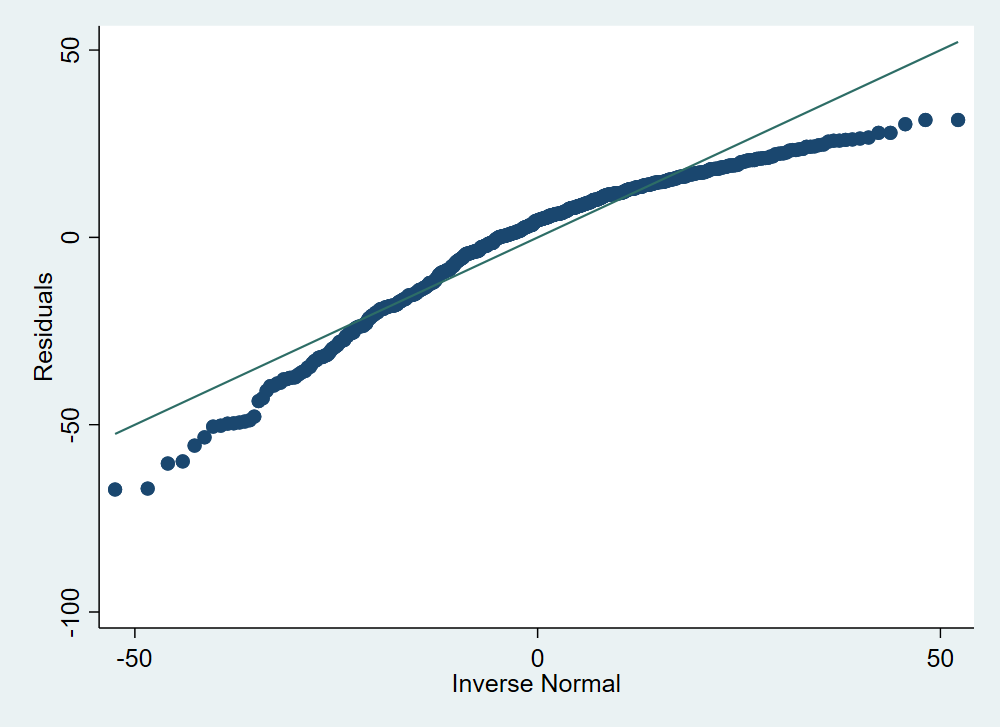

Supplement: Supplementary file 2 — Supplementary Material 2. [file 12891_2024_7312_MOESM2_ESM.zip › saroa-cta-02b-outcome-mixed-spadi-qnorm-24.png]

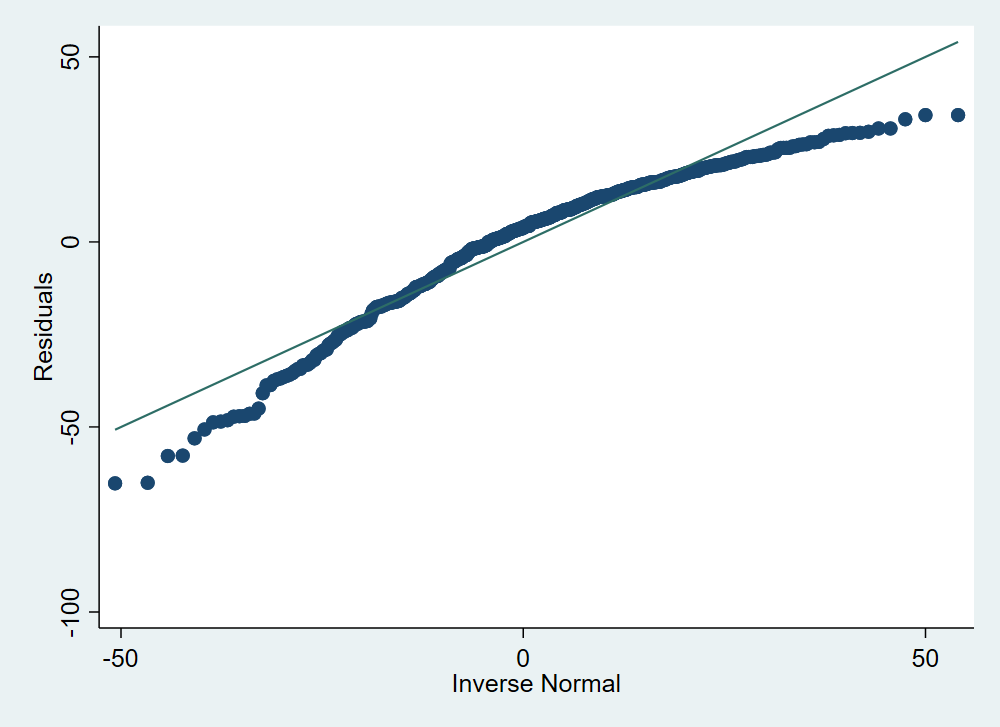

Supplement: Supplementary file 2 — Supplementary Material 2. [file 12891_2024_7312_MOESM2_ESM.zip › saroa-cta-02b-outcome-mixed-spadi-qnorm-6.png]

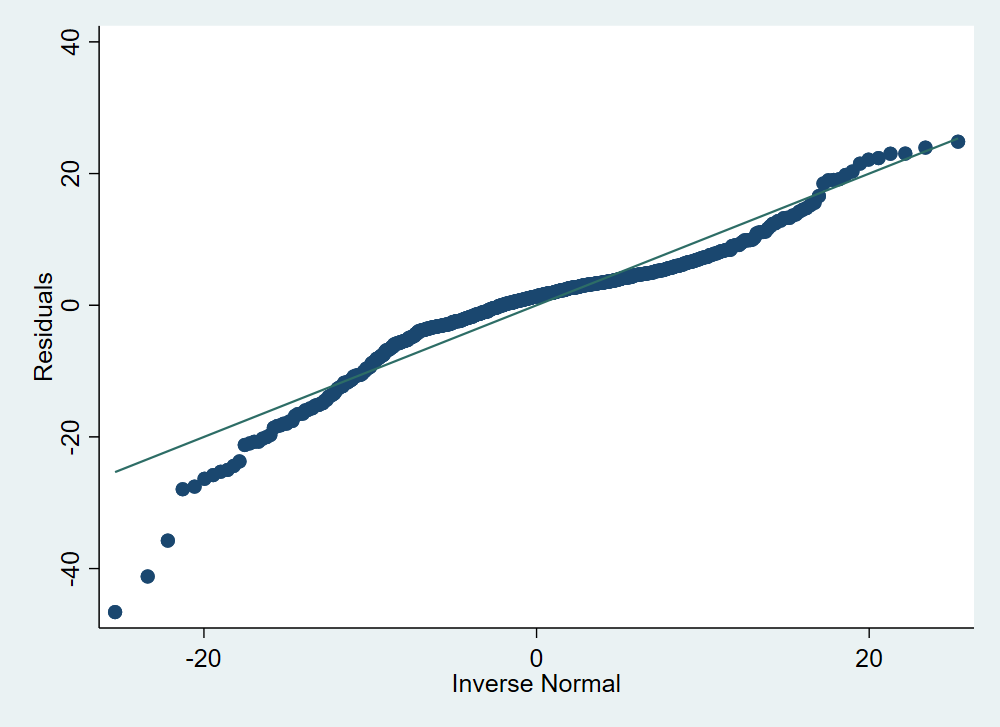

Supplement: Supplementary file 2 — Supplementary Material 2. [file 12891_2024_7312_MOESM2_ESM.zip › saroa-cta-02b-outcome-mixed-spadi-qnorm-mixed.png]

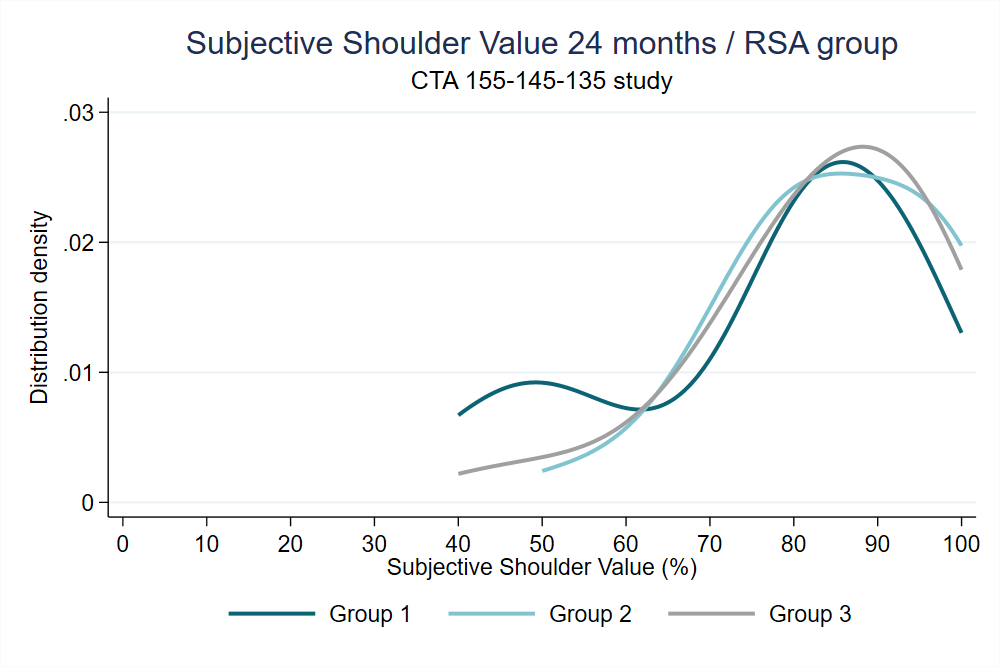

Supplement: Supplementary file 2 — Supplementary Material 2. [file 12891_2024_7312_MOESM2_ESM.zip › saroa-cta-02b-outcome-mixed-ssv-KdensCeiling24mo.png]

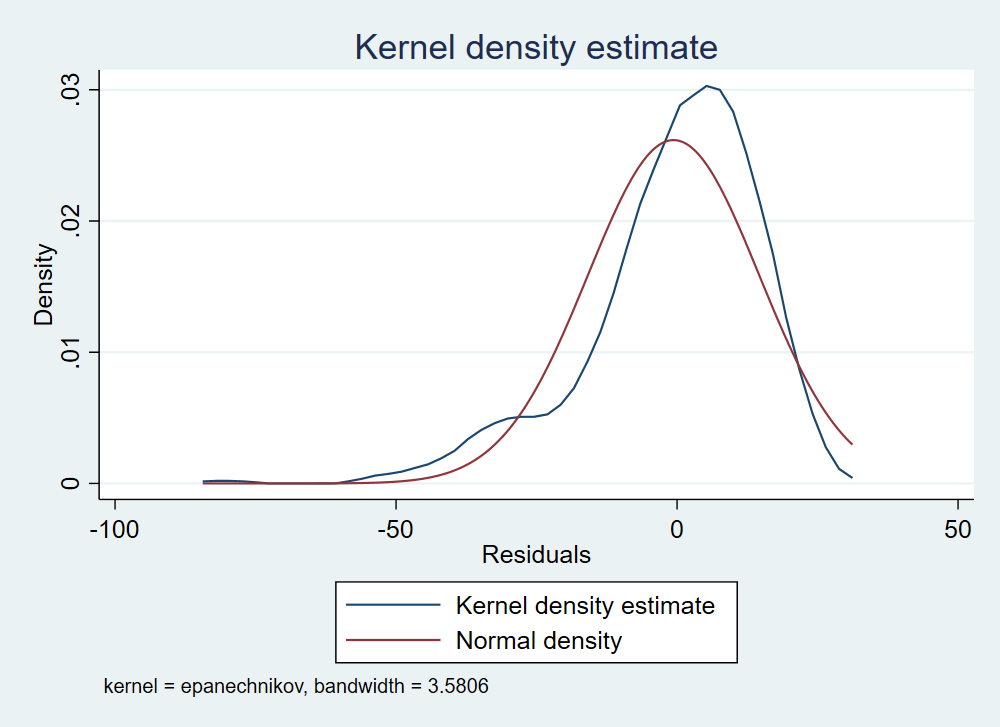

Supplement: Supplementary file 2 — Supplementary Material 2. [file 12891_2024_7312_MOESM2_ESM.zip › saroa-cta-02b-outcome-mixed-ssv-kdensity-12.png]

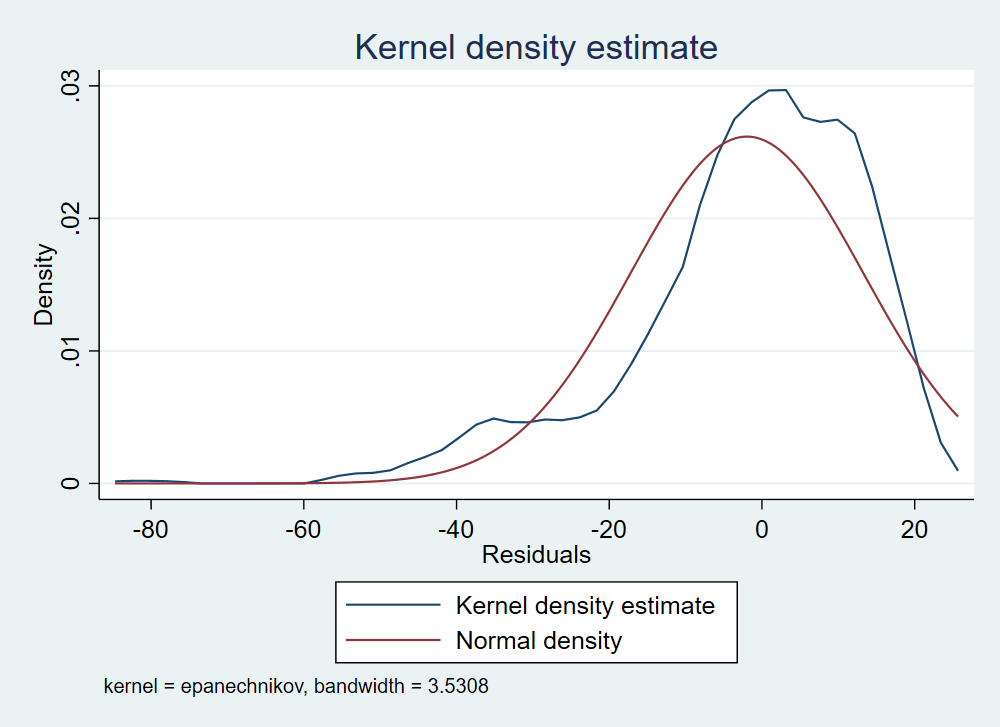

Supplement: Supplementary file 2 — Supplementary Material 2. [file 12891_2024_7312_MOESM2_ESM.zip › saroa-cta-02b-outcome-mixed-ssv-kdensity-24.png]

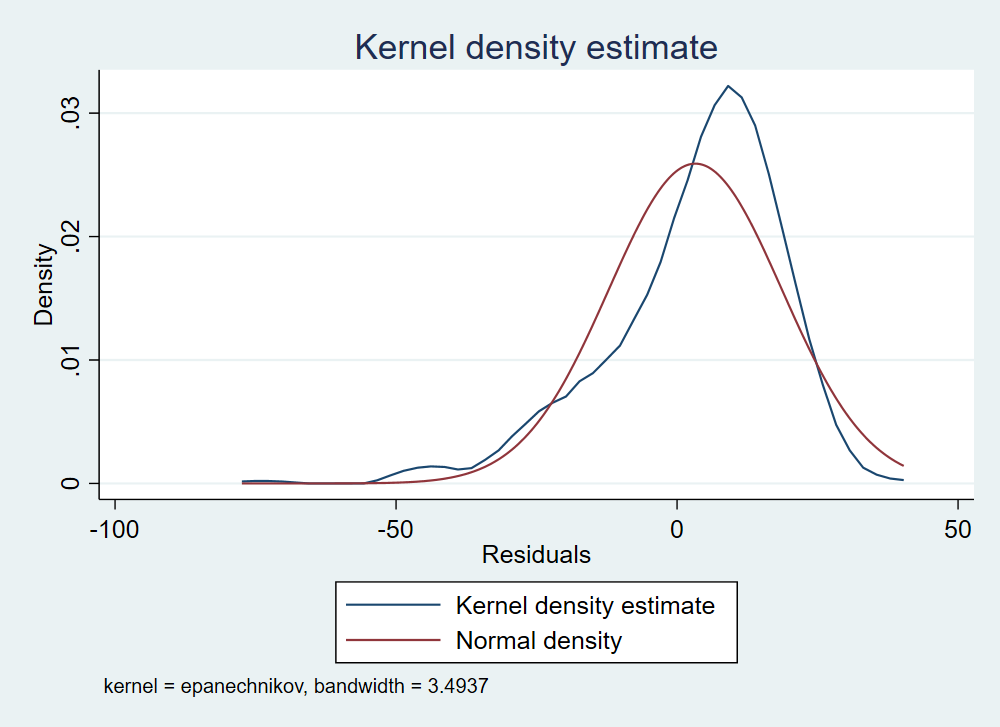

Supplement: Supplementary file 2 — Supplementary Material 2. [file 12891_2024_7312_MOESM2_ESM.zip › saroa-cta-02b-outcome-mixed-ssv-kdensity-6.png]

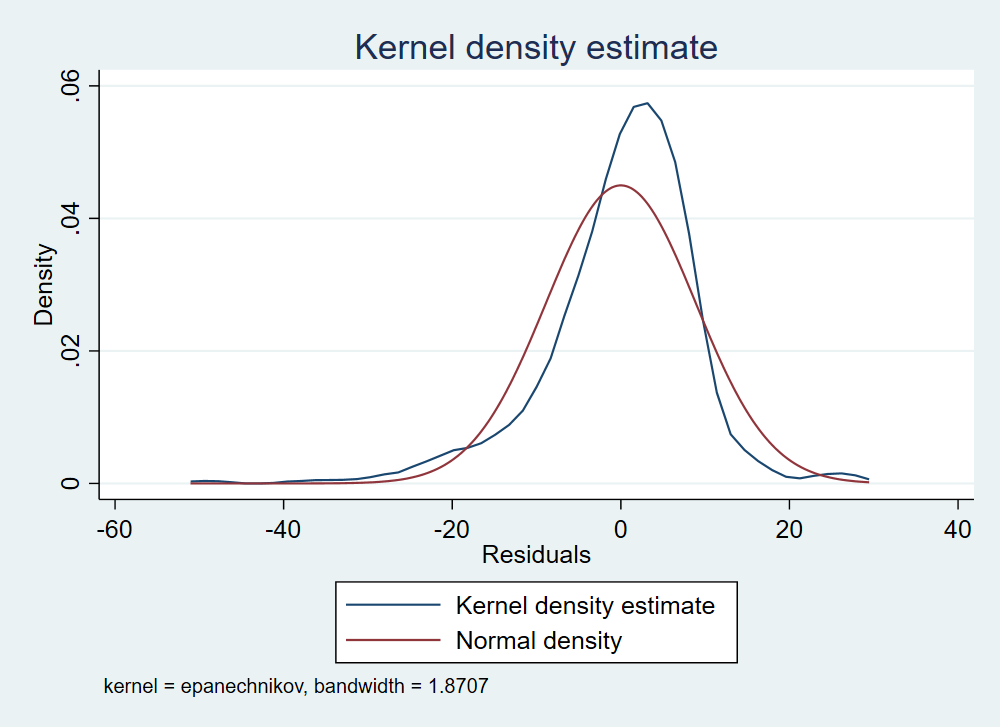

Supplement: Supplementary file 2 — Supplementary Material 2. [file 12891_2024_7312_MOESM2_ESM.zip › saroa-cta-02b-outcome-mixed-ssv-kdensity-mixed.png]

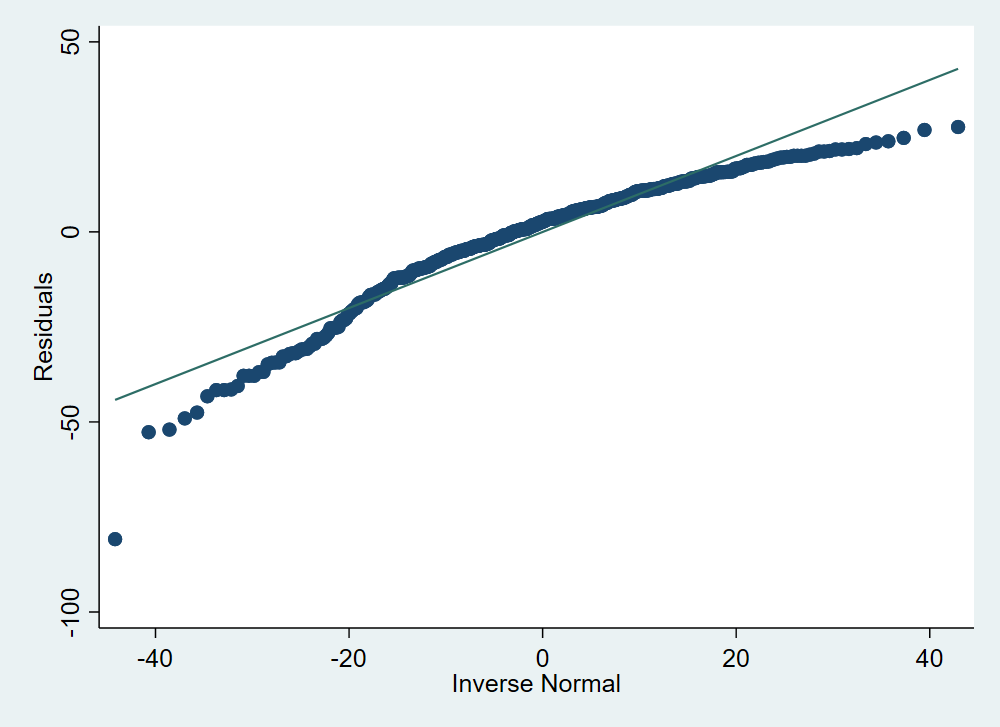

Supplement: Supplementary file 2 — Supplementary Material 2. [file 12891_2024_7312_MOESM2_ESM.zip › saroa-cta-02b-outcome-mixed-ssv-qnorm-12.png]

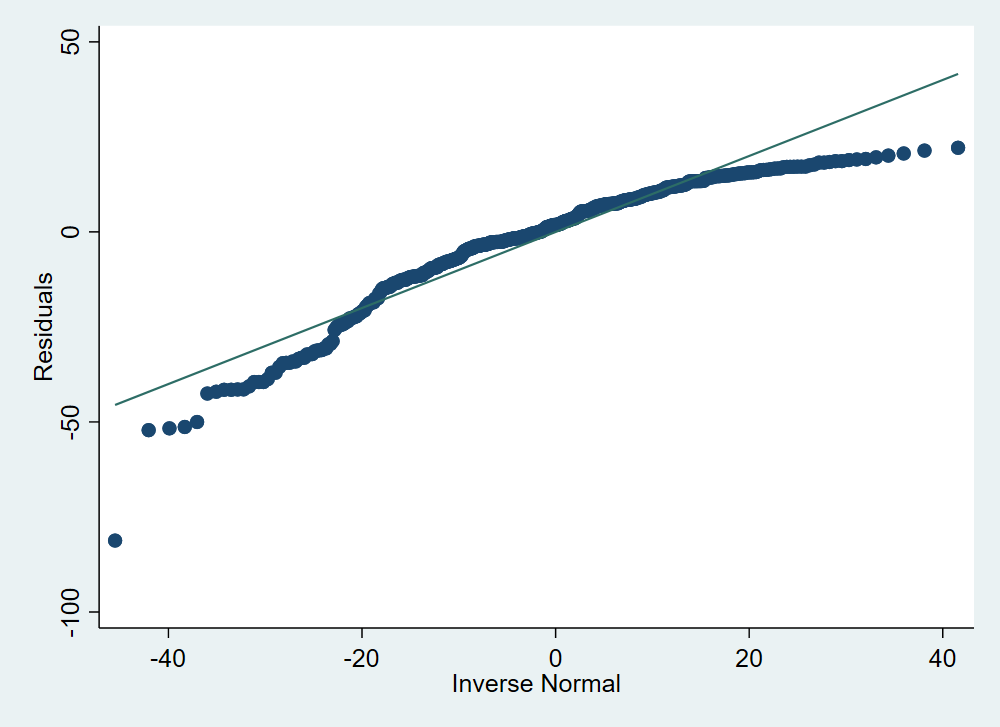

Supplement: Supplementary file 2 — Supplementary Material 2. [file 12891_2024_7312_MOESM2_ESM.zip › saroa-cta-02b-outcome-mixed-ssv-qnorm-24.png]

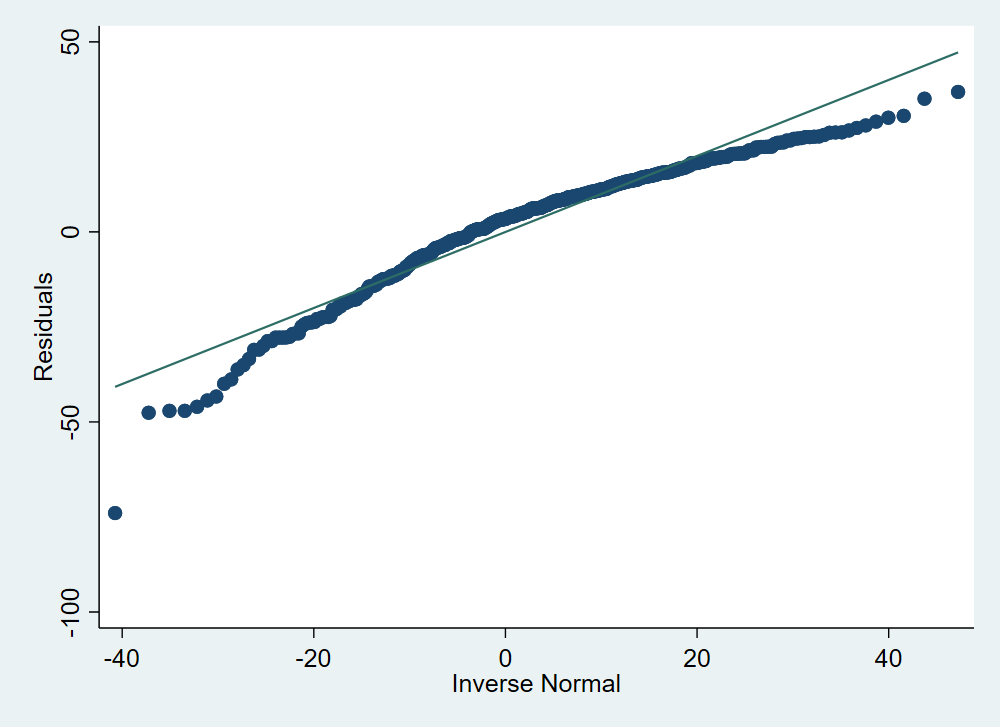

Supplement: Supplementary file 2 — Supplementary Material 2. [file 12891_2024_7312_MOESM2_ESM.zip › saroa-cta-02b-outcome-mixed-ssv-qnorm-6.png]

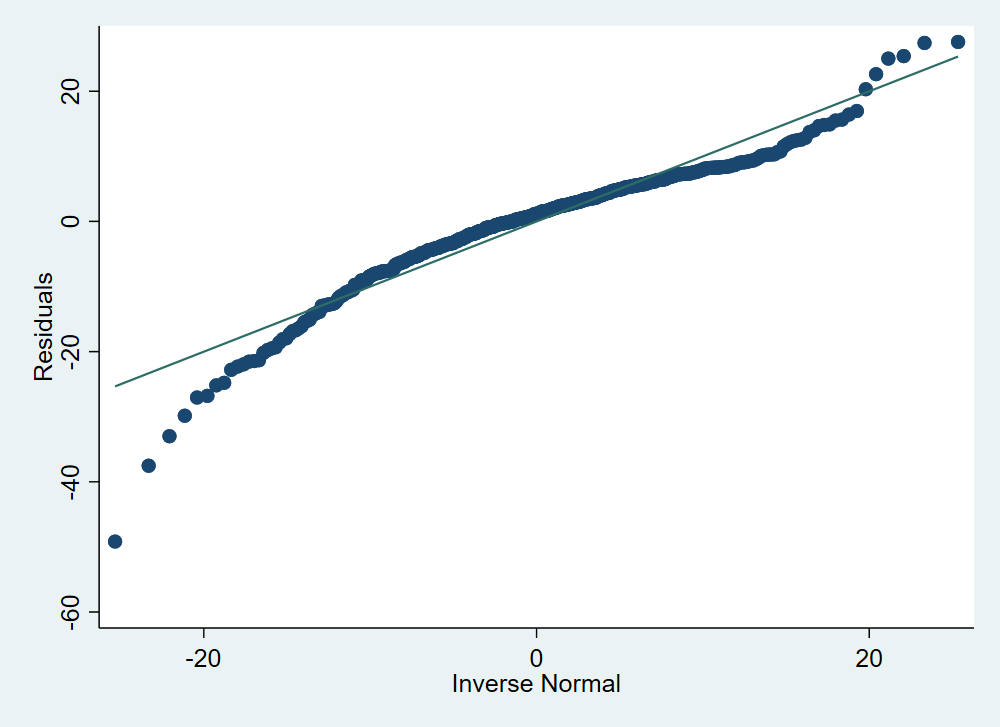

Supplement: Supplementary file 2 — Supplementary Material 2. [file 12891_2024_7312_MOESM2_ESM.zip › saroa-cta-02b-outcome-mixed-ssv-qnorm-mixed.png]
